# Genome-wide association study identifies 8p21.3 associated with persistent hepatitis B virus infection among Chinese

**DOI:** 10.1038/ncomms11664

**Published:** 2016-05-31

**Authors:** Yuanfeng Li, Lanlan Si, Yun Zhai, Yanling Hu, Zhibin Hu, Jin-Xin Bei, Bobo Xie, Qian Ren, Pengbo Cao, Fei Yang, Qingfeng Song, Zhiyu Bao, Haitao Zhang, Yuqing Han, Zhifu Wang, Xi Chen, Xia Xia, Hongbo Yan, Rui Wang, Ying Zhang, Chengming Gao, Jinfeng Meng, Xinyi Tu, Xinqiang Liang, Ying Cui, Ying Liu, Xiaopan Wu, Zhuo Li, Huifen Wang, Zhaoxia Li, Bo Hu, Minghui He, Zhibo Gao, Xiaobing Xu, Hongzan Ji, Chaohui Yu, Yi Sun, Baocai Xing, Xiaobo Yang, Haiying Zhang, Aihua Tan, Chunlei Wu, Weihua Jia, Shengping Li, Yi-Xin Zeng, Hongbing Shen, Fuchu He, Zengnan Mo, Hongxing Zhang, Gangqiao Zhou

**Affiliations:** 1 State Key Laboratory of Proteomics, Beijing Proteome Research Center, Beijing Institute of Radiation Medicine, Beijing 100850, China; 2 National Engineering Research Center for Protein Drugs, Beijing 100850, China; 3 National Center for Protein Sciences at Beijing, Beijing 100850, China; 4 Center for Genomic and Personalized Medicine, Guangxi Medical University, Nanning, Guangxi 530021, China; 5 Department of Epidemiology and Biostatistics, MOE Key Laboratory of Modern Toxicology, School of Public Health, Nanjing Medical University, Nanjing 210029, China; 6 State Key Laboratory of Oncology in Southern China, Guangzhou 510060, China; 7 Affiliated Cancer Hospital of Guangxi Medical University, Nanning, Guangxi 530021, China; 8 National Laboratory of Medical Molecular Biology, Institute of Basic Medical Sciences, Chinese Academy of Medical Sciences, Beijing 100005, China; 9 Department of Infectious Disease, Affiliated You'an Hospital, Capital University of Medical Science, Beijing 100069, China; 10 Liver Failure Treatment and Research Center, Beijing 302 Hospital, Beijing 100039, China; 11 Department of Clinical Laboratory, the Third Affiliated Hospital, Sun Yat-sen University, Guangzhou 510630, China; 12 BGI-Shenzhen, Shenzhen 518031, China; 13 Department of Gastroenterology and Hepatology, Jinling Hospital, Clinical School of Nanjing University, Nanjing 210002, China; 14 Department of Gastroenterology, the First Affiliated Hospital, College of Medicine, Zhejiang University, Hangzhou 310006, China; 15 Department of Hepatobiliary Surgery I, Peking University Cancer Hospital and Institute, Beijing 100142, China

## Abstract

Hepatitis B virus (HBV) infection is a common infectious disease. Here we perform a genome-wide association study (GWAS) among Chinese populations to identify novel genetic loci involved in persistent HBV infection. GWAS scan is performed in 1,251 persistently HBV infected subjects (PIs, cases) and 1,057 spontaneously recovered subjects (SRs, controls), followed by replications in four independent populations totally consisting of 3,905 PIs and 3,356 SRs. We identify a novel locus at 8p21.3 (index rs7000921, odds ratio=0.78, *P*=3.2 × 10^−12^). Furthermore, we identify significant expression quantitative trait locus associations for *INTS10* gene at 8p21.3. We demonstrate that INST10 suppresses HBV replication via IRF3 in liver cells. In clinical plasma samples, we confirm that INST10 levels are significantly decreased in PIs compared with SRs, and negatively correlated with the HBV load. These findings highlight a novel antiviral gene *INTS10* at 8p21.3 in the clearance of HBV infection.

## Abstract (web summary)

This genome-wide association study on persistent hepatitis B virus (HBV) infection among Chinese confirms previously associated genetic loci while discovering a novel protective locus at 8p21.3. The study also demonstrates the nearby gene *INST10* suppresses HBV replication *in vitro*.

## Graphical abstract

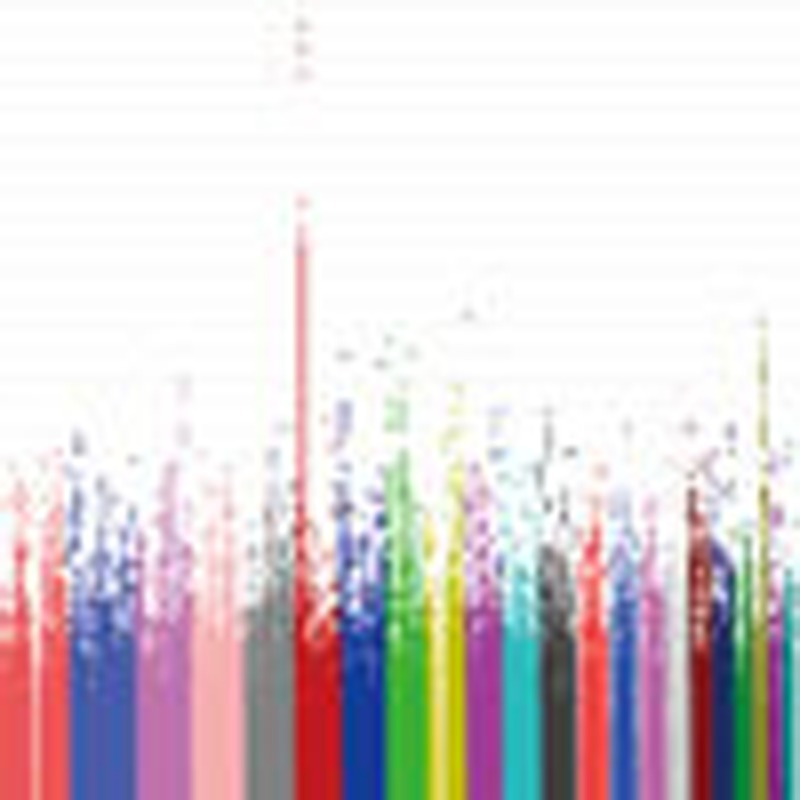

Hepatitis B virus (HBV) infection is one of the major infectious diseases with >250 million chronic carriers worldwide, causing a broad spectrum of liver diseases ranging from asymptomatic carrier, fulminant hepatitis, chronic hepatitis and liver cirrhosis to hepatocellular carcinoma (HCC)^1^. There is an urgent health care need to understand and control chronic HBV infection. Persistent HBV infection or HBV clearance has been considered a multifactorial and polygenic event with viral, environmental and genetic components^2^,^3^. The segregation analyses and twin studies strongly supported the roles of host genetic factors in determining the persistence of HBV infection^4^,^5^. Recently, several genome-wide association studies (GWASs) have identified single nucleotide polymorphisms (SNPs) at eight loci linking genetic susceptibility to persistent HBV infection in populations of Asia ancestry, including *HLA-DP* (index rs3077 and rs9277535), *HLA-DQ* (rs2856718 and rs7453920), *HLA-C* (rs3130542), *EHMT2* (rs652888), *TCF19* (rs1419881), *CFB* (rs12614) and two non-HLA loci *UBE2L3* (rs4821116) and *CD40* (rs1883832) (refs 6, 7, 8, 9, 10). However, in some of these studies, due to relatively small sample size or the unknown history of HBV exposure in the control subjects, the power to detect the low-penetrance loci with modest effects could decrease dramatically. Furthermore, the susceptibility to infectious diseases is considered to be determined at different functional levels^11^, suggesting additional genetic factors remain to be discovered.

To identify new loci conferring susceptibility to persistent HBV infection among Chinese, here we conduct a GWAS consisting of 1,251 persistently HBV infected subjects (persistently infected (PIs); cases) and 1,057 spontaneously recovered subjects (spontaneously recovered (SRs); controls), followed by validation of top candidate SNPs in four independent sample sets totally including 3,905 PIs and 3,356 SRs. The study confirms previously associated genetic loci while discovering a novel protective locus at 8p21.3 (index SNP rs7000921). By expression quantitative trait locus (eQTL) analyses and functional studies, we further demonstrates the nearby gene integrator complex subunit 10 (*INTS10*) at 8p21.3 suppresses HBV replication in an interferon regulatory factor 3 (IRF3)-dependent manner *in vitro*. In summary, the GWAS identifies a novel antiviral gene *INTS10* at 8p21.3 in the clearance of HBV infection.

## Results

### Genome-wide association analyses

To detect novel loci conferring susceptibility to persistent HBV infection, we carried out a two-stage GWAS (Supplementary Fig. 1). In the discovery GWAS stage, we used genotypes from 12,027 individuals by various genotyping platforms providing genome-wide coverage (Table 1 and Supplementary Note)^12^,^13^,^14^,^15^. With the plasma/serum of these subjects available, we determined who of them were PIs (cases) or SRs (controls) by screening for hepatitis B surface antigen (HBsAg), and antibodies against HBsAg (anti-HBs) and hepatitis B core antigen (anti-HBc). Totally, 1,251 cases and 1,057 controls were involved in the GWAS stage, all of whom are of Chinese ancestry recruited from Guangxi, Guangdong and Jiangsu provinces, respectively (Table 1, Supplementary Table 1a and Supplementary Note). In the replication stage, four independent sample sets of Chinese ancestry that were recruited from Jiangsu, Guangxi, Guangdong and Beijing provinces, respectively, were included (Supplementary Note). With the same sample inclusion and exclusion criteria as those used in the discovery GWAS stage, the replication stage totally consisted of 3,905 cases and 3,356 controls (Table 1 and Supplementary Table 1a)^16^,^17^.

To extend the coverage to the genomic region in the GWAS stage, we used genotypes of autosomal SNPs that passed strict quality checks to impute genotypes of SNPs across the chromosomes for all subjects (Methods section and Supplementary Table 2). We performed three rounds of imputation using the data from the HapMap project phase II, HapMap project phase III and the 1000 Genomes Project as references, respectively, and generated genotypes of 2,177,782, 1,059,015 and 4,494,311 SNPs, respectively (Supplementary Table 3). To assess the accuracy of genotyping and imputation, we resequenced a ∼127-Kb genomic region at 1p36.22 in 274 subjects randomly selected from the GWAS stage (Supplementary Note). Excellent concordance between the array genotyping and sequencing was observed in these individuals (98.6%; *P*<2.2 × 10^−16^, Kappa test; Supplementary Table 4). A high consistency between the imputation and sequencing was also observed (Pearson's correlation *r*=0.94, *P*<2.2 × 10^−16^; Supplementary Fig. 2a). Moreover, we noted that the SNPs with high imputation quality (imputation *r*^*2*^>0.8) showed a higher consistency between the imputation and sequencing than those with low imputation quality (Pearson's correlation *r*=0.95 and 0.86, respectively; Supplementary Fig. 2b,c).

Having shown the validity of array genotyping and imputation data in the GWAS stage, we then carried out genotype–phenotype association analyses using non-integer allele numbers in logistic regression model, with adjustment for age, sex and principal components-based correction for population stratification (Methods section and Supplementary Fig. 3). A quantile–quantile plot showed a good match between the distributions of observed *P* values and those expected by chance (inflation factor *λ*=1.05; Supplementary Fig. 4), indicating minimal overall inflation of the genome-wide statistical results.

### Several previously reported SNPs were replicated

Recent GWASs have identified a number of SNPs that were significantly associated with the risk of persistent HBV infection. In this study, we confirmed the genetic effects of *HLA-DP* (index rs9277535, *P*=3.8 × 10^−6^; and rs3077, *P*=2.3 × 10^−3^), *HLA-DQ* (rs2856718, *P*=1.8 × 10^−3^; and rs7453920, *P*=5.5 × 10^−6^), *CFB* (rs12614, *P*=4.0 × 10^−3^) and *CD40* (rs1883832, *P*=6.9 × 10^−3^) (Supplementary Table 5), which have been identified in previous GWASs (refs 6, 7, 10). However, other four SNPs (rs652888, rs1419881, rs3130542 and rs4821116) in or near *EHMT2*, *TCF19*, *HLA-C* and *UBE2L3* loci^8^,^9^ failed to be replicated in this study (all *P*>0.05; Supplementary Table 5). These results were unlikely to be caused by the error of imputation, as these four SNPs were either directly genotyped or imputed with high imputation quality (imputation *r*^*2*^>0.96). We also reviewed the previous candidate gene-based association studies of persistent HBV infection. In addition to the SNPs in *HLA-DP* and *HLA-DQ*, the SNPs in the microRNA gene *MIR219A1* at 6p21.32 (ref. 18) were also be replicated (*P*=2.6 × 10^−6^ and 1.3 × 10^−6^ for rs421446 and rs107822, respectively; Supplementary Table 6). However, the other previously reported SNPs did not show any consistent associations in this study (Supplementary Table 6). These inconsistent associations between our study and the previous studies may be due to the different study design or racial diversity.

To further explore the associations between the *HLA* classical alleles and persistent HBV infection, we performed *HLA* allele genotyping *in silico* on the basis of the known SNPs genotypes (Supplementary Note) using the R package HIBAG (ref. 19). Four previously identified alleles (HLA-DPB1*201, HLA-DQA1*301, HLA-DQB*301 and HLA-DQB*302) were replicated in the present study (all *P*<5 × 10^−3^; Supplementary Table 7) (ref. 9). In addition, we newly identified the allele HLA-DPB1*501 (*P*=4.0 × 10^−4^), which was in moderate linkage disequilibrium (LD) with the previously identified SNPs rs3077 (*r*^*2*^=0.30) and rs9277535 (*r*^*2*^=0.56) (Supplementary Table 7).

Recent GWASs have also identified several SNPs that are associated with HBV-related liver phenotypes. Among those SNPs, several ones in *HLA-DP* and *HLA-DQ* that were significantly associated with hepatitis B vaccine response or HBV-related HCC also showed suggestive associations with persistent HBV infection (Supplementary Table 8), reflecting shared genetic risk factors among the HBV-related phenotypes. However, all the other SNPs showed no associations with persistent HBV infection in our GWAS data (Supplementary Table 8), suggesting that the molecular mechanisms among these phenotypes are largely different.

### A new susceptibility locus at 8p21.3 was identified

**Figure 1:**
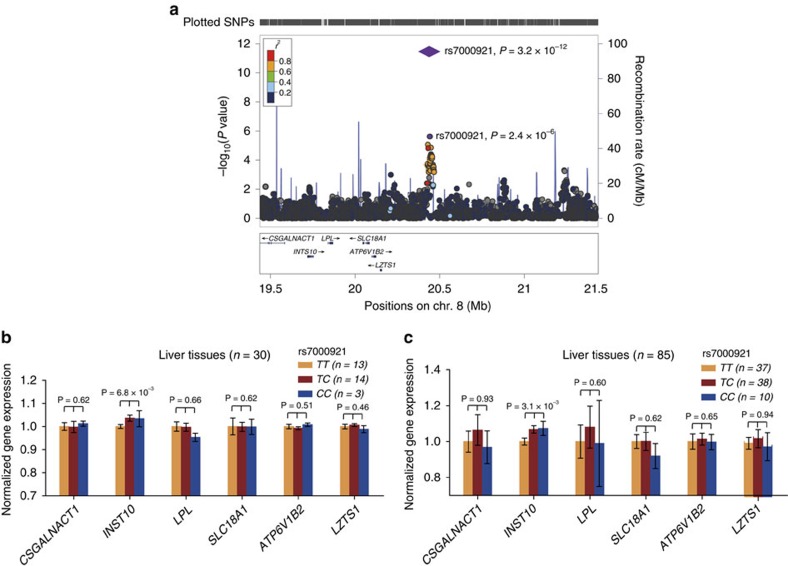
rs7000921 are significantly associated with persistent HBV infection and rs7000921 is associated with the mRNA levels of ***INST10*****in liver tissues.** (**a**) The genetic association results were shown for SNPs in the region 1-Mb up- or downstream of the index SNP rs7000921. Genomic positions are based on NCBI Build 36. In the meta-analysis, the *P* value of rs7000921 is shown as purple diamonds, with their initial *P* value in the GWAS stage shown as purple dots. The LD values (*r*^2^) to rs7000921 for the other SNPs are indicated by marker colour. Red signifies *r*^*2*^≥0.8, orange 0.6≤*r*^*2*^<0.8, green 0.4≤*r*^*2*^<0.6, light blue 0.2≤*r*^*2*^<0.4 and blue *r*^*2*^<0.2. Estimated recombination rates (from the HapMap project phase II) are plotted in light blue. Genes within the region surrounding rs7000921 are annotated, with the positions of transcripts shown by arrows. (**b**,**c**) The mRNA expression levels of nearby genes in subjects with different rs7000921 genotypes (*CC*, *CT* and *TT*) were shown. The mRNA expression levels were log2 transformed. Expression levels of each gene were normalized to the mean level of homozygotes for the major allele of rs7000921 (*TT* genotype) in 31 (**b**) or 88 (**c**) human liver tissue samples. Among the 31 liver samples, one sample failed to be genotyped for the rs7000921, thus the analyses were only restricted in the remaining 30 samples. Among the 88 liver samples, three subjects were considered as outliers (their mRNA levels of *INTS10*>mean+3 s.d. or <mean −3 s.d.), thus the analyses were restricted in the remaining 85 samples. *P* values were derived from linear regression analyses, and were considered to be significant when below 0.05 after Bonferroni correction by multiplying the number of comparisons. Error bars indicate s.e.m.

In addition to the previously reported SNPs in *HLA-DP*, *HLA-DQ* and *MIR219A1*, seventy-two loci showed significant associations with *P*≤1 × 10^−4^ in the discovery GWAS stage in this study. We then selected all of these top 72 signals for replication (Supplementary Data 1 and Supplementary Table 9; Methods section) in an independent sample set (replication stage 1, Jiangsu population). Of these 72 tested SNPs, 6 SNPs showed significant associations in the same direction as observed in the GWAS stage (Supplementary Data 1). These 6 SNPs were further genotyped in another sample set (replication stage 2, Guangxi population), and only rs7000921 at 8p21.3 were replicated (Supplementary Data 1). Consistently, rs7000921 showed evidence of association in replication stage 3 (Guangdong population) and stage 4 (Beijing population; Supplementary Data 1). In the combined analyses, rs7000921 (odds ratio (OR)=0.78, *P*_meta_=3.2 × 10^−12^) reached genome-wide significance for association with persistent HBV infection (Fig. 1a, Table 2 and Supplementary Fig. 5). No evidence of heterogeneity for OR values of rs7000921 was observed among all these sample sets (*P*_heterogeneity_=0.29; Table 2).

We further investigated the effect of rs7000921 on persistent HBV infection using stratification by sex and age. In the pooled case–control samples, we found no appreciable variation of the effects across the subgroups stratified by age or sex for rs7000921 (*P*_heterogeneity_=0.088 and 0.26, respectively; Supplementary Table 10). The interaction effects between rs7000921 and viral factors (for example, HBV genotypes and mutations, and viral load) were not assessed because these data were not fully available in our samples. Therefore, the possibility that the association signals detected by rs7000921 reflect some other aspects of disease biology related to persistent HBV infection risk cannot be completely ruled out.

### *INTS10* was identified as the causative gene at 8p21.3

The SNP rs7000921 is located at intergenic region on chromosome 8p21.3. Six genes (*CSGALNACT1*, *INST10*, *LPL*, *SLC18A1*, *ATP6V1B2* and *LZTS1*) are located within 1 Mb from this SNP (Fig. 1a). To identify potentially causative gene(s) at 8p21.3, we performed eQTL analyses based on liver tissues from 31 patients with persistent HBV infection (Methods section). We found that the protective minor allele C of rs7000921 was significantly associated with elevated transcript levels of *INST10* (*P*=6.8 × 10^−3^; Fig. 1b and Supplementary Data 2). This liver eQTL finding was then replicated in an independent sample set of 88 human liver tissues (*P*=3.1 × 10^−3^; Fig. 1c, Supplementary Data 2 and Methods section)^20^,^21^. In these two sample sets, the expression of *INST10* in protective allele carriers (*TC* or *CC* of rs7000921) showing 22–31% elevation compared with that in risk allele carriers (*TT*; Fig. 1b,c). The associations remained significant even after Bonferroni correction for multiple comparisons. When these two sample sets were pooled together, we achieved a more significant eQTL signal (Fisher's combined *P*=2.5 × 10^−4^). No significant eQTL signals were found between the rs7000921 and the other five genes at 8p21.3. Taken together, these results suggest a potential role for *INTS10* in persistent HBV infection. However, the allele-specific changes of *INST10* expression in liver tissues were not seen in lymphocytes of HapMap populations, suggesting that the underlying regulatory mechanism is tissue-specific.

To investigate candidate causative variants, we performed functional annotation for the genetic variants that are tagged by the index SNP rs7000921 (*r*^*2*^>0.7) on the basis of publically available data sets or tools (Supplementary Note and Supplementary Table 11). All the SNPs highly correlated with rs7000921 are located at intergenic regions, of which rs11991803 (*r*^*2*^=0.739) and rs4922214 (*r*^*2*^=0.729) are in conserved regions predicted to have high regulatory potential scores (Supplementary Table 11). The eQTL analyses of rs11991803 and rs4922214 showed genotype-specific expression of *INST10*, similar to the results of rs7000921 (Supplementary Fig. 6). We further checked the data from the Encyclopedia of DNA Elements (ENCODE) database, and found that the rs11991803 was within a transcriptional repressor CCCTC-binding factor–binding site detected in multiple cell types including the human hepatoma cell line HepG2, suggesting that this variant might be involved in gene regulation (Supplementary Fig. 7). Taken together, these observations suggest that the causative variants at 8p21.3 may have long-range action in the regulation of *INTS10* expression in certain cell types and merit further investigation in the future.

### INTS10 suppresses HBV replication

INTS10 is a subunit of the integrator complex, which can interact with RNA polymerase II to mediate 3′ end processing of small nuclear RNAs U1 and U2, the core components of spliceosome^22^,^23^,^24^. In addition, the integrator complex mediates transcriptional initiation, pause release and transcriptional termination at diverse classes of gene targets, including host small nuclear RNAs and coding genes and viral microRNAs^25^,^26^,^27^. INTS10 is expressed in a wide range of tissue types including in liver tissues, according to the RNA-Seq Atlas database^28^. However, the specific roles of INTS10 in diseases, for example, in persistent HBV infection, remain unclear.

**Figure 2:**
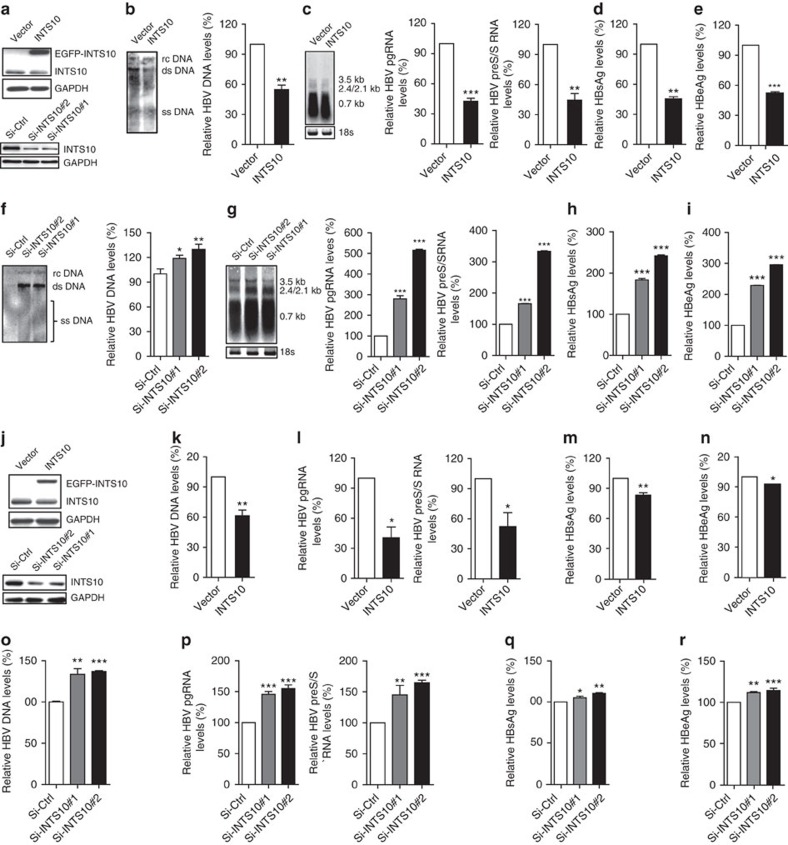
INTS10 suppresses HBV replication in L02 and HepG2.2.15 cells. (**a**) Protein levels of INTS10 in cellular lysates of L02 cells. L02 cells (∼2 × 10^5^) were transfected with pAAV-HBV1.2 vectors, together with pLV-EGFP-INTS10 vector (INTS10) or pLV-EGFP control vector(Vector) (up) or with INTS10-specific siRNAs (Si-INTS10#1 and Si-INTS10#2) or non-targeting scrambled siRNA (Si-Ctrl) (down). (**b**,**c**) Levels of HBV DNAs (**b**), 3.5 Kb pregenomic RNAs (pgRNAs) and 2.4/2.1 Kb Pre-S/S RNAs (**c**) in L02 cells with INTS10 overexpression. (**d**,**e**) Levels of HBsAg (**d**) and HBeAg (**e**) in supernatants of L02 cells with INTS10 overexpression. (**f**,**g**) Levels of HBV DNAs (**f**), pgRNAs and Pre-S/S RNAs (**g**) in L02 cells with INTS10 knockdown. (**h**,**i**) Levels of HBsAg (**h**) and HBeAg (**i**) in supernatants of L02 cells with INTS10 knockdown. (**j**) Protein levels of INTS10 in cellular lysates of HepG2.2.15 cells. HepG2.2.15 cells (∼2 × 10^5^) were transfected with INTS10 or control vectors (up), or with INTS10-specific or control siRNAs(down). (**k**,**l**) Levels of HBV DNAs (**k**), pgRNAs and Pre-S/S RNAs (**l**) in HepG2.2.15 cells with INTS10 overexpression. (**m**,**n**) Levels of HBsAg (**m**) and HBeAg (**n**) in supernatants of HepG2.2.15 cells with INTS10 overexpression. (**o**,**p**) Levels of HBV DNAs (**o**), pgRNAs and Pre-S/S RNAs (**p**) in HepG2.2.15 cells with INTS10 knockdown. (**q**,**r**) Levels of HBsAg (**q**) and HBeAg (**r**) in supernatants of HepG2.2.15 cells with INTS10 knockdown. All the supernatants and cells were collected 72 h post-transfection. Protein levels of INTS10 were examined by western blot analyses. HBV DNA levels in cells were measured by Southern blot analysis (left) and quantitative real-time PCR (qRT-PCR; right). The pgRNAs and Pre-S/S RNAs of HBV in cells were measured by northern blot analysis with 18S ribosome RNA (rRNA) indicating RNA loading in each lane (left), and qRT-PCR normalized to human β-actin gene *ACTB* (right). The levels of HBsAg and HBeAg in supernatants were measured by enzyme-linked immunosorbent assays (ELISA). Error bars indicate s.d. *P* values were determined using two-tailed unpaired *t*-test. *P<0.05, **P<0.01 and ***P<0.001. rcDNA, relaxed circular DNA; dsDNA, double-stranded DNA; ssDNA, single-stranded DNA.

To investigate whether the INTS10 plays a role on HBV replication, we used *in vitro* cell culture assay systems. The immortalized human hepatocyte cell line L02 was transfected with pAAV-HBV1.2 vectors, together with either pLV-EGFP-INTS10 or pLV-EGFP control vectors (Fig. 2a). Compared with the cells expressing control vectors, the significantly decreased levels of HBV markers, including replicative intermediates of HBV DNAs, HBV RNAs, HBsAg and hepatitis B e antigen (HBeAg), were observed in cells stably expressing exogenous INTS10 (all *P*<0.01; Fig. 2b–e and Supplementary Table 12). Consistent with these findings, knockdown by two independent siRNAs targeting *INTS10* led to significantly elevated levels of HBV DNAs, HBV RNAs, HBsAg and HBeAg (all *P*<0.05; Fig. 2a,f–i). The antiviral activity of INTS10 against HBV is not limited to the L02 cells. INTS10 can also efficiently reduce the HBV markers in human hepatoma cell line HepG2.2.15 which constitutively produces HBV (Fig. 2j–r), and in human hepatoma cell line HepG2 which was co-transfected with the pAAV-HBV1.2 vectors (Supplementary Fig. 8). Taken together, these results suggest that INST10 plays a role in suppressing HBV replication *in vitro*.

### INTS10 suppresses HBV replication via IRF3-dependent manner

We then sought to explore the underlying mechanisms by which INTS10 suppresses HBV replication by analysing mRNA expression profiles of liver tissues from 31 HBV carriers (Supplementary Note). Comparing samples with high *INTS10* expression to samples with low *INTS10* expression, we identified 402 differentially expressed genes (false discovery rate *Q* value<0.01 and fold change>1.2; Supplementary Data 3a) in determining biological pathways that are altered after *INTS10* dysregulation. Intriguingly, we observed significant enrichment and activation of the spliceosome (*P*_nominal_=1.5 × 10^−5^, ranks the first) and the retinoic acid-inducible gene-I-like receptor (RLR) signalling pathway (*P*_nominal_=1.8 × 10^−3^, ranks the second; Supplementary Data 3b) in samples with high *INTS10* expression. Given the important roles of integrator complex in spliceosome, the enrichment of term spliceosome may reflect the intrinsic physiologic function of INTS10. Notably, however, the RLR members such as retinoic acid-inducible gene-I (RIG-I) and melanoma differentiation-associated gene 5 (MDA5) have been shown to sense the HBV and activate innate immune signalling in hepatocytes to suppress virus replication^29^,^30^,^31^. To determine whether the RLR signalling pathway was regulated by INTS10 in other independent samples, we performed similar analyses in two data sets from the Gene Expression Omnibus (GEO) database (accession number GSE25097 and GSE22058), which contain 289 and 96 liver tissues, respectively (Supplementary Data 3c,d). Again, the term spliceosome ranked the first in both data sets (*P*_nominal_=1.0 × 10^−8^ and 2.6 × 10^−3^, respectively), and the RLR-related pathway ranked the third (*P*_nominal_=2.6 × 10^−2^) and ninth (*P*_nominal_=0.20), respectively (Supplementary Data 3b). Taken together, these results suggest that INST10 may be involved in inhibition of HBV replication through the RLR pathway.

**Figure 3:**
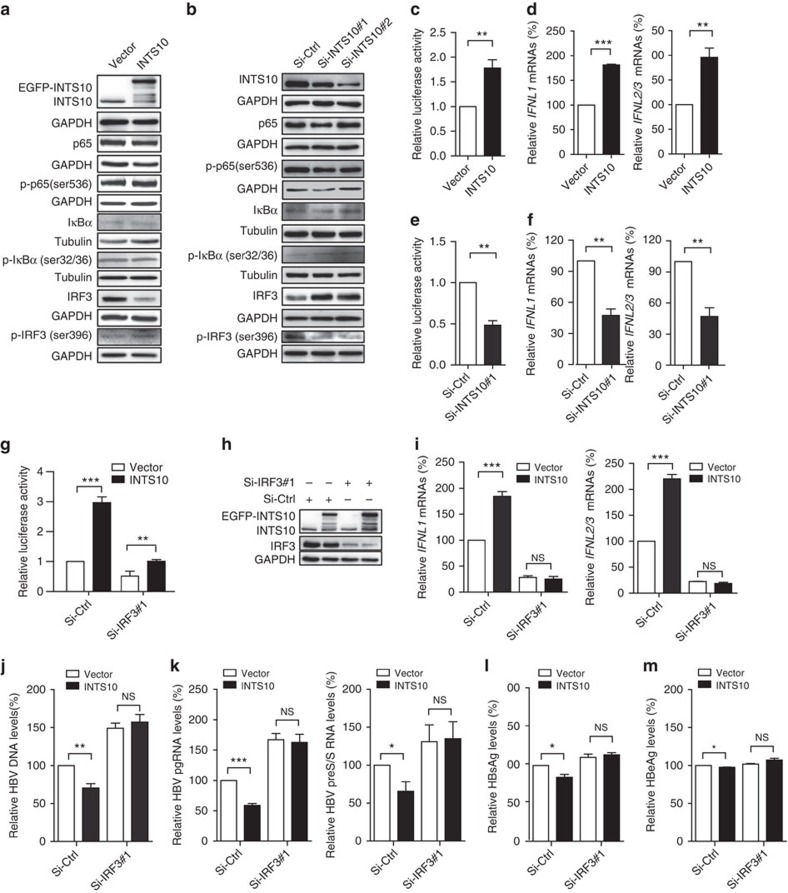
INTS10 suppresses HBV replication in an IRF3-dependent manner in HepG2.2.15 cells. (**a**,**b**) Levels of phosphorylated (p-) or total proteins in lysates of HepG2.2.15 cells measured by western blot analyses, with GAPDH or Tubulin indicating protein loading in each lane. The cells were transfected with pLV-EGFP-INTS10 vector (INTS10) or pLV-EGFP control vector (Vector) (**a**), or with INTS10-specific siRNAs (Si-INTS10#1 and Si-INTS10#2) or non-targeting scrambled siRNA controls (Si-Ctrl) (**b**). (**c**) Luciferase activity of ISRE reporter plasmids 48 h after co-transfection into cells with INTS10 or control vectors. RLU, relative luciferase units. (**d**) The mRNA levels of *IFNL1* and *IFNL2/3* measured by quantitative real-time PCR (qRT-PCR) normalized to human β-actin gene *ACTB* in cells transfected with INTS10 or control vectors. (**e**) Luciferase activity of ISRE reporter plasmids 48 h after co-transfection into cells with INTS10-specific or control siRNAs. (**f**) The mRNA levels of *IFNL1* and *IFNL2/3* measured by qRT-PCR normalized to *ACTB* in cells transfected INTS10-specific or control siRNAs. (**g**) Luciferase activity of ISRE reporter plasmids 48 h after co-transfection into cells with INTS10 or control vectors and an IRF3-specific siRNA (Si-IRF3#1) or non-targeting scrambled siRNA controls (Si-Ctrl). (**h**) Levels of INTS10 and IRF3 measured by western blot analyses, with GAPDH indicating protein loading in each lane. (**i**) The mRNA levels of *IFNL1* (left) and *IFNL2/3* (right) measured by qRT-PCR normalized to *ACTB* in cells co-transfected with INTS10 or control vectors and IRF3-specific or control siRNAs. (**j**,**k**) Levels of HBV DNAs (**j**), 3.5 Kb pregenomic RNAs (pgRNAs) and 2.4/2.1 Kb Pre-S/S RNAs (**k**) in cells co-transfected with INTS10 or control vectors andIRF3-specific or control siRNAs measured by qRT-PCR normalized to *ACTB*. (**l**,**m**) Levels of HBsAg (**l**) and HBeAg (**m**) in supernatants of cells co-transfected with INTS10 or control vectors and IRF3-specific or control siRNAs measured by enzyme-linked immunosorbent assays (ELISA). All histograms show mean values from three independent experiments; error bars indicate s.d. *P* values were determined using two-tailed unpaired *t*-test. *P<0.05, **P<0.01 and ***P<0.001.

**Figure 4:**
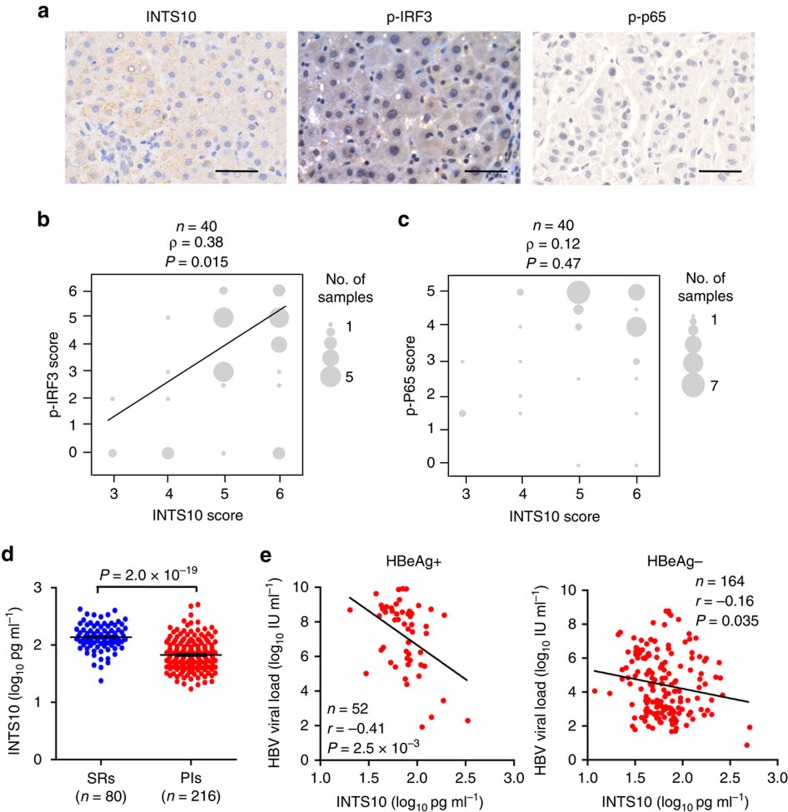
The levels of INTS10, p-IRF3, p-p65 and HBV DNA in clinical samples. (**a**) Representative images from the liver tissues by immunohistochemistry staining are shown for INTS10, p-p65 and p-IRF3, respectively. The scale bar represents 50 μm. (**b**,**c**) The correlation of protein levels between INTS10 and p-IRF3 (**b**), or between INTS10 and p-p65 (**c**). Protein levels of INTS10, p-p65 and p-IRF3 were measured in the non-tumour liver tissues of patients with HBV-related HCC by immunohistochemistry staining (*n*=40). The size of the circle is proportional to the number of samples. A Spearman's test was used, and the correlation coefficiency (*ρ*) and the two-tailed *P* values are shown. (**d**) The concentration of the plasma INTS10 in persistently HBV infected subjects (PIs) and spontaneously recovered subjects (SRs). The plasma INTS10 levels were measured by enzyme-linked immunosorbent assays (ELISA) in 216 PIs and 80 SRs. Horizontal bars indicate the mean value of each subset. The significance was calculated using two-tailed unpaired *t* test. (**e**) Correlation between the plasma INTS10 and HBV DNA load in PIs with positive HBeAg (left) and PIs with negative HBeAg (right). The plasma INTS10 and HBV DNA load were log10 transformed. The correlation coefficiency (*r*) and the two-tailed *P* values were then evaluated by Pearson's test. *P* values were considered to be significant when below 0.05.

Binding of RLRs to virus-derived nucleic acids activates the downstream signalling pathways in a manner dependent on the adaptor protein mitochondrial antiviral signalling protein (also known as IPS-1, VISA or Cardif), leading to the activation of the IRF3 and NF-κB and the subsequent production of type I interferons (IFNs, including IFN-α and IFN-β) and type III IFNs (that is, IFN-λ, including IFNL1 (also known as IL29), IFNL2 (IL28A) and IFNL3 (IL28B)) and inflammatory cytokines^32^. Thus, we examined whether the HepG2.2.15 cells transfected with the INTS10 expression plasmid could activate the IRF3 and NF-κB. We found that overexpression of INTS10 could increase IRF3 phosphorylation (p-IRF3), whereas the NF-κB could not be activated (Fig. 3a). Consistent with these findings, knockdown of INTS10 by siRNAs led to significantly decreased levels of p-IRF3, whereas not influencing the activity of NF-κB (Fig. 3b). Furthermore, we found that overexpression of INTS10 could potently activate IFN-stimulated response element (ISRE) in reporter assays (*P*<0.01; Fig. 3c) and elevate mRNA levels of type III IFNs (*IFNL1* and *IFNL2/3*; *P*<0.05; Fig. 3d and Supplementary Table 12), but not influence the type I IFNs. Consistent with these findings, knockdown of INTS10 significantly reduced the activity of ISRE reporter and mRNA levels of *IFNLN1* and *IFNLN2/3* (Fig. 3e,f). To ensure that these observations could be applied to other types of hepatocytes, we then did the same experiments in L02 and HepG2 cells co-transfected with HBV1.2 vectors and obtained identical results (Supplementary Figs 9 and 10). Next, we investigated whether the IRF3 pathway is required for the INTS10-elicited immunity against HBV infection. Indeed, we found that the activation of ISRE reporter, elevation of mRNA levels of type III IFNs and the reduction of HBV markers by enforced INTS10 expression were weakened when cells were transfected with siRNAs targeting IRF3 (Fig. 3g–m and Supplementary Fig. 11). Accordingly, in the liver tissues of patients persistently infected with HBV, we observed that the protein levels of INTS10 were positively correlated with those of p-IRF3 (ρ=0.38, *P*=0.015), but not p-p65 (Fig. 4a–c and Supplementary Table 13). Taken together, these results suggest that INTS10 suppresses HBV replication in an IRF3-dependent manner.

### INTS10 correlates with the persistence of HBV infection

To further validate the roles of INTS10 in facilitating HBV clearance, we investigated the plasma INTS10 from subjects persistently infected HBV or those spontaneously recovered from HBV infection. Consistent with the eQTL result in liver tissues, we observed elevated INTS10 protein levels in the plasma of rs7000921 C allele carriers (*P*=0.020, unpaired *t*-test; Supplementary Fig. 12). Furthermore, the levels of plasma INTS10 in 216 PIs were significantly lower than those in 80 SRs (*P*=2.0 × 10^−19^, fold change=2.0; Fig. 4d and Supplementary Table 1b). In addition, we found significantly negative correlation between the INTS10 levels and HBV DNA load in the plasma of PIs with positive HBeAg (Pearson correlation coefficient *r*=−0.41, *P*=2.5 × 10^−3^) and those with negative HBeAg (*r*=−0.17, *P*=0.028; Fig. 4e). Taken together, these results further support our genetic and functional findings, indicating that insufficiency of INTS10 may contribute to the persistence of HBV infection.

## Discussion

To date, hundreds of GWASs have been performed to investigate the genetic susceptibilities to common diseases, but very few of them have been re-used since their initial publication. In the present study, by using previously reported GWAS data among Chinese and re-examining phenotypes related to the HBV infection, we obtained a relatively large case–control population (totally including 1,251 PIs (cases) and 1,057 SRs (controls)) to perform a GWAS for the persistence of HBV infection. Thus, enough statistical power, >90% at significance level of 0.01, was provided to detect an allele with a minor allele frequency (MAF) of 0.20 that confers an additive 1.2-fold effect on disease risk (Supplementary Fig. 13). Through replication studies among four independent populations (totally consisting of 3,905 PIs and 3,356 SRs), we identified a novel association signal at 8p21.3 (index SNP rs7000921). Our study demonstrates that novel insights into disease biology can be obtained by re-using previously published GWAS data sets.

In a separate study, we genotyped the rs7000921 in 689 Chinese subjects without information on HBV infection status (Supplementary Table 1c). The frequencies of the rs7000921[C] allele in the SRs (0.283) were similar to that in this random control set (0.266; Supplementary Table 14). To gain insight into the geographic frequency distribution of rs7000921, we compared the SRs with the 14 populations from the 1000 Genomes Project. The frequency of the rs7000921[C] allele in the SRs was similar to that of Asians (0.210–0.309, *P*=0.20), but significantly higher than that of Europeans (0.107–0.218, *P*=7.8 × 10^−11^) and significantly less than that of Africans (0.484–0.691, *P*=1.4 × 10^−51^;Supplementary Fig. 14 and Supplementary Table 14). It remains to be determined whether these differences between ethnic groups influence susceptibility to the persistence of HBV infection.

The rs7000921 lies in a noncoding region at 8p21.3. No coding SNPs show high LD with the rs7000921 (Supplementary Table 11). As noncoding SNPs may alter gene expression, we used eQTL analyses to explore whether the rs7000921 or SNPs tagged by it are *cis*-acting regulators of nearby gene(s) in human liver. Indeed, we found that the eQTL associations with *INTS10* were statistically significant in two independent sample sets of liver tissues, strongly suggesting a causative role of altered *INTS10* expression on phenotypes associated with rs7000921. However, it is possible that eQTLs exist for risk SNPs with genes other than *INTS10*, either within this region or regulated more distally. Additionally, eQTL analyses are complicated by tissue heterogeneity due to variation in genomic copy number, methylation and gene expression. Therefore, caution needs to be applied when interpreting eQTL data. Additional analyses in larger sample sizes and in more cell or tissue types relevant to aetiology of persistent HBV infection will be needed to confirm the significance of INTS10 as susceptibility gene for persistence of HBV infection at 8p21.3.

INTS10 is biologically plausible for susceptibility to persistent HBV infection. Although the specific roles of INTS10 in persistent HBV infection have never been reported before, functional assays in this study indicated that INTS10 can significantly decrease levels of HBV markers in liver cells (Fig. 2 and Supplementary Fig. 8). Consistent with the roles of INTS10 in suppressing HBV replication, the levels of plasma INTS10 in PIs were significantly lower than those in SRs (Fig. 4d), and the INTS10 levels were significantly negatively correlated with the HBV DNA levels in the plasma of PIs (Fig. 4e). Taken together, these findings suggest that insufficiency of INTS10 is biologically plausible for an increased risk of HBV infection.

Previous studies have revealed the roles of RLR pathway in control of HBV infection^29^,^30^,^31^,^33^,^34^,^35^,^36^,^37^. For instance, the RNA sensor RIG-I has been reported to dually function as an innate sensor of the 5′-ɛ region of HBV pregenomic RNAs to induce type III IFNs but not type I IFNs, and as a direct antiviral factor to counteract the interaction of HBV polymerase with the 5′-ɛ region of HBV, which consistently suppressed HBV replication^29^. Meanwhile, the HBV X protein can target RLR pathway to suppress virus-triggered IRF3 activation and IFN-β induction^33^,^34^,^35^,^36^. Consistent with these previous studies, our study for the first time demonstrated that the INTS10 promotes HBV clearance through activation of IRF3 in liver cell models. Further studies are warranted to identify the interaction protein(s), and elucidate the precise mechanisms of INTS10 against HBV infection in hepatocytes, and/or other types of cell including dendritic cell subsets.

In summary, our GWAS replicates the previously identified 6p21.32, 6p21.33 and 20q13.12, and reveals a novel locus at 8p21.3 contributing to the susceptibility to persistent HBV infection. We for the first time identify the *INTS10* gene at 8p21.3 as causative for HBV clearance by activation of IRF3 and then expression of anti-virus IFNs. Our work indicates that the INTS10 insufficiency contributes to the viral persistence; it may shed light on the prevention and clinical implications of INTS10 in control of HBV infection.

## Methods

### Study samples

For detailed descriptions of all case–control sample sets, see Supplementary Note. All the subjects above were unrelated ethnic adult Chinese. This study was performed with the approval of the Medical Ethical Committee of Beijing Institute of Radiation Medicine (Beijing City, China). Written informed consent was obtained from each participant, and personal information on demographic factors was collected by structured questionnaire. The investigators were blind to the case/control status of subjects during all genotyping experiments.

### Case–control populations

Subjects who had been positive for both HBsAg and anti-HBc immunoglobulin G for at least 6 months were defined as PIs (cases). Those who were negative for HBsAg and positive for both anti-HBs and anti-HBc immunoglobulin G were defined as SRs (controls). In the discovery GWAS stage, the genotype data were derived from several previously published GWASs and in-house data^12^,^13^,^14^,^15^. By screening for HBV markers in the plasma of these subjects whose plasma samples were available, we determined 1,251 cases and 1,057 controls (Supplementary Table 1a). With the same sample inclusion and exclusion criteria as those used in the discovery GWAS stage, we totally determined 3,905 cases and 3,356 controls in the replication stage (Supplementary Table 1a) (refs 16, 17).

### Random controls without information on HBV infection

This population consists of 689 subjects, which was used to evaluate the frequency of the protective rs7000921 allele [C] in naïve controls in China. All the subjects were unrelated ethnic adult Chinese randomly recruited from Guangxi province, and were not screened for HBV markers in previous studies. The male/female ratio and the mean age (s.d.) of these random controls are 1.4 (403/286) and 54.9 (11.8) years old, respectively (Supplementary Table 1b).

Protein levels of INTS10, p-p65 and p-IRF3 were measured by immunohistochemistry (IHC) in 40 non-tumour liver tissues of patients with HBV-related HCC (Supplementary Table 13) collected from the Jinling Hospital (Nanjing City, China). The levels of plasma INTS10 were detected by enzyme-linked immunosorbent assays in 216 PIs and 80 SRs, who were randomly selected from the Guangdong population in the replication stage (Supplementary Table 1c).

### Quality controls in the GWAS stage

We performed stringent quality controls on both samples and SNPs to ensure subsequent robust association tests. Samples were removed if they (i) had an overall genotyping rate of<90%; (ii) showed sex discrepancies; (iii) showed unexpected duplicates or relatives (PI_HAT>0.025) or (iv) were identified as outliers. SNPs were excluded if they had (i) a call rate of<90%; (ii) a MAF of <0.05; or (iii) a *P* value of <1 × 10^−4^ in a Hardy–Weinberg equilibrium test among controls. After quality controls, a total of 1,251 cases and 1,057 controls were remained; and, 616,583, 286,713, 694,784, 590,809 and 441,776 SNPs, respectively, were remained for GWAS population 1, 2, 3, 4 and 5, respectively, for subsequent analyses (Supplementary Table 2).

### SNP imputation

To increase the number of overlapping SNPs among data sets and generate more genotypes in the discovery GWAS stage, we performed imputation on the GWAS data sets using a Markov Chain based haplotyper (MACH; version 1.0.16)^38^ with haplotypes derived from genotypes of samples of Asian ancestry in the HapMap phase II, the HapMap phase III and the 1000 Genomes Project. Before imputation, all the SNPs were checked for strand inconsistencies. Then, data were imputed by a two-stage design for all the data sets. The first stage generated error and crossover maps as parameter estimates, which were used to generate maximum likelihood estimates of allele numbers per SNP on the basis of reference haplotypes for the data sets during the second stage of the imputation. The imputation were performed for each data set separately. Cases and controls within each data set were imputed together. For detailed descriptions, see Supplementary Note.

### Assessment of accuracy of array genotyping and imputation

To repeat the array genotyping and imputation results, we randomly selected 274 samples from the GWAS population 2, and then resequenced a ∼127-Kb non-repeat genomic region at 1p36.22 locus in them using deep sequencing. BWA (v0.5.9) was used to aligned high quality reads to human genome (hg19 build) with default parameters. SAMtools (v0.1.8) was used to remove PCR duplicates. When accounting for the array genotyping accuracy, Kappa test was used to evaluate the agreement between the genotypes determined by array genotyping and those determined by deep sequencing. When accounting for the imputation accuracy, we calculated the Pearson's correlation coefficients between the ‘allele dosages' from the imputed data based on the 1000 Genomes Project and the non-reference allele proportions of SNPs in the deep sequencing. For detailed descriptions, see Supplementary Note.

### Genome-wide genetic association analyses in the GWAS stage

We combined all five GWAS subgroups to perform joint association analyses. Population substructure was characterized using principal component analyses as implemented in EIGENSTRAT (version 3.0) (ref. 39). We did genome-wide association analyses at every SNP using MACH2DAT (ref. 40) by use of imputation results based on HapMap phase II, HapMap phase III and 1000 Genomes Project data, respectively. To account for imputation uncertainty, we used ‘allele dosages' as a primary predictor of persistent HBV infection in logistic regression models adjusted for age, sex and admixture principal components. Haploview (v4.2) and R package were used to generate Manhattan plot of -log_10_ (*P*) and quantile–quantile plot, respectively. We also performed meta-analysis in the GWAS stage. For detailed descriptions, see Supplementary Note.

### Text mining of candidate gene-based association studies

We searched the PubMed database (http://www.ncbi.nlm.nih.gov/sites/pubmed) for papers published before 15 Mar 2011, according to the following keywords: (polymorphism or polymorphisms) and (single nucleotide polymorphism or SNP) and (hepatitis B virus or HBV). Thus, the papers that tested the genetic associations between SNPs and chronic/persistent HBV infection were included. The reviews and the papers performing investigations for other liver diseases were excluded. The studies lacking statistical analyses were also excluded. Finally, a total of 76 papers were remained. From these papers, the SNPs that were reported to be significantly associated with the chronic or persistent HBV infection were selected. Then the SNPs with MAF<0.01 in East Asian population according to the 1000 Genomes Project were removed. Finally, a total of 69 SNPs were remained for reviewing the consistency of associations in this study (Supplementary Table 6).

### Text mining of GWASs on HBV-related phenotypes

We searched the studies based on GWAS Catalog (http://www.genome.gov/26525384). Eight studies on HBV-related phenotypes were selected. We reviewed these papers and selected 25 significantly associated SNPs for reviewing the consistency of associations in this study (Supplementary Table 8).

### HLA alleles analyses

HLA alleles were predicted from dense SNPs genotypes using the R package HIBAG (http://cran.r-project.org/web/packages/HIBAG/index.html). We selected a threshold of 0.5 as a value that has modest effects on both call rate and accuracy. ORs and 95% confidence intervals were calculated in logistic regression model.

HLA-DRB1*1301 and*1302 alleles (corresponding to *HLA-DR13*) were reported to have a protective effect against persistent HBV infection in different populations^41^,^42^. However, *HLA-DR13* were failed to be predicted with the R package HIBAG in this study. We then selected rs11752643 as a proxy of *HLA-DR13* (rs11752643 is closely linked with *HLA-DR13,* with *r*^*2*^=0.83 in healthy Japanese samples)^6^. We found that rs11752643 showed marginal association with persistent chronic HBV infection (*P*=0.089).

### Selection of SNPs for the replication studies

In the joint analyses, a locus not reported previously to conferring susceptibility to persistent HBV infection was chosen for replication if it had a SNP with a *P* value ≤1.0 × 10^−4^ in the GWAS stage. SNPs showing independent association (*P*≤0.05) by conditional analyses with adjustment of the most significant SNP within each region went forward to replication stage (Supplementary Note). These steps led to the identification of 72 candidate SNPs forward to the replication stage. In the meta-analysis, the same steps were performed. This led to the identification of 43 candidate SNPs, all of which were overlapped with the 72 most significant SNPs in joint analyses.

### Genotyping and quality controls in the replication stage

In the replication stage 1, one SNP within MHC region was genotyped using TaqMan assays, and the remaining 71 SNPs were genotyped using Sequenom assays with five failed. Among the 67 successfully genotyped SNPs, six SNPs survived (*P*<0.05 and with effects in the same direction as in the GWAS stage) and went forward to the replication stage 2. In this stage, four SNPs were genotyped using Sequenom assays and two SNPs using TaqMan assays. The SNP rs7000921 survived in the replication stage 2 and then went forward to the subsequent replication stages 3 and 4 using TaqMan assays.

In the Sequenom assays, the sample DNAs were amplified by multiplex PCR, then the products were used for locus-specific single-base extension reaction and detected for alleles using matrix-assisted laser desorption/ionization time-of-flight mass spectrometry (Sequenom). TaqMan assays were performed according to the manufacturers' instructions (Applied Biosystems).

In each of the replication stage, the genotype data were subjected to the same quality control analyses as in the GWAS stage. The cluster patterns of the genotyping data from the Sequenom and TaqMan assays were visually checked to confirm their good quality. The associations were carried out with additive model using PLINK (v1.07).

Meta-analysis of data generated from both GWAS and replication stages was conducted to assess the pooled genetic effects using meta-analysis helper (METAL) software^43^. Cochran's *Q* statistic were calculated to test between-group heterogeneity.

The potential modification effects of sex and age on the association between rs7000921 and persistent HBV infection risk were assessed both by adding interaction terms in the logistic regression model and by separate analyses of subgroups of subjects stratified by these factors.

For detailed descriptions, see Supplementary Note.

### Statistical analyses

Fisher's exact test or *χ*^2^ test was used for the analyses of contingency tables depending on the sample sizes. *P* values were calculated by two-sided Student's *t*-test for means of age, activities of reporter genes, expression levels of HBV markers and mRNA expression levels of IFNs. The estimate of variation within each group of data was carried out by F-test. If measured values did not meet the assumptions of normality and homogeneity of variances, log-transformation was used before *t*-tests were performed. *P*<0.05 was considered as statistical significance. *P* values for the correlation between the genotypes of rs7000921, rs11991803 and rs4922214, and the mRNA levels of indicated genes in liver tissues (log2 transformed) were determined using the linear regression analyses adjusting for sex and age; those were considered to be significant when below 0.05 after Bonferroni correction by multiplying with the number of comparisons. Spearman's test was used to evaluate the correlation coefficiency (*ρ*) and the two-tailed *P* values of the expression levels of INTS10, p-p65 and p-IRF3, which were non-normally distributed variables. Pearson's test was used to evaluate the correlation coefficiency (*r*) and the two-tailed *P* values of the plasma INTS10 and HBV DNA load. Log-transformation was used before Pearson's test. *P*<0.05 was considered as statistical significance.

For detailed descriptions of genotype-expression analyses, functional annotations, pathway enrichment analyses and functional assays (including cell transfections, western blotting assays, detection of HBV DNAs and RNAs, quantitative real-time PCR assays, enzyme-linked immunosorbent assays, luciferase reporter gene assays and immunohistochemistry assays), see Supplementary Note.

### Data availability

The mRNA expression data that support the eQTL findings of this study have been deposited in GEO with accession code GSE74925. We confirmed that all relevant data are available from us.

## Additional information

**Accession codes:** The mRNA expression profiles of tissues from the 31 subjects have been deposited in GEO with accession code GSE74925.

**How to cite this article:** Li, Y. *et al*. Genome-wide association study identifies 8p21.3 associated with persistent hepatitis B virus infection among Chinese. *Nat. Commun.* 7:11664 doi: 10.1038/ncomms11664 (2016).

## Supplementary Material

Supplementary Information Supplementary Figures 1-15, Supplementary Tables 1-14, Supplementary Note 1 and Supplementary References

Supplementary Data 1 Summary of the GWAS and replication studies of top 72 SNPs.

Supplementary Data 2 rs7000921 genotypes and INTS10 mRNA expression levels in liver samples.

Supplementary Data 3 Differentially expressed genes in determining biological pathways that are altered after INTS10 dysregulation in liver tissues.

## Figures and Tables

**Table 1:** Summary of the data or samples used in the GWAS and replication stage.

	**Cases**			**Controls**		
	**Sample size**	**Mean age, yr (s.d.)**	**Females /males**	**Sample size**	**Mean age, yr (s.d.)**	**Females /males**
*GWAS stage*						
GWAS population 1	286	37.2 (10.1)	0/286	656	37.1 (10.8)	0/656
GWAS population 2	707	43.7 (11.6)	94/613	0	—	—
GWAS population 3	78	62.8 (8.3)	58/20	74	66.0 (7.2)	41/33
GWAS population 4	91	56.6 (10.0)	17/74	203	57.5 (9.7)	61/142
GWAS population 5	89	46.3 (11.1)	23/66	124	47.7 (13.0)	33/91
Overall	1,251	44.4 (12.7)	192/1,059	1,057	44.2 (14.6)	135/922
						
*Replication stage*						
Replication 1	1,279	50.5 (11.1)	524/755	1,360	50.4 (11.0)	567/793
Replication 2	1,299	38.2 (10.7)	493/806	1,067	38.4 (12.4)	416/651
Replication 3	783	39.8 (14.9)	324/459	560	40.7 (17.0)	252/308
Replication 4	544	35.5 (11.9)	129/415	369	28.0 (10.5)	77/292
Overall	3,905	42.2 (13.3)	1,470/2,435	3,356	42.5 (14.5)	1,312/2,044

Cases, persistently HBV infected subjects (PIs); controls, spontaneously recovered subjects (SRs); yr, year; –, not applicable.

The data in the GWAS stage were derived from previously published GWASs and in-house data^11^,^12^,^13^,^14^. GWAS populations 1, 2 and 3 were from Guangxi province. GWAS population 4 was from Jiangsu province. GWAS population 5 was from Guangdong province. For GWAS populations 1, 2, 3, 4 and 5, the original sample sizes were 1,999, 707, 436, 5,408 and 3,477, respectively; and genotyping arrays were Illumina Omini one, Affymatrix SNP 5.0, Illumina Omni Zhonghua, Affymatrix SNP 6.0 and Illumina Human610-Quad, respectively. In the replication stage, replication 1 samples are from Jiangsu province^15^. Replication 2 samples are from Guangxi province. Replication 3 samples are from Guangdong province. Replication 4 samples are from the Beijing City^16^. The details for the data of GWAS populations and samples in the replication stage were described in Methods section.

**Table 2:** Association results for the rs7000921 at 8p21.3 in five case–control populations.

**Studies**	Cases*	Controls*	**ORs (95% CI)**	***P*** **values**
GWAS	60/464/727	83/433/540	0.66 (0.56–0.79)	2.4 × 10^−6^
Replication 1	72/481/724	105/543/708	0.84 (0.74–0.95)	6.7 × 10^−3^
Replication 2	62/459/752	83/423/555	0.77 (0.68–0.88)	1.4 × 10^−4^
Replication 3	48/314/376	39/255/218	0.78 (0.65–0.94)	9.1 × 10^−3^
Replication 4	25/185/303	25/140/196	0.80 (0.64–1.02)	6.8 × 10^−2^
Overall	267/1,903/2,882	335/1,794/2,217	0.78 (0.73–0.84)	3.2 × 10^−12^

CI, confidence interval; cases, persistently HBV infected subjects (PIs); controls, spontaneously recovered subjects (SRs).

The GWAS population contains three independent case–control sub-populations of Southern Chinese ancestry, namely the Guangxi-GWAS population, the Jiangsu-GWAS population and the Guangdong-GWAS population (see Supplementary Note). Replication 1, 2, 3 and 4 samples are from Jiangsu, Jiangxi, Guangdong and Beijing, respectively. In GWAS population, ORs and 95% CIs were calculated under additive model by logistic regression while adjusting for age, sex and two admixture principal components. In the replication stage, ORs and 95% CIs were calculated under additive model by logistic regression while adjusting for age and sex. In overall samples, the meta-analysis gave a joint *P* value and a joint OR, with the *P* value for heterogeneity 0.29.

^*^Counts of minor allele homozygote (*CC*)/heterozygote (*CT*)/major allele homozygote (*TT*) genotypes in the cases and controls, respectively. The number of genotyped samples varies due to genotyping failure.
