# Supplementary material for: Genome-wide association study identifies 8p21.3 associated with persistent hepatitis B virus infection among Chinese
Source: Nat Commun. 2016 May 31;7:11664. doi: 10.1038/ncomms11664 (PMC4895015; doi:10.1038/ncomms11664)
Supplement: Supplementary Information — Supplementary Figures 1-15, Supplementary Tables 1-14, Supplementary Note 1 and Supplementary References [file ncomms11664-s1.pdf]

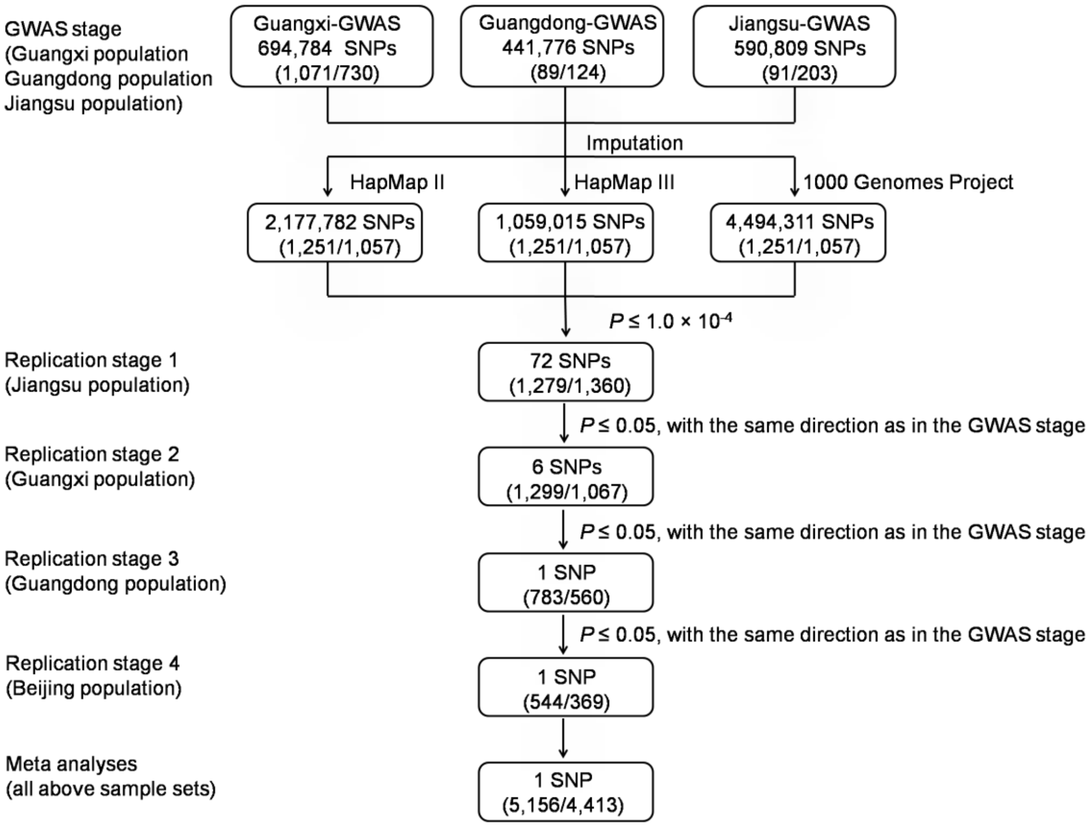

Supplementary Figure 1: An overview of the study workflow. Numbers refer to the cases and controls and the SNPs genotyped or imputed. Three rounds of imputation were performed using data from the HapMap project phase II, the HapMap project phase III and the 1000 Genomes Project as references and generated genotypes of 2,177,782, 1,059,015 and 4,494,311 SNPs, respectively. The 72 top significantly associated SNPs in the GWAS stage were genotyped in the samples of replication stage 1. Then, the confirmed SNPs ($P \leq 0.05$, and with the same directions as in the GWAS stage) were further genotyped in the samples of replication stage 2, 3 and 4. Lastly, a meta-analysis combining all the case-control studies for rs7000921 at 8p21.3 was performed.

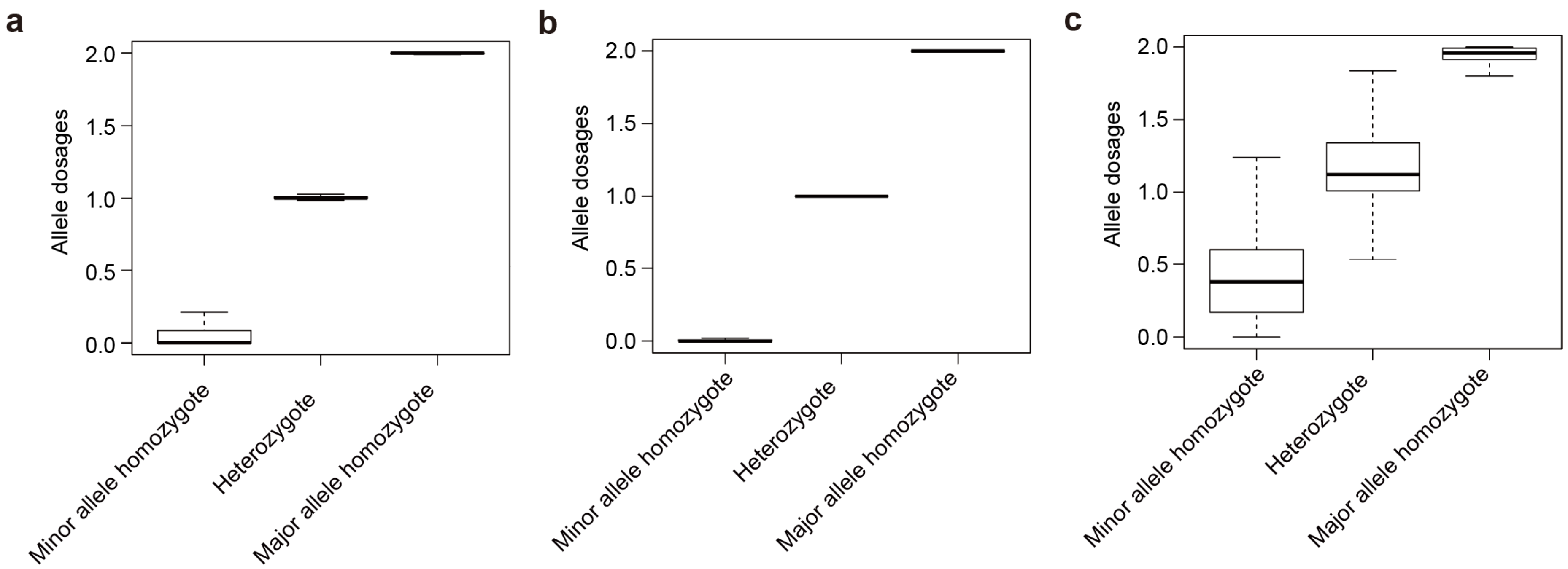

Supplementary Figure 2: The consistency of SNPs genotypes defined by imputation or sequencing. We evaluate the relationship between the "allele dosages" of SNPs determined by imputation and the genotypes of these SNPs detected by sequencing (including Agilent array capture based deep sequencing of 89 samples and long-range PCR based deep sequencing of 185 samples). (**a**) The consistency between the imputation and sequencing for all imputed SNPs (Pearson's correlation $r = 0.94$, $P = 1.8 \times 10^{-20}$). (**b**) The consistency between the imputation and

sequencing for SNPs with high imputation quality (imputation $r^2 > 0.8$; Pearson's correlation $r = 0.95$). (**c**) The consistency between the imputation and sequencing for SNPs with low imputation quality (imputation $r^2 \leq 0.8$; Pearson's correlation $r = 0.86$). Outliers are not shown.

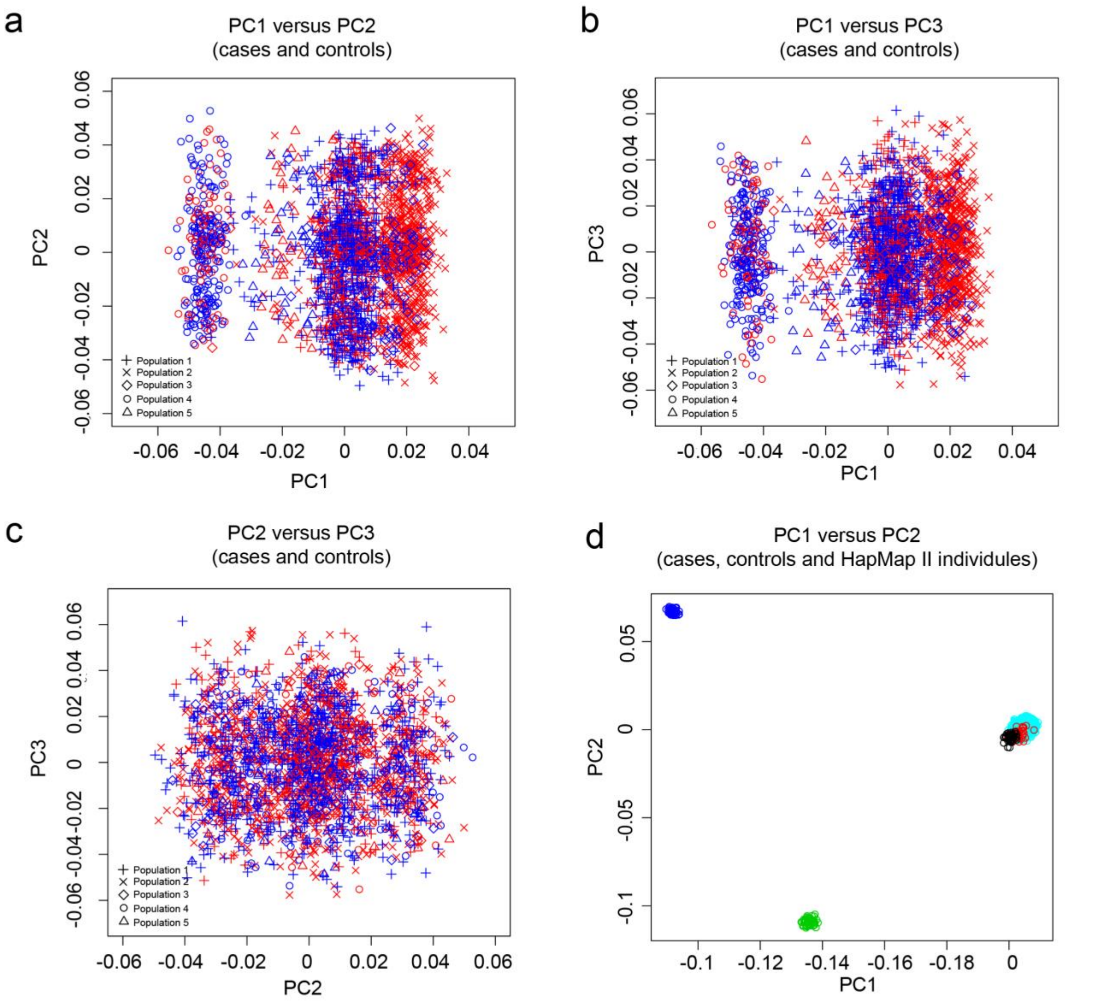

Supplementary Figure 3: The principal components analyses (PCA) of samples in the GWAS stage and reference samples from the HapMap project phase II. (**a**) Principal component (PC) 1 versus PC2 for the cases (n = 1,251) and controls (n = 1,057) in the GWAS stage. Red represents the cases and blue represents the controls. (**b**) PC1 versus PC3 for the cases and controls in the GWAS stage. Red represents the cases and blue represents the controls. (**c**) PC2 versus PC3 for the cases and controls in the GWAS stage. Red represents the cases and blue represents the controls. (**d**) PC1 versus PC2 for the cases and controls in the GWAS stage (light blue), and for the reference individuals in the HapMap II (n = 206), including 57 YRIs (Yoruba in Ibadan, Nigeria; dark blue), 60 CEUs (Utah residents (CEPH) with Northern and

Western European ancestry; green), 44 JPTs (Japanese in Toyko, Japan; black) and 45 CHBs (Han Chinese in Beijing, China; red). GWAS population 1, 2 and 3 were from Guangxi province. GWAS population 4 was from Jiangsu province. GWAS population 5 was from Guangdong province.

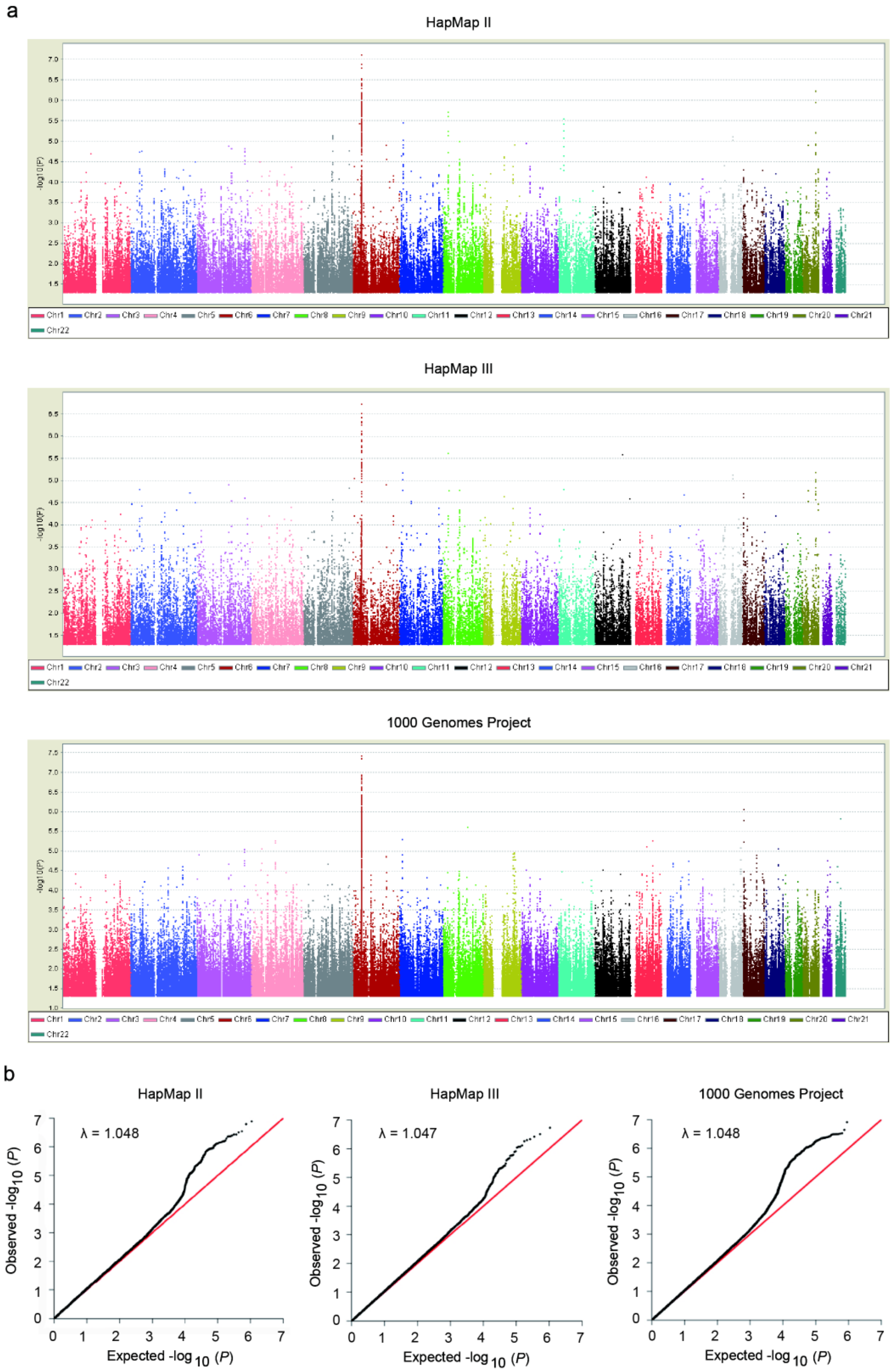

Supplementary Figure 4: Manhattan plots and Quantile-Quantile plots of *P*

values from association tests in the GWAS stage. (**a**) The Manhattan plot shows the associations for the genotyped and imputed SNPs with the persistent HBV infection. The x-axis represents the genomic positions (NCBI Build 36), and the y-axis shows $-\log_{10}(P)$. Within each chromosome shown on the x-axis, the data are plotted from the p-ter end. (**b**) The quantile-quantile plot. The red line represents the null hypothesis of no true association. The black line with gradient λ (inflation coefficiency) is fitted to the lower 90% of the distribution of the observed test statistics. The plot is based on the genotyped and imputed SNPs passed the quality controls. The values of inflation factor λ are 1.048, 1.047 and 1.048 using the data from the HapMap project phase II, HapMap phase III and 1000 Genomes Project as reference, respectively.

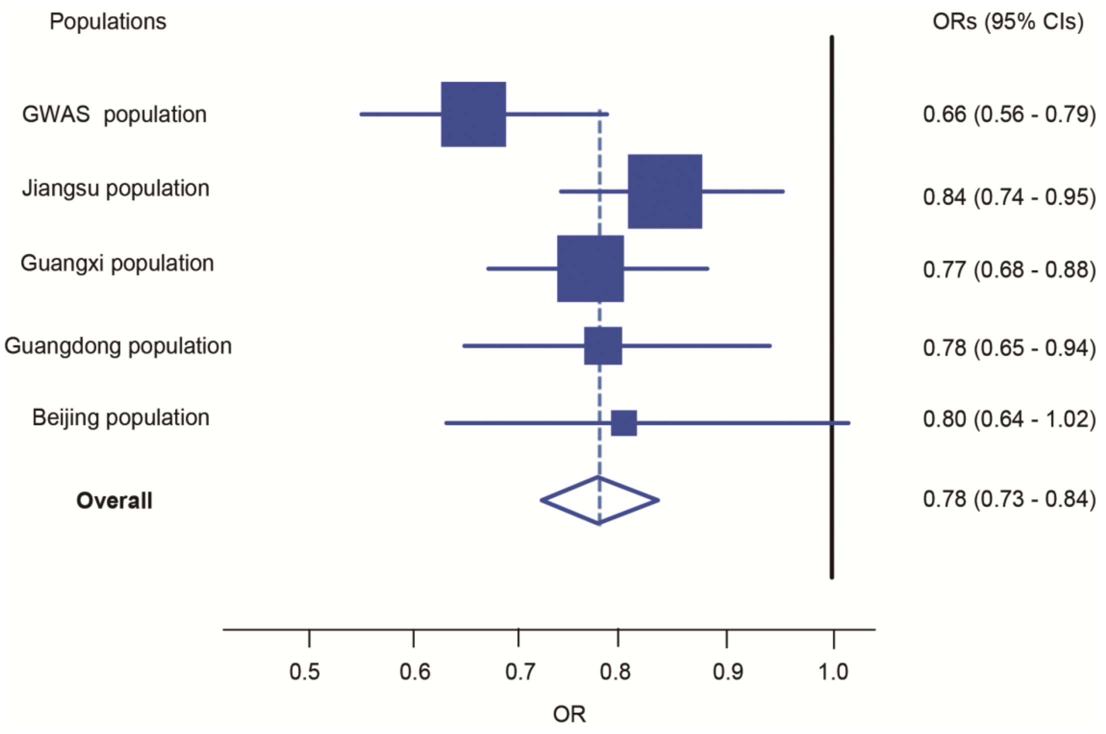

Supplementary Figure 5: Forest plot for the rs7000921 across all the case-control studies. We plot the odds ratio (OR; blue square) and the 95% confidence interval (CI; horizontal blue line) for each study. A vertical blue dashed line indicates the final ORs of rs7000921 across all five studies. The five bars represent results from five studies and the blue diamond below them summarizes their meta-analyzed effect. The area of each square is proportional to the weight of each study in the meta-analysis. Overall, the meta-analysis gave a joint P value of 3.2×10^{-12} (joint OR = 0.78, 95% CI = 0.73-0.84) for rs7000921. $P_{heterogeneity} = 0.29$.

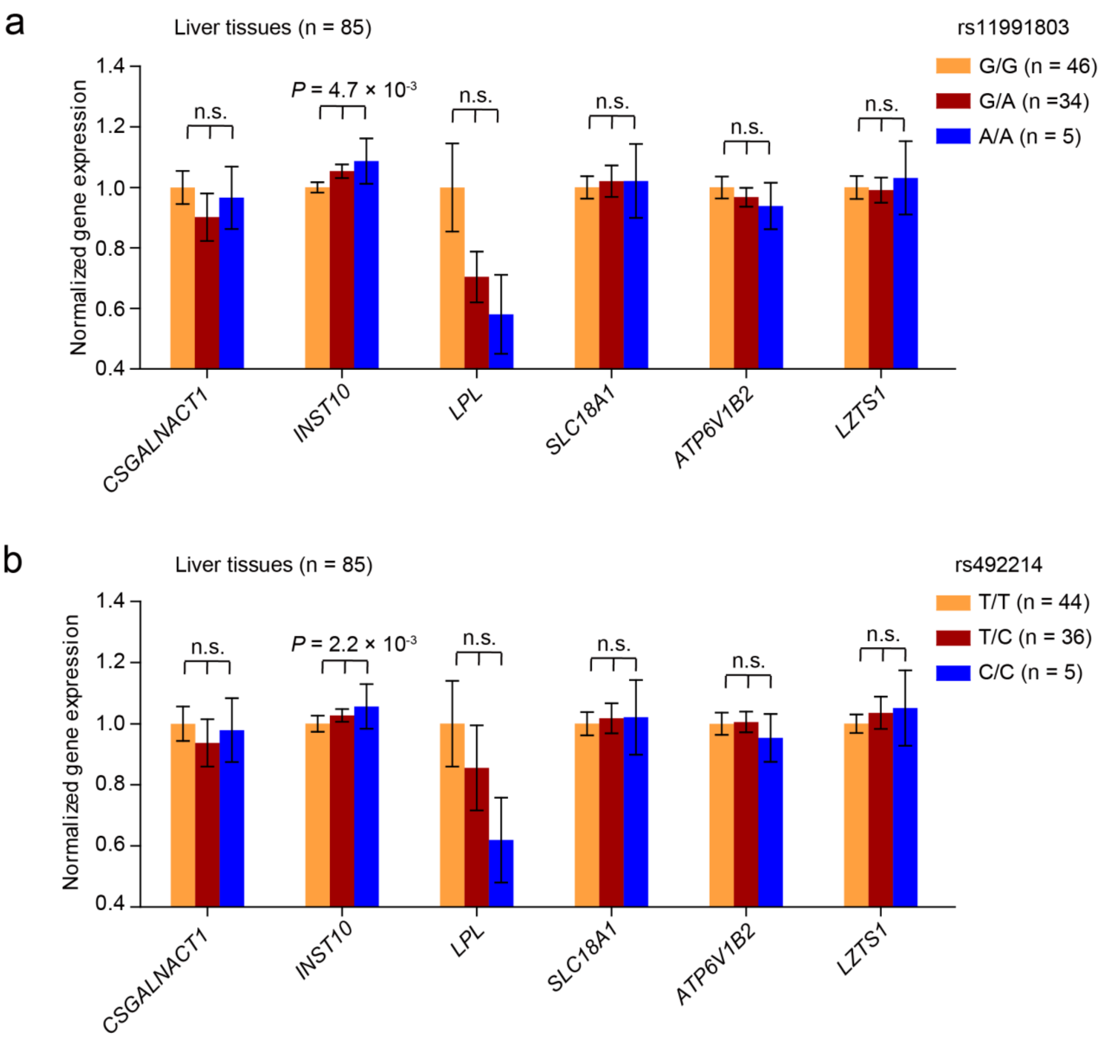

Supplementary Figure 6: The rs11991803 and rs4922214 at 8p21.3 locus are significantly associated with mRNA expression levels of *INTS10* gene. The genotypes of rs11991803 and rs4922214 were not available for the sample set of 31 HBV carriers which was used for the expression quantitative trait locus (eQTL) analyses. However, in the sample set of 88 HBV carriers, the genotypes of rs11991803 and rs4922214 have been determined by whole genome sequencing (European Genome-phenome Archive database, accession number ERP001196). The mRNA expression levels of liver tissues have also been measured by expression arrays (GEO datasets, accession number GSE25097). The intensity of all gene array experiments were normalized together using the Robust Multiarray Averaging (RMA)

method. Among the 88 liver samples, three subjects were considered as outliers (their mRNA expression levels of *INTS10* > mean + 3 s.d. or < mean - 3 s.d.), thus we only analyzed the remaining 85 individuals. (**a**) Expression levels of nearby genes in 85 human liver tissue samples with different genotypes of rs11991803 (GG, GA and AA). (**b**) Expression levels of nearby genes in 85 human liver tissue samples with different genotypes of rs4922214 (TT, TC and CC). Expression levels of each gene were normalized to the mean level of homozygotes for the major allele of rs11991803 (GG genotype, **a**) or rs4922214 (TT genotype**, b**). *P* values were derived from linear regression analyses, and were considered to be significant when below 0.05 after Bonferroni correction by multiplying with the number of comparisons. Error bars indicate standard error of the mean (s.e.m.). n.s., not significant. s.d., standard deviation.

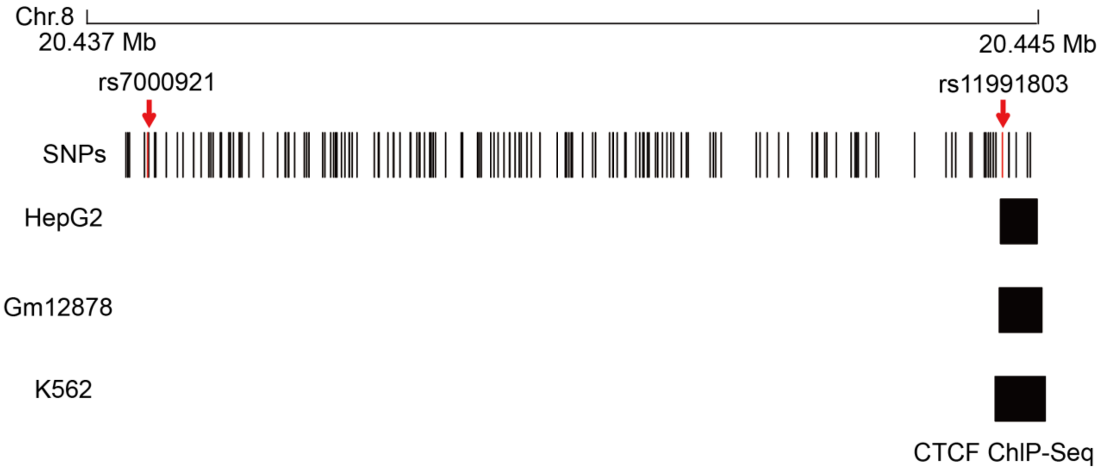

Supplementary Figure 7: The CTCF ChIP-Seq data for the candidate causative SNP rs11991803. All the SNPs from Chr.8:20437300-20445000 (positions were based on NCBI Build 36) are shown; the index SNP rs7000921 and the candidate causative SNP rs11991803 were indicated by red arrows. We used Encyclopedia of DNA Elements (ENCODE) chromatin immunoprecipitation and sequencing (ChIP-Seq) data for the transcriptional repressor CCCTC-binding factor (CTCF) at 8p21.3. The rs11991803 was within a CTCF-binding site which was detected by HaploReg (http://www.broadinstitute.org/mammals/haploreg/haploreg.php) in multiple cell types including the human hepatoma cell line HepG2, human normal B-lymphocyte cell line Gm12878 and human immortalized myelogenous leukemia cell line K562. Chr., chromosome. SNP, single-nucleotide polymorphism.

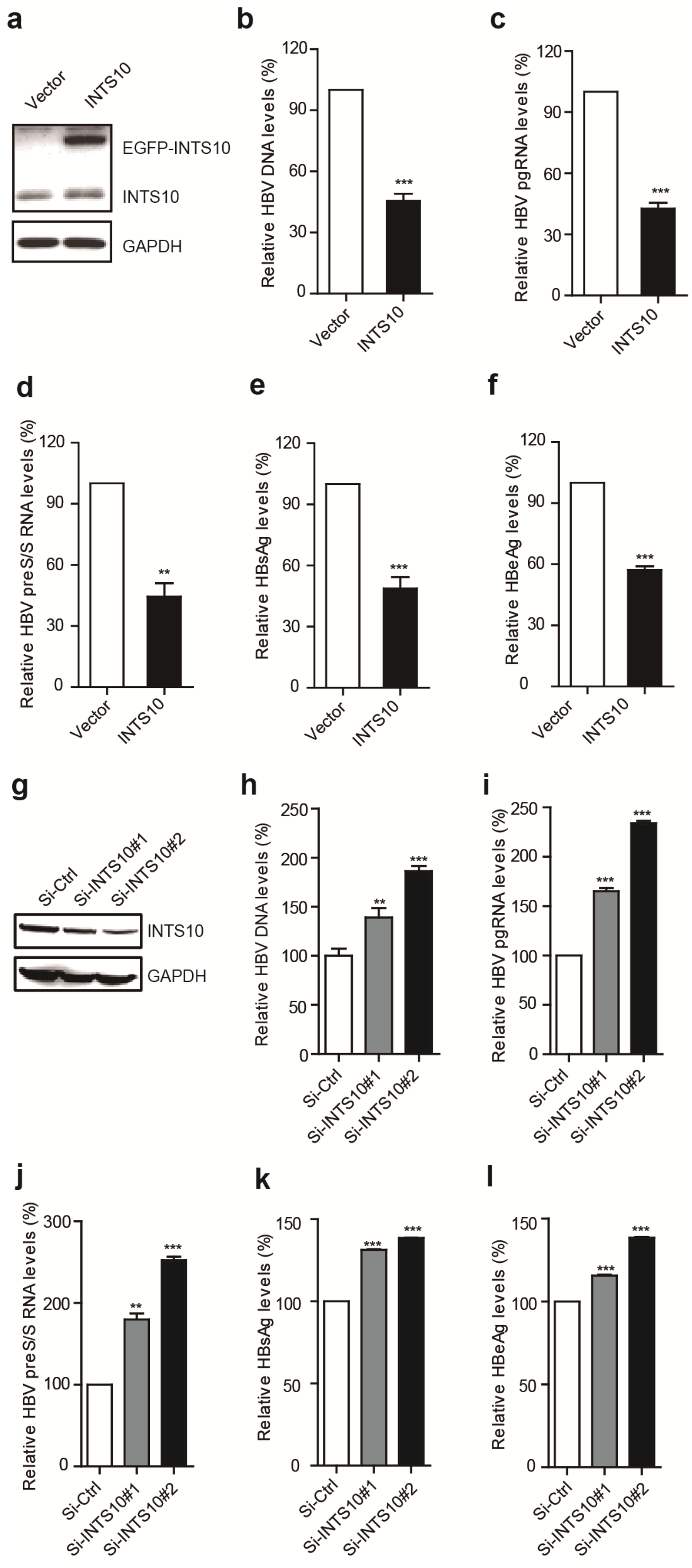

Supplementary Figure 8: INTS10 suppresses HBV replication in HepG2 cells. (a)

INTS10 levels in cellular lysates of HepG2 cells (~2×10^5) transfected with pAAV-HBV1.2 vectors, together with pLV-EGFP-INTS10 vector (INTS10) or pLV-EGFP control vector (Vector). (**b**-**d**) Levels of HBV DNAs (**b**), 3.5 Kb pregenomic RNAs (pgRNAs) (**c**) and 2.4/2.1 Kb Pre-S/S RNAs (**d**) in HepG2 cells with INTS10 overexpression. (**e**,**f**) Levels of HBsAg (**e**) and HBeAg (**f**) in supernatants of HepG2 cells with INTS10 overexpression. (**g**) INTS10 levels in cellular lysates of HepG2 cells (~2×10^5) transfected with pAAV-HBV1.2 vectors, together with INTS10-specific siRNAs (Si-INTS10#1 and Si-INTS10#2) or non-targeting scrambled siRNA controls (Si-Ctrl). (**h**-**j**) Levels of HBV DNAs (**h**), pgRNAs (**i**) and Pre-S/S RNAs (**j**) in HepG2 cells with INTS10 knockdown. (**k**,**l**) Levels of HBsAg (**k**) and HBeAg (**l**) in supernatants of HepG2 cells with INTS10 knockdown. Supernatants and cells were collected 72 hours post-transfection. INTS10 levels in cells were examined by Western blot analyses, with GAPDH indicating protein loading in each lane. Levels of HBV DNAs in cells were measured by quantitative real-time PCR (qRT-PCR). Levels of HBV RNAs in cells were measured by qRT-PCR normalized to human β-actin gene *ACTB*. The levels of HBsAg and HBeAg in supernatants were measured by enzyme-linked immunosorbent assays (ELISA). All the histograms show mean values from three independent experiments; error bars indicate standard deviation (s.d.). *P* values were determined using two-tailed unpaired *t* test. $^{*}P < 0.05$, $^{**}P < 0.01$ and $^{***}P < 0.001$.

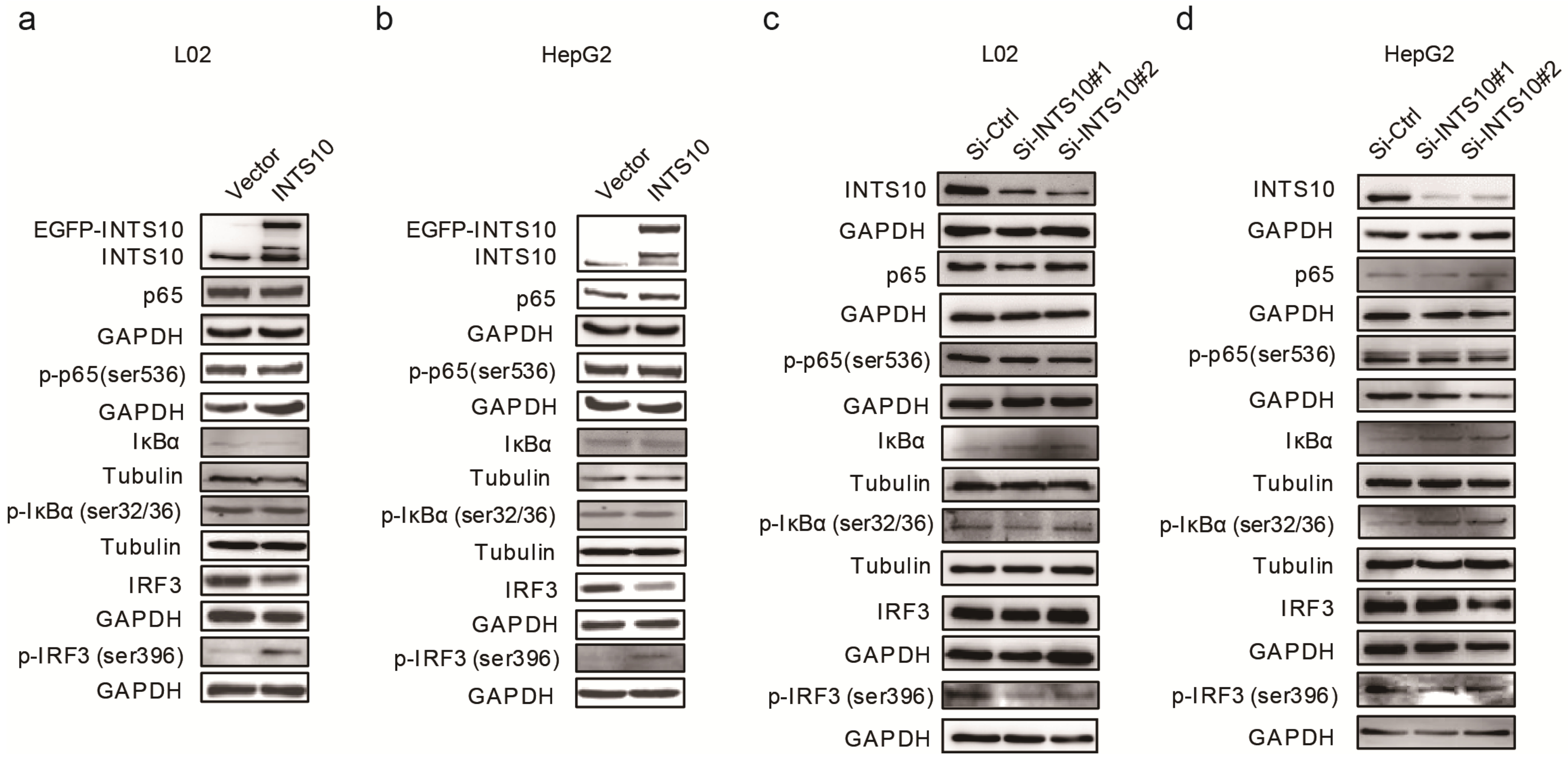
a
L02
Vector
INTS10
EGFP-INTS10
INTS10
p65
GAPDH
p-p65(ser536)
GAPDH
IκBα
Tubulin
p-IκBα (ser32/36)
Tubulin
IRF3
GAPDH
p-IRF3 (ser396)
GAPDH
b
HepG2
Vector
INTS10
EGFP-INTS10
INTS10
p65
GAPDH
p-p65(ser536)
GAPDH
IκBα
Tubulin
p-IκBα (ser32/36)
Tubulin
IRF3
GAPDH
p-IRF3 (ser396)
GAPDH
c
L02
Si-Ctrl
Si-INTS10#1
Si-INTS10#2
INTS10
GAPDH
p65
GAPDH
p-p65(ser536)
GAPDH
IκBα
Tubulin
p-IκBα (ser32/36)
Tubulin
IRF3
GAPDH
p-IRF3 (ser396)
GAPDH
d
HepG2
Si-Ctrl
Si-INTS10#1
Si-INTS10#2
INTS10
GAPDH
p65
GAPDH
p-p65(ser536)
GAPDH
IκBα
Tubulin
p-IκBα (ser32/36)
Tubulin
IRF3
GAPDH
p-IRF3 (ser396)
GAPDH

Supplementary Figure 9: INTS10 activates IRF3 pathway in L02 and HepG2 cells when exposed to HBV. (**a**,**b**) L02 cells (~2 × 10^5, **a**) or HepG2 cells (~2 × 10^5, **b**) were contransfected with pAAV-HBV1.2 vectors and pLV-EGFP-INTS10 vector (INTS10) or pLV-EGFP control vector (Vector). (**c**,**d**) L02 cells (~2 × 10^5, **c**) or HepG2 cells (~2 × 10^5, **d**) were contransfected with pAAV-HBV1.2 vectors and INTS10-specific siRNAs (Si-INTS10#1 and Si-INTS10#2) or non-targeting scrambled siRNA controls (Si-Ctrl). The levels of phosphorylated (p-) or total proteins in lysates of cell lines were measured by Western blot analyses, with GAPDH or Tubulin indicating the protein loading in each lane.

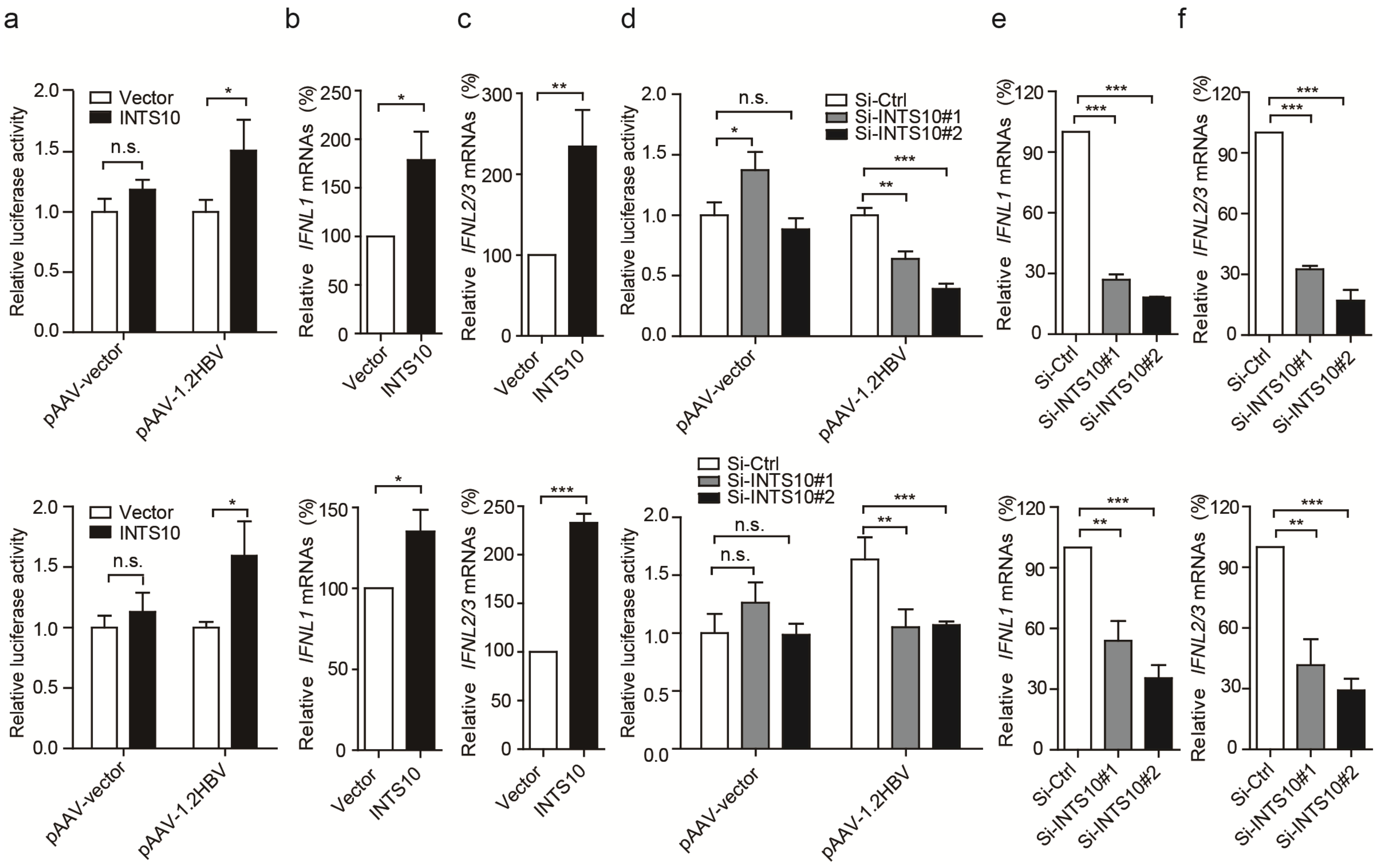

a
b
c
d
e
f
Vector
INTS10
n.s.
*
**
***
Relative luciferase activity
Relative IFNL1 mRNAs (%)
Relative IFNL2/3 mRNAs (%)
pAAV-vector
pAAV-1.2HBV
Si-Ctrl
Si-INTS10#1
Si-INTS10#2

Supplementary Figure 10: INTS10 promotes activation of ISRE reporter and elevation of mRNA levels of type III IFNs in L02 and HepG2 cells exposed to HBV. (**a**) Luciferase activity of IFN-stimulated response element (ISRE) reporter plasmids 48 hours after cotransfection into L02 (up) and HepG2 cells (down) with pLV-EGFP-INTS10 vector (INTS10) or pLV-EGFP control vector (Vector), and pAAV-HBV1.2 vector or pAAV control vector (pAAV-vector). RLU, relative luciferase units. (**b**) The mRNA levels of *IFNL1* measured by quantitative real-time PCR (qRT-PCR) in L02 (up) and HepG2 cells (down) cotransfected with pAAV-HBV1.2 vectors and INTS10 or control vectors. (**c**) The mRNA levels of *IFNL2/3* measured by qRT-PCR in L02 (up) and HepG2 cells (down) cotransfected with pAAV-HBV1.2 vectors and INTS10 or control vectors. (**d**) Luciferase activity of ISRE reporter plasmids 48 hours after cotransfection into L02 (up) and HepG2 cells (down) with INTS10-specific siRNAs (Si-INTS10#1 and Si-INTS10#2) or non-targeting scrambled siRNA controls (Si-Ctrl), and pAAV-HBV1.2 or control vectors. (**e**) The mRNA levels of *IFNL1* measured by qRT-PCR in L02 (up) and HepG2 cells (down) cotransfected with pAAV-HBV1.2 vectors and INTS10-specific or control siRNAs. (**f**) The mRNA levels of *IFNL2/3* measured by qRT-PCR in L02 (up) and HepG2 cells (down) cotransfected with pAAV-HBV1.2 vectors and INTS10-specific or control siRNAs. Human β-actin gene *ACTB* was used as reference gene in all qRT-PCR assays. All the histograms show mean values from three independent experiments; error bars indicate standard deviation (s.d.). *P* values were

determined using two-tailed unpaired *t* test. $^{*}P < 0.05$, $^{**}P < 0.01$ and $^{***}P < 0.001$.

n.s., not significant.

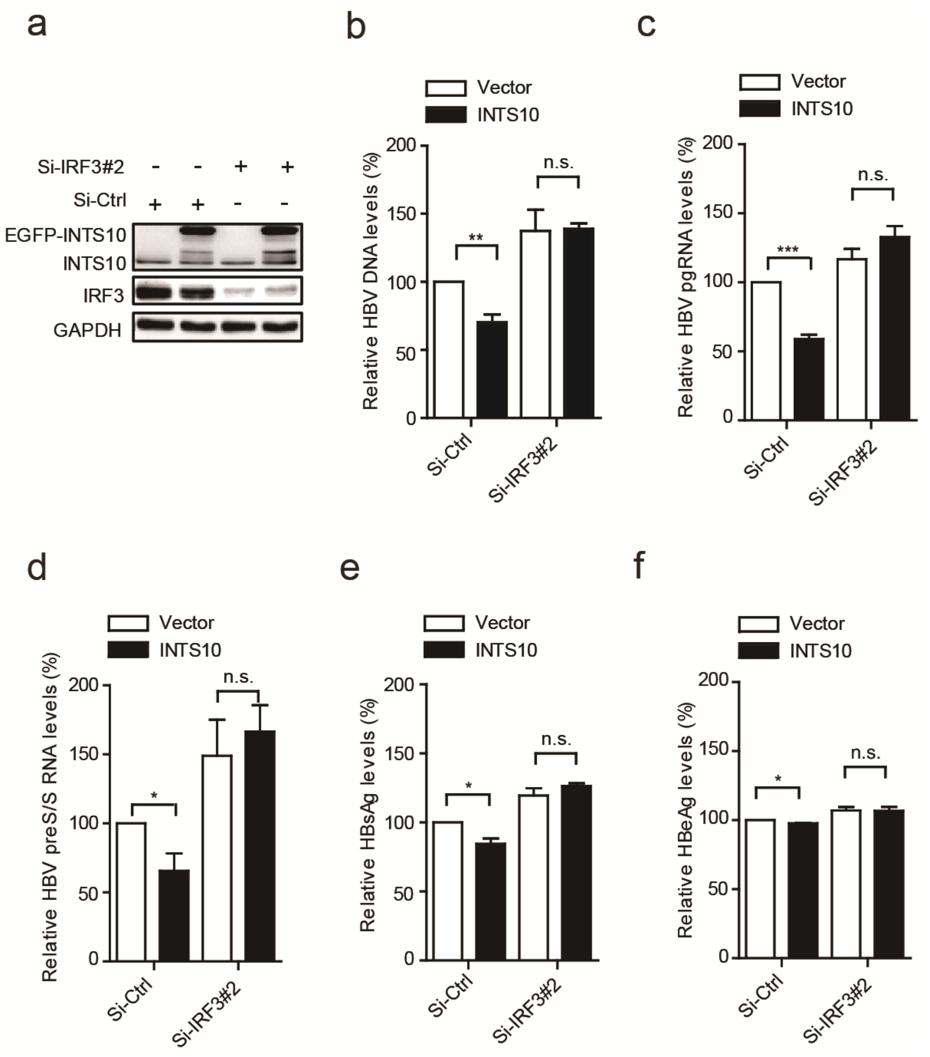

Supplementary Figure 11: INTS10 suppresses HBV replication in an IRF3-dependent manner. (**a**) Levels of INTS10 and IRF3 in HepG2.2.15 cells measured by Western blot assays. (**b**-**d**) Levels of HBV DNAs (**b**), 3.5 Kb pregenomic RNAs (pgRNAs, **c**) and 2.4/2.1 Kb Pre-S/S RNAs (**d**) in HepG2.2.15 cells stably expressing pLV-EGFP-INTS10 vector (INTS10) or pLV-EGFP control vector (Vector), which were cotransfected with IRF3-specific siRNAs (Si-IRF3#2) or non-targeting scrambled siRNA controls (Si-Ctrl). The levels of these HBV intermediates were measured by quantitative real-time PCR (qRT-PCR). (**e**,**f**) Levels of HBsAg (**e**) and HBeAg (**f**) in supernatants of HepG2.2.15 cells stably expressing INTS10 or control

vectors when cotransfected with IRF3-specific or control siRNAs. The levels were measured by enzyme-linked immunosorbent assays (ELISA). All the histograms show mean values from three independent experiments; error bars indicate standard deviation (s.d.). *P* values were determined using two-tailed unpaired *t* test. $^{*}P < 0.05$, $^{**}P < 0.01$ and $^{***}P < 0.001$. n.s., not significant.

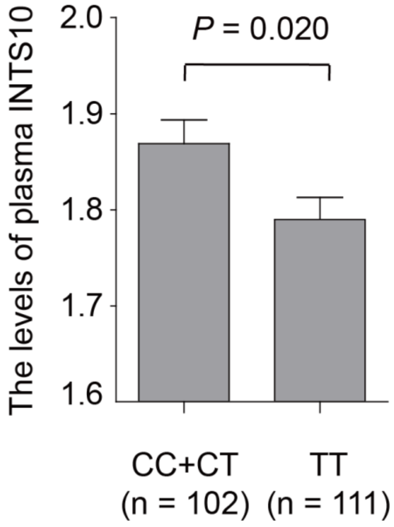

Supplementary Figure 12: Protein expression of INTS10 was markedly elevated in the plasma of rs7000921 C allele carriers. The levels of plasma INTS10 were measured in PIs from Guangdong population in replication stage 3 (n = 216) by enzyme-linked immunosorbent assays (ELISA). rs7000921 was genotyped using TaqMan assays. Totally 213 samples were used in the final analyses because 3 samples failed to be genotyped. *P* values were determined using two-tailed unpaired *t* test. Error bars indicate standard error of the mean (s.e.m.).

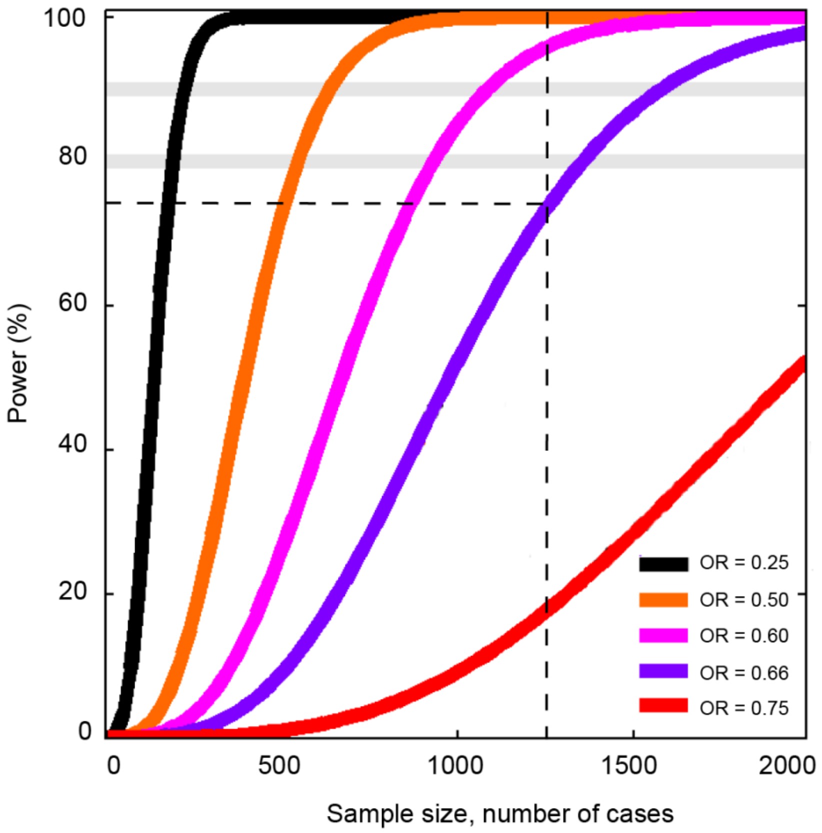

Supplementary Figure 13: Power to detect a genetic effect of various sizes (OR = 0.25, 0.50, 0.60, 0.66, or 0.75) versus study sample size. Power is reported here as the probability of SNPs to be identified in a scan. Vertical and horizontal dashed lines show that the power of our GWAS stage to identify the rs7000921 at 8p21.3 was estimated to be 75%, giving persistent HBV infection prevalence of 10%, 1,251 cases and 1,057 controls, an OR of 0.66, minor C allele frequency of 25.6% and *P* value of 2.4×10^{-6}. OR, odds ratio.

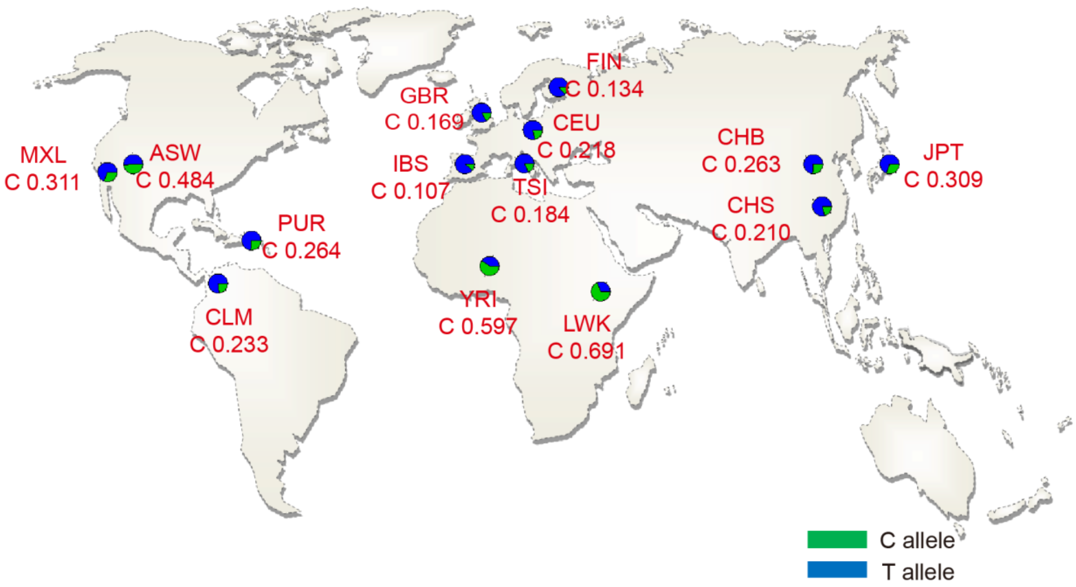

Supplementary Figure 14: Allele frequencies of the rs7000921 C allele across different populations. Frequencies of rs700021 C allele derived from the 1000 Genomes Project. The pie charts indicate the frequencies of the C (green) and T (blue) allele in each population sampled. The populations include: (1) Japanese in Tokyo (JPT); (2) Southern Han Chinese (CHS); (3) Han Chinese in Bejing (CHB); (4) Finnish in Finland (FIN); (5) Utah Residents with Northern and Western European Ancestry (CEU); (6) Toscani in Italia (TSI); (7) British in England and Scotland (GBR); (8) Iberian Population in Spain (IBS); (9) Yoruba in Ibadan (YRI); (10) Luhya in Webuy (LWK); (11) Mexican Ancestry from Los Angeles USA (MXL); (12) Americans of African Ancestry in SW USA (ASW); (13) Puerto Ricans from Puerto Rico (PUR); and (14) Colombians from Medellin (CLM).

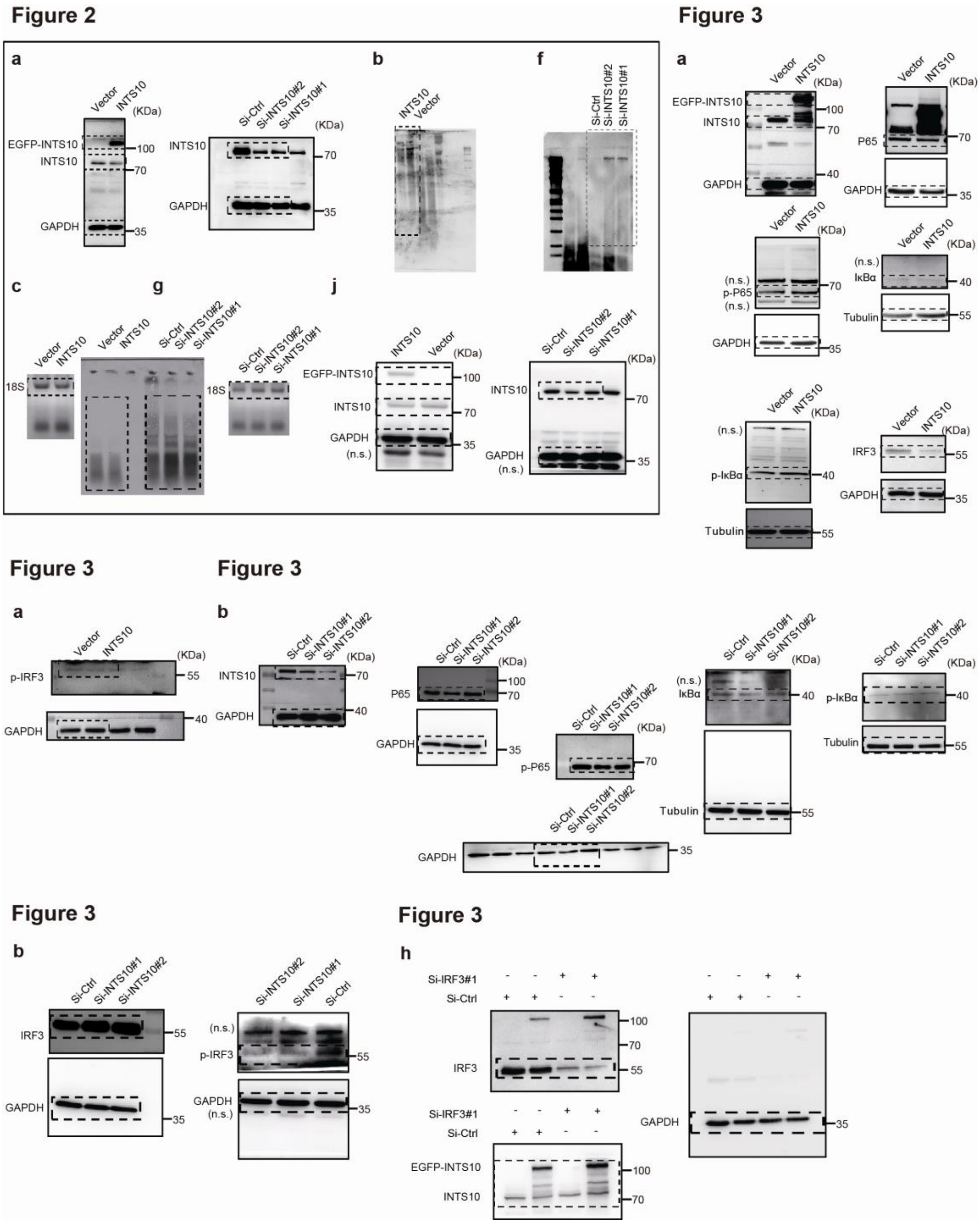

Supplementary Figure 15:Full-length pictures of the blots presented in the main figures.

Supplementary Table 1: Summary description of the samples used in this study.

(a) Samples used for the GWAS and replication studies

Characteristics	GWAS populations															
	GWAS population 1			GWAS population 2	GWAS population 3			Guangxi-GWAS population (GWAS population 1, 2 and 3)			GWAS population 4 (Jiangsu-GWAS population)			GWAS population 5 (Guangdong-GWAS population)		
	Cases (n = 286)	Controls (n = 656)	*P*	Cases (n = 707)	Cases (n = 78)	Controls (n = 74)	*P*	Cases (n = 1,071)	Controls (n = 730)	*P*	Cases (n = 91)	Controls (n = 203)	*P*	Cases (n = 89)	Controls (n = 124)	*P*
Age, years																
Mean (s.d.)	37.2 (10.1)	37.1 (10.8)	0.93	43.7 (11.6)	62.8 (8.3)	66.0 (7.2)	0.002	43.2 (12.5)	39.9 (13.6)	1.6×10^{-7}	56.6 (10.0)	57.5 (9.7)	0.46	46.3 (11.1)	47.7 (13.0)	0.42
≤ 43, n (%)	217 (75.9)	487 (74.2)	0.82	384 (54.3)	2 (2.6)	0 (0)	1.00	603 (56.3)	487 (66.7)	9.1×10^{-6}	13 (14.3)	16 (5.4)	0.14	35 (39.3)	46 (37.1)	0.85
> 43, n (%)	79 (24.1)	169 (25.8)		323 (45.7)	76 (97.4)	74 (1.0)		468 (43.7)	243 (33.3)		78 (85.7)	187 (94.6)		54 (60.7)	78 (63.9)	
Sex, n (%)																
Females	0 (0)	0 (0)	1.00	94 (13.3)	58 (74.4)	41 (55.4)	0.023	152 (14.2)	41 (5.6)	7.6×10^{-9}	17 (18.7)	61 (30.0)	0.058	23 (25.8)	33 (26.6)	0.97
Males	286 (100)	656 (100)		613(86.7)	20 (25.6)	33 (44.6)		919 (85.8)	689 (94.4)		74 (81.3)	142 (70.0)		66 (74.2)	91 (73.4)	

Characteristics	GWAS populations			Replication populations												
	Overall GWAS population			Jiangsu population (replication 1)			Guangxi population (replication 2)			Guangdong population (replication 3)			Beijing population (replication 4)		
	Cases (n = 1,251)	Controls (n = 1,057)	*P*	Cases (n = 1,279)	Controls (n = 1,360)	*P*	Cases (n = 1,299)	Controls (n = 1,067)	*P*	Cases (n = 783)	Controls (n = 560)	*P*	Cases (n = 544)	Controls (n = 369)	*P*
Age, years															
Mean (s.d.)	44.4 (12.7)	44.2 (14.6)	0.75	50.5 (11.1)	50.4 (11.0)	0.70	38.2 (10.7)	38.4 (12.4)	0.59	39.8 (14.9)	40.7 (17.0)	0.35	35.5 (11.9)	28.0 (10.5)	2.4×10^{-19}
≤ 43, n (%)	651 (52.0)	549 (51.9)	0.96	396 (31.0)	430 (31.6)	0.75	962 (74.1)	769 (72.1)	0.30	523 (67.8)	366 (65.4)	0.62	418 (76.8)	330 (89.3)	1.9×10^{-6}

> 43, n (%)	600 (48.0)	508 (48.1)		883 (69.0)	930 (68.4)		337 (25.9)	298 (27.9)		260 (33.2)	194 (34.6)		126 (23.2)	39 (10.7)	
Sex, n (%)															
Females	192 (15.3)	135 (12.8)	0.077	524 (41.0)	567 (41.7)	0.74	493 (38.0)	416 (39.0)	0.64	324 (41.4)	252 (45.0)	0.21	129 (23.7)	77 (20.9)	0.35
Males	1,059 (84.7)	922 (87.2)		755 (59.0)	793 (58.3)		806 (62.0)	651 (61.0)		459 (58.6)	308 (55.0)		415 (76.2)	292 (79.1)	

Cases, persistently hepatitis B virus infected subjects (PIs); Controls, spontaneously recovered subjects (SRs). GWAS population 1, 2 and 3 were from Guangxi province, thus their combining was referenced as "Guangxi-GWAS population". GWAS population 4 was from Jiangsu province, thus was also referred to as “Jiangsu-GWAS population”. GWAS population 5 was from Guangdong province, thus was also referred to as “Guangdong-GWAS population”. All the participants were unrelated ethnic adult Chinese. *P* values are calculated by *t* test (two-sided) for means of age, and χ^2 test (two-sided, group size > 5) or Fisher’s Exact test (two-sided, group size ≤ 5) for other variables. s.d., standard deviation.

(b) Samples randomly selected from the Guangdong population (replication 3) for detecting the plasma INTS10 by ELISA

Characteristics	Cases (n = 216)	Controls (n = 80)
Age, years		
Mean (s.d.)	40.1 (15.9)	42.6 (18.8)
≤ 43, n (%)	132 (61.1)	47 (58.7)
> 43, n (%)	84 (38.9)	33 (41.3)
Sex, n (%)		
Females	78 (36.1)	41 (51.3)
Males	138 (63.8)	39 (48.7)

ELISA, enzyme-linked immunosorbent assay. s.d., standard deviation.

(c) Random controls used for evaluating the frequency of the protective rs7000921 allele [C] in naïve controls in China

Characteristics	Random controls (n = 689)
Age, years	
Mean (s.d.)	54.9 (11.8)
≤ 43, n (%)	109 (15.8)
> 43, n (%)	580 (84.2)
Sex, n (%)	
Females	286 (41.5)
Males	403 (58.5)

s.d., standard deviation.

Supplementary Table 2: Summary of the SNPs passing quality controls in the GWAS stage.

Chr.	SNPs passing QC, n				
	GWAS population 1	GWAS population 2	GWAS population 3	GWAS population 4	GWAS population 5
1	50,933	22,922	54,690	47,664	34,293
2	48,972	23,828	55,194	48,869	36,258
3	41,014	19,998	47,471	41,222	30,565
4	36,724	18,407	42,775	37,648	27,326
5	37,457	19,135	41,764	38,722	27,670
6	45,507	19,169	48,351	38,922	30,225
7	33,693	15,342	37,583	32,678	24,593
8	33,043	16,253	36,819	33,040	25,125
9	29,206	13,468	32,564	28,715	21,528
10	33,263	16,773	36,630	32,852	23,414
11	32,143	15,351	34,113	30,697	22,095
12	31,109	14,557	34,138	28,980	21,647
13	23,094	11,127	25,757	22,942	16,902
14	20,001	9,252	22,986	19,207	14,878
15	18,898	8,354	22,431	17,907	13,668
16	19,211	8,846	23,855	18,816	13,562
17	16,991	6,523	20,594	13,913	11,855
18	18,005	8,561	21,821	17,731	13,340
19	13,197	3,802	15,329	8,409	8,185
20	15,826	7,328	17,764	15,390	11,224
21	9,288	4,271	10,485	8,823	6,759
22	9,008	3,446	11,670	7,662	6,664
Sum	616,583	286,713	694,784	590,809	441,776

Chr., chromosome. QC, quality control. GWAS population 1, 2 and 3 were from the Guangxi province, thus their combining was referred to as "Guangxi-GWAS population". GWAS population 4 was from the Juangsu province, thus was also referred to as “Jiangsu-GWAS population”. GWAS population 5 was from the Guangdong province, thus was also referred to as “Guangdong-GWAS population”. All the participants were unrelated ethnic adult Chinese. GWAS population 1 was from 1,999 cancer-free subjects in the GWAS on serum levels of complement C3 and C4, all of whom have been genotyped by Illumina Omini one array; GWAS population 2 was from the GWAS on HBV-related HCC, all of whom were genotyped

using Affymatrix SNP 5.0 array; GWAS population 3 was from 436 cancer-free subjects genotyped by Illumina Human Omni Zhonghua BeadChip (in-house unpublished data); GWAS population 4 was from the GWAS on lung cancer, all of whom have been genotyped using Affymatrix SNP 6.0 array; GWAS population 5 was from the GWAS on nasopharyngeal carcinoma, all of whom have been genotyped using Illumina Human610-Quad BeadChip (see Supplementary Methods).

Supplementary Table 3: Summary of SNPs imputation in the GWAS stage.

Chr.	SNPs (by both imputation and SNP arrays) passing QC, n		
	HapMap II	HapMap III	1000 G
1	166,887	87,196	350,308
2	187,822	87,884	377,499
3	149,547	74,158	329,225
4	137,355	65,437	330,242
5	143,811	67,774	292,516
6	158,862	71,195	312,314
7	121,770	58,366	260,077
8	127,947	57,631	255,550
9	107,286	49,603	199,565
10	118,632	56,862	237,149
11	112,079	54,508	225,260
12	106,235	52,545	215,765
13	88,509	39,771	171,304
14	72,238	35,115	148,987
15	62,923	32,593	121,037
16	59,924	32,821	134,647
17	47,944	27,943	106,370
18	65,248	31,045	131,552
19	31,263	19,298	80,716
20	52,509	27,312	98,180
21	30,747	15,162	64,790
22	28,244	14,796	51,258
Sum	2,177,782	1,059,015	4,494,311

Chr., chromosome. QC, quality control. HapMap II, imputation using the genotyping data from the HapMap phase II as reference. HapMap III, imputation using the genotyping data from the HapMap phase III as reference. 1000 G, imputation using the genotyping data from the 1000 Genomes Project as reference.

Supplementary Table 4: Comparisons between the sequencing data and the genotyping data by SNP array.

		Number of SNP genotypes determined by sequencing								
		Long-range PCR capture-based (n = 185)			Agilent array capture-based (n = 89)			All (n = 274)		
		AA	AB	BB	AA	AB	BB	AA	AB	BB
Number of SNP genotypes determined by SNP array	AA	1,805	39	0	1,147	1	0	2,952	40	0
	AB	21	1,138	2	5	829	7	26	1,967	9
	BB	0	0	215	0	0	190	0	0	405

A, reference allele. B, alternative allele. n, the number of subjects. SNP, single nucleotide polymorphism. We randomly selected 274 samples from the population 2 in the genome-wide association study (GWAS) stage, which was genotyped using Affymatrix SNP 5.0 array. We successfully determined 5,399 SNP genotypes in the 274 samples by deep sequencing. Among these genotypes, 5,324 were concordant between the array genotyping and sequencing, indicating a high rate of concordance (5,324/5,399 = 98.6%; $P < 2.2 \times 10^{-16}$, Kappa test).

Supplementary Table 5: Summary of SNPs that were significantly associated with the persistent HBV infection in previous GWASs.

No.	Chr.	Positions[a]	SNPs	Genes	Alleles[b]	ORs (95% CIs)[c]	P^c	PMID number for the studies	In our GWAS data			
									Minor allele frequencies[d]		ORs (95% CIs)[e]	P^e
									Cases (n = 1,251)	Controls (n = 1,057)		
1	6	31238572	rs1419881	*TCF19*	C/T	0.73 (0.66-0.81)	1.3×10^{-18}	23760081	0.444	0.442	1.01 (0.87-1.17)	0.92
2	6	31340090	rs3130542	*HLA-C*	A/G	0.75 (0.69-0.81)	9.5×10^{-14}	24162738	0.180	0.167	1.10 (0.89-1.35)	0.38
3	6	31959213	rs652888	*EHMT2*	C/T	1.38 (1.22-1.57)	7.1×10^{-13}	23760081	0.332	0.317	1.12 (0.96-1.31)	0.14
4	6	32022158	rs12614	*CFB*	T/C	0.53 (0.48-0.59)	1.3×10^{-34}	25802187	0.028	0.034	0.42 (0.22-0.79)	**4.0×10^{-3}**
5	6	32778233	rs2856718	*HLA-DQA2/ HLA-DQB1*	G/A	0.64 (0.53-0.78)	4.0×10^{-37}	21750111	0.385	0.443	0.81 (0.71-0.93)	**1.8×10^{-3}**
6	6	32837990	rs7453920	*HLA-DQB2*	A/G	1.81 (1.62-2.01)	6.0×10^{-28}	21750111	0.052	0.096	0.50 (0.36-0.68)	**5.5×10^{-6}**
7	6	33134224	rs2395309	*HLA-DPA1*	A/G	NA	1.6×10^{-5}	19349983	0.244	0.302	0.75 (0.63-0.89)	**1.1×10^{-3}**
8	6	33141000	rs3077	*HLA-DPA1*	T/C	0.56 (0.51-0.61)	2.3×10^{-38}	19349983	0.213	0.295	0.77 (0.65-0.91)	**2.3×10^{-3}**
9	6	33146744	rs2301220	*HLA-DPA1*	G/A	NA	4.5×10^{-6}	19349983	0.240	0.302	0.80 (0.68-0.94)	**6.5×10^{-3}**
10	6	33147603	rs9277341	*HLA-DPA1*	T/C	NA	5.1×10^{-3}	19349983	0.131	0.170	0.81 (0.66-0.99)	**3.8×10^{-2}**
11	6	33153536	rs3135021	*HLA-DPB1*	A/G	NA	3.5×10^{-3}	19349983	0.148	0.197	0.85 (0.70-1.03)	0.096
12	6	33162839	rs9277535	*HLA-DPB1*	A/G	0.57 (0.52-0.62)	6.3×10^{-39}	19349983	0.274	0.369	0.69 (0.59-0.81)	**3.8×10^{-6}**
13	6	33166930	rs10484569	*HLA-DPB1*	A/G	NA	1.6×10^{-3}	19349983	0.478	0.423	1.26 (1.09-1.45)	**1.8×10^{-3}**
14	6	33167974	rs3128917	*HLA-DPB1*	T/G	NA	1.6×10^{-4}	19349983	0.399	0.452	0.79 (0.68-0.91)	**1.4×10^{-3}**
15	6	33168096	rs2281388	*HLA-DPB1*	T/C	NA	2.6×10^{-3}	19349983	0.476	0.414	1.28 (1.11-1.48)	**8.0×10^{-4}**
16	6	33168927	rs3117222	*HLA-DPB1*	G/A	NA	2.1×10^{-4}	19349983	0.397	0.452	0.78 (0.68-0.91)	**1.3×10^{-3}**
17	6	33187144	rs9380343	*HLA-DPB1*	T/C	NA	3.2×10^{-3}	19349983	0.486	0.432	1.29 (1.12-1.50)	**4.9×10^{-4}**
18	20	44180389	rs1883832	*CD40*	T/C	1.19 (1.14-1.25)	2.95×10^{-15}	25802187	0.555	0.500	1.19 (1.05-1.34)	**6.9×10^{-3}**
19	22	20303319	rs4821116	*UBE2L3*	A/G	1.22 (1.15-1.30)	1.7×10^{-12}	24162738	0.463	0.455	1.09 (0.94-1.26)	0.25

SNP, single nucleotide polymorphism. HBV, hepatitis B virus. GWAS, genome-wide association study. Chr., chromosome. OR, odds ratio. CI, confidence interval. PMID, PubMed identification. NA, not available. [a]Positions based on NCBI Build 36. [b]Minor allele/major allele. [c]Data derived from indicated studies. [d]Imputation data using the 1000 Genomes Project data as reference. [e]*P* values, ORs and 95% CIs calculated under additive model by logistic regression while adjusting for age, sex and the first two principal components.

Supplementary Table 6: Summary of SNPs that were significantly associated with persistent HBV infection in previous candidate gene-based association studies.

No.	Chr.	Positions[a]	SNPs	Alleles[b]	Genes	PMID number for the previous studies	In our GWAS data	
							ORs (95% CIs)[c]	*P*[c]
1	1	11778965	rs1801133	A/G	*MTHFR*	18222012	0.87 (0.72-1.04)	0.12
2	1	196938276	rs4915154	G/A	*PTPRC*	15333587	1.04 (0.86-1.25)	0.70
3	1	205011484	rs1518110	C/A	*IL10*	18479293	0.96 (0.82-1.13)	0.63
4	1	205013030	rs1800872	G/T	*NA*	16824070, 20529592, 20087947	0.92 (0.76-1.10)	0.34
5	1	205013520	rs1800896	C/T	*IL10*	18479293	1.01 (0.73-1.42)	0.93
6	1	205013790	rs1800893	T/C	*IL10*	18479293	1.05 (0.75-1.45)	0.79
7	1	205105309	rs1400986	T/C	*IL20*	18479293	0.88 (0.73-1.07)	0.20
8	1	205109797	rs1518108	T/C	*NA*	18479293	1.07 (0.87-1.32)	0.51
9	2	113605371	rs380092	T/A	*IL1RN*	15188516	1.12 (0.96-1.30)	0.14
10	2	187241986	rs2290083	T/C	*ITGAV*	18694400	1.08 (0.88-1.34)	0.45
11	2	189558018	rs3106796	A/G	*COL3A1*	19000145	0.97 (0.83-1.14)	0.73
12	2	204439189	rs733618	C/T	*CTLA4*	15452244	0.92 (0.79-1.07)	0.29
13	2	204440592	rs5742909	T/C	*CTLA4*	18049163, 15452244, 16489681	1.07 (0.85-1.34)	0.57
14	2	204440959	rs231775	A/G	*CTLA4*	15452244, 18803359	1.06 (0.91-1.23)	0.48
15	2	204447164	rs3087243	A/G	*CTLA4*	15452244	1.03 (0.87-1.23)	0.65
16	2	242440994	rs10204525	A/G	*PDCD1*	20837075	NA	NA
17	3	46386939	rs1799987	A/G	*CCR5*	17063508	NA	NA
18	3	46387263	rs1799988	C/T	*CCR5*	17063508	NA	NA

19	4	100714511	rs1800591	T/G	*MTTP*	18803359	1.22 (0.97-1.54)	0.092
20	4	123597430	rs2069762	C/A	*IL2*	19938203	0.96 (0.82-1.12)	0.61
21	5	139993100	rs2569190	G/A	*CD14*	17007028	1.12 (0.96-1.30)	0.14
22	6	31650455	rs1800630	A/C	*TNF*	15649304, 12915457, 18070287, 16907849, 20825556	1.03 (0.85-1.25)	0.76
23	6	31650461	rs1799724	T/C	*LTA*	12915457, 16907849, 16127755	0.98 (0.75-1.26)	0.86
24	6	31651010	rs1800629	A/G	*TNF*	20087947, 16907849, 17612762, 12915457, 17974504, 16824070, 16827185, 20004605	1.20 (0.93-1.54)	0.16
25	6	32777351	rs11752643	T/C	*NA*	21274863	0.43 (0.15-1.22)	0.089
26	6	32919607	rs2071543	T/G	*PSMB8*	20525414, 17525827	0.78 (0.57-1.06)	0.11
27	6	32922953	rs1135216	C/T	*TAP1*	20525414, 17525827	1.05 (0.86-1.29)	0.61
28	6	33134224	rs2395309	A/G	*HLA-DPA1*	21274863, 19349983	0.75 (0.63-0.89)	$\mathbf{1.1 \times 10^{-3}}$
29	6	33141000	rs3077	A/G	*HLA-DPA1*	21274863, 19349983, 21310144	0.77 (0.65-0.91)	$\mathbf{2.3 \times 10^{-3}}$
30	6	33146744	rs2301220	C/T	*HLA-DPA1*	21274863, 19349983	0.80 (0.68-0.94)	$\mathbf{6.5 \times 10^{-3}}$
31	6	33147603	rs9277341	T/C	*HLA-DPA1*	21274863, 19349983	0.81 (0.66-0.99)	$\mathbf{3.8 \times 10^{-2}}$
32	6	33153536	rs3135021	A/G	*HLA-DPB1*	21274863, 19349983	0.85 (0.7-1.03)	0.096
33	6	33162839	rs9277535	A/G	*HLA-DPB1*	21274863, 19349983, 21310144	0.69 (0.59-0.81)	$\mathbf{3.5 \times 10^{-6}}$
34	6	33166930	rs10484569	A/G	*HLA-DPB1*	21274863, 19349983	1.26 (1.09-1.45)	$\mathbf{1.8 \times 10^{-3}}$
35	6	33167974	rs3128917	T/G	*HLA-DPB1*	21274863, 19349983	0.78 (0.68-0.91)	$\mathbf{1.3 \times 10^{-3}}$

36	6	33168096	rs2281388	A/G	*HLA-DPB1*	21274863, 19349983	1.28 (1.11-1.48)	**7.4×10^{-4}**
37	6	33168927	rs3117222	C/T	*HLA-DPB1*	21274863, 19349983	0.78 (0.67-0.91)	**1.2×10^{-3}**
38	6	33187144	rs9380343	T/C	*HLA-DPB1*	21274863, 19349983	1.29 (1.12-1.50)	**4.9×10^{-4}**
39	6	33282761	rs421446	G/A	*MIR219A1*	23508906	0.68 (0.58-0.80)	**2.6×10^{-6}**
40	6	104866020	rs8734	C/T	*CD24*	19610054	NA	NA
41	6	137582213	rs2234711	A/G	*IFNGR1*	19488747	1.12 (0.96-1.31)	0.16
42	6	152170770	rs2077647	C/T	*ESR1*	21837769	0.95 (0.81-1.11)	0.52
43	10	54201241	rs1800450	T/C	*MBL2*	20193030, 15994813, 12517417	1.03 (0.84-1.27)	0.77
44	10	54201691	rs7096206	G/C	*MBL2*	20193030, 15994813, 20712490	1.09 (0.91-1.30)	0.37
45	11	2110210	rs680	C/T	*IGF2*	16750516	1.12 (0.94-1.33)	0.20
46	11	2136445	rs3842759	A/T	*INS-IGF2*	16750516	NA	NA
47	11	102218675	rs602128	A/G	*MMP3*	18303200	1.08 (0.93-1.27)	0.32
48	11	111526126	rs549908	G/T	*IL18*	19466545	1.33 (1.08-1.65)[d]	8.3×10^{-3}
49	11	111540668	rs1946518	G/T	*IL18*	17610422, 15786533	0.88 (0.76-1.02)	0.093
50	12	4253297	rs1049606	C/T	*CCND2*	20414251	NA	NA
51	12	10436864	rs2617160	A/T	*KLRK1*	20648603	0.89 (0.77-1.03)	0.13
52	12	46525024	rs731236	G/A	*VDR*	9952386	1.06 (0.72-1.55)	0.76
53	12	66838787	rs2430561	A/T	*IFNG*	12526950, 17211638, 17033822, 17612762, 19938203	1.11 (0.90-1.35)	0.34
54	15	89212660	rs4932178	T/C	*FURIN*	19492430	1.05 (0.83-1.31)	0.70
55	17	29603901	rs1024611	G/A	*CCL2*	20087947, 17202846	0.93 (0.81-1.08)	0.34
56	17	31231893	rs2107538	T/C	*CCL5*	19017985	1.14 (0.97-1.34)	0.10

57	17	43163827	rs4794067	C/T	*TBX21*	19473434	1.08 (0.87-1.34)	0.48
58	19	10128011	rs4804490	A/C	*DNMT1*	20044957	1.01 (0.87-1.17)	0.88
59	19	10132034	rs2241531	G/C	*DNMT1*	20044957	0.99 (0.85-1.14)	0.85
60	19	46550761	rs1982073	A/G	*TGFB1*	15368436, 17974504, 15787639, 17612762	1.14 (0.99-1.33)	0.073
61	21	33536120	rs4986956	C/T	*IFNAR2*	16757563	0.82 (0.65-1.05)	0.11
62	21	33562658	rs2834167	A/G	*IL10RB*	20087947, 19714778, 16757563	0.99 (0.85-1.15)	0.90
63	21	33618577	rs2843710	G/C	*IFNAR1*	17125879	0.97 (0.82-1.14)	0.67
64	21	33618737	rs16997869	T/C	*IFNAR1*	17125879	1.04 (0.85-1.25)	0.72
65	21	33635877	rs1012335	C/G	*IFNAR1*	19103527	NA	NA
66	21	33637569	rs2257167	C/G	*IFNAR1*	19103527, 18761606	1.02 (0.88-1.18)	0.82
67	21	33698565	rs2284553	A/G	*IFNGR2*	20980339	1.08 (0.93-1.26)	0.31
68	21	33709182	rs9808753	G/A	*IFNGR2*	20980339	1.04 (0.89-1.21)	0.58
69	21	41719935	rs2071430	T/G	*MX1*	19744071, 17845304	0.87 (0.73-1.03)	0.096

SNP, single nucleotide polymorphism. HBV, hepatitis B virus. Chr., chromosome. PMID, PubMed identification. GWAS, genome-wide association study. OR, odds ratio. CI, confidence interval. NA, not available. [a]Positions based on NCBI Build 36. [b]Minor allele/major allele. [c]*P* values, ORs and 95% CIs calculated under additive model by logistic regression while adjusting for age, sex and the first two principal components. The lowest *P* values were shown based on three sets of imputation data. [d]Significant but with the opposite direction as the previously published report.

Supplementary Table 7: Associations between the HLA classical alleles and the persistent HBV infection.

No.	*HLA* classical alleles	Positions[a]	ORs (95% CIs)[b]	P^b	Linkage disequilibrium with the SNPs (r^2)			
					rs3077	rs9277535	rs2856718	rs7453920
1	*A*1101*	30019970	1.00 (0.84-1.19)	1				
2	*A*201*	30019970	0.85 (0.66-1.09)	0.19				
3	*A*206*	30019970	1.44 (0.92-2.26)	0.11				
4	*A*207*	30019970	0.74 (0.59-0.93)	**8.3×10^{-3}**				
5	*A*2402*	30019970	1.30 (1.06-1.59)	**1.2×10^{-2}**				
6	*A*3001*	30019970	1.25 (0.69-2.27)	0.46				
7	*A*3303*	30019970	1.09 (0.86-1.38)	0.49				
8	*C*102*	31346171	0.95 (0.80-1.14)	0.61				
9	*C*1402*	31346171	1.19 (0.80-1.77)	0.40				
10	*C*302*	31346171	1.18 (0.92-1.52)	0.19				
11	*C*303*	31346171	1.11 (0.75-1.63)	0.60				
12	*C*304*	31346171	0.99 (0.79-1.23)	0.92				
13	*C*401*	31346171	0.64 (0.43-0.96)	**3.0×10^{-2}**				
14	*C*602*	31346171	1.14 (0.74-1.77)	0.56				
15	*C*702*	31346171	1.06 (0.86-1.29)	0.59				
16	*C*801*	31346171	1.08 (0.84-1.38)	0.57				
17	*B*1301*	31431272	0.92 (0.7-1.22)	0.57				
18	*B*1302*	31431272	1.03 (0.55-1.92)	0.93				
19	*B*1501*	31431272	0.60 (0.33-1.07)	0.084				

20	*B*4001*	31431272	1.32 (1.03-1.68)	**2.6×10^{-2}**		
21	*B*4601*	31431272	1.01 (0.82-1.25)	0.90		
22	*B*5101*	31431272	1.00 (0.65-1.55)	1		
23	*B*5801*	31431272	1.08 (0.83-1.41)	0.58		
24	*DRB1*1101*	32660042	1.43 (0.98-2.09)	0.065		
25	*DRB1*1202*	32660042	1.25 (0.97-1.61)	0.085		
26	*DRB1*1501*	32660042	0.79 (0.61-1.01)	0.064		
27	*DRB1*301*	32660042	1.16 (0.87-1.53)	0.32		
28	*DRB1*405*	32660042	0.94 (0.66-1.33)	0.71		
29	*DRB1*701*	32660042	1.13 (0.75-1.68)	0.56		
30	*DRB1*803*	32660042	1.06 (0.71-1.58)	0.77		
31	*DRB1*901*	32660042	0.96 (0.77-1.19)	0.71		
32	*DQA1*102*	32716284	0.97 (0.81-1.17)	0.77	0.09	0
33	*DQA1*103*	32716284	1.02 (0.72-1.44)	0.92	0.04	0
34	*DQA1*201*	32716284	1.07 (0.72-1.61)	0.73	0.03	0
35	*DQA1*301*	32716284	0.50 (0.33-0.77)	**1.6×10^{-3}**	0.05	0.36
36	*DQA1*302*	32716284	0.90 (0.71-1.14)	0.39	0.13	0.01
37	*DQA1*303*	32716284	0.75 (0.50-1.13)	0.17	0.05	0
38	*DQA1*501*	32716284	1.23 (0.93-1.62)	0.15	0.14	0.01
39	*DQA1*601*	32716284	1.41 (1.09-1.82)	**9.2×10^{-3}**	0.12	0.01
40	*DQB*201*	32739039	1.16 (0.89-1.52)	0.27	0.14	0.01
41	*DQB*202*	32739039	1.13 (0.73-1.74)	0.58	0.02	0
42	*DQB*301*	32739039	1.42 (1.18-1.72)	**2.7×10^{-4}**	0	0.01

43	*DQB*302*	32739039	0.51 (0.34-0.77)	$\mathbf{1.4 \times 10^{-3}}$			0.05	0.36
44	*DQB*303*	32739039	0.96 (0.78-1.17)	0.66			0.12	0.01
45	*DQB*401*	32739039	0.89 (0.63-1.26)	0.52			0.07	0.01
46	*DQB*601*	32739039	0.80 (0.63-1.02)	0.074			0.08	0.01
47	*DQB*602*	32739039	0.82 (0.54-1.23)	0.33			0.05	0
48	*DPB1*201*	33157346	0.71 (0.57-0.90)	$\mathbf{4.8 \times 10^{-3}}$	0.38	0.32		
49	*DPB1*401*	33157346	0.81 (0.59-1.11)	0.19	0.23	0.16		
50	*DPB1*501*	33157346	0.74 (0.63-0.87)	$\mathbf{4.0 \times 10^{-4}}$	0.30	0.56		

HLA, human leukocyte antigen. HBV, hepatitis B virus. OR, odds ratio. CI, confidence interval. SNP, single nucleotide polymorphism.

[a]Positions based on NCBI Build 36. [b]*P* values, ORs and 95% CIs calculated under additive model by logistic regression while adjusting for age, sex and the first two principal components. SNPs in *HLA-DP* (rs3077 and rs9277535) and *HLA-DQ* (rs2856718 and rs7453920) were previously reported to be associated with persistent HBV infection in populations of Asia ancestry. In this study, we confirmed the associations of these 4 SNPs. Thus, we checked the linkage disequilibrium between the SNPs in *HLA-DP* (rs3077 and rs9277535) and the HLA classical allele *HLA-DPB1*, and between the SNPs in *HLA-DQ* (rs2856718 and rs7453920) and the HLA classical allele *HLA-DQ*.

Supplementary Table 8: Summary of SNPs that were significantly associated with the HBV-related phenotypes in previous GWASs.

No.	Diseases/traits	Genes	Chr.	SNPs	Alleles[a]	ORs (95% CIs) or Beta coefficient (standard error)	*P*	PMID number of the studies	In our GWAS data	
									ORs (95% CIs)	*P*
1	Disease progression of HBV infection	*GRIN2A*	16	rs11866328	T/G	0.60 (0.50-0.71)	1.6×10^{-8}	22004137	1.05 (0.86-1.29)	0.61
2	Hepatitis B vaccine response	*C2*	6	rs9267665	T/C	2.05 (1.64-2.57)	1.2×10^{-17}	21764829	1.29 (0.98-1.69)	0.074
3	Hepatitis B vaccine response	*HLA-DRA*	6	rs3135363	G/A	1.53 (1.35-1.74)	6.5×10^{-22}	21764829	1.17 (0.99-1.38)	0.074
4	Hepatitis B vaccine response	*HLA-DPB1*	6	rs9277535	A/G	0.72 (0.63-0.81)	2.9×10^{-12}	21764829	0.69 (0.59-0.81)	**3.8×10^{-6}**
5	Hepatitis B vaccine response	*HLA-DPB1*	6	rs477515	T/C	2.05 (1.75-2.41)	2.6×10^{-19}	24282030	0.69 (0.53-0.90)	**4.9×10^{-3}**
6	Hepatitis B vaccine response	*HLA-DPB1*	6	rs28366298	C/A	1.77 (1.53-2.05)	1.7×10^{-14}	24282030	0.86 (0.71-1.03)	0.10
7	Hepatitis B vaccine response	*BTNL2*	6	rs3763316	T/C	1.84 (1.56-2.17)	3.8×10^{-13}	24282030	1.03 (0.84-1.27)	0.76
8	Hepatitis B vaccine response	*HLA-DPB1*	6	rs13204672	G/A	2.01 (1.67-2.43)	1.5×10^{-13}	24282030	0.65 (0.36-1.19)	0.15
9	HBV-related HCC	*KIF1B*	1	rs17401966	G/A	0.61 (0.55-0.67)	1.7×10^{-18}	20676096	1.04 (0.89-1.23)	0.62
10	HBV-related HCC	*HLA-DQA1/ -DRB1*	6	rs9272105	A/G	1.28 (1.22-1.35)	5.2×10^{-22}	22807686	1.18 (1.00-1.39)	**4.7×10^{-2}**
11	HBV-related HCC	*GRIK1*	21	rs455804	A/C	0.84 (0.80-0.89)	5.2×10^{-10}	22807686	0.88 (0.75-1.03)	0.12
12	HBV-related HCC	*STAT4*	2	rs7574865	T/G	1.21 (1.14-1.28)	2.5×10^{-10}	23242368	0.92 (0.79-1.08)	0.32
13	HBV-related HCC	*HLA-DQ*	6	rs9275319	G/A	1.49 (1.36-1.63)	2.7×10^{-17}	23242368	0.71 (0.53-0.95)	**1.8×10^{-2}**
14	HBV-related HCC		8	rs12682266	A/G	0.72 (NR)	3.8×10^{-5}	22174901	1.01 (0.88-1.17)	0.85
15	HBV-related HCC		8	rs7821974	C/T	0.75 (NR)	2.3×10^{-4}	22174901	1.01 (0.87-1.17)	0.88
16	HBV-related HCC		8	rs2275959	A/G	1.31 (NR)	5.2×10^{-4}	22174901	0.99 (0.85-1.15)	0.90
17	HBV-related HCC		8	rs1573266	A/G	0.72 (NR)	2.7×10^{-5}	22174901	1.03 (0.87-1.21)	0.74
18	HBV/HCV-related HCC	*GLB1*	3	rs4678680	G/T	2.27 (1.68-3.08)	2.0×10^{-7}	21105107	1.21 (0.90-1.62)	0.21
19	HBV/HCV-related HCC	*BACH2*	6	rs12663434	A/C	0.60 (0.48-0.77)	3.0×10^{-5}	21105107	NA	NA

20	HBV/HCV-related HCC	*BACH2*	6	rs7749730	G/A	0.59 (0.46-0.75)	1.0×10^{-5}	21105107	0.97 (0.82-1.16)	0.78
21	HBV/HCV-related HCC	*BACH2*	6	rs9444730	G/T	0.59 (0.46-0.75)	1.0×10^{-5}	21105107	0.96 (0.80-1.15)	0.64
22	HBV/HCV-related HCC	*C2*	6	rs9267673	T/C	1.97 (1.47-2.46)	2.0×10^{-6}	21105107	1.14 (0.91-1.43)	0.26
23	HBV/HCV-related HCC	*HLA-DRB1*	6	rs2647073	C/A	1.94 (1.40-2.69)	6.0×10^{-5}	21105107	NA	NA
24	HBV/HCV-related HCC	*HLA-DRB1*	6	rs3997872	A/T	0.47 (0.33-0.67)	2.0×10^{-5}	21105107	NA	NA
25	HBV/HCV-related HCC	*C14orf143*	14	rs12100561	A/G	1.52 (1.26-1.83)	4.0×10^{-6}	21105107	1.10 (0.95-1.28)	0.16

SNP, single nucleotide polymorphism. HBV, hepatitis B virus. GWAS, genome-wide association study. Chr., chromosome. OR, odds ratio. CI, confidence interval. NR, not reported. NA, not available. PMID, PubMed identification. [a]Minor allele/major allele.

Supplementary Table 9: Primers and probes used for SNPs genotyping in the replication stage.

Samples	SNPs	Primers and probes	Sequences (5'→3')	Genotyping[c]
**By Sequenom genotyping[a]**				
		Forward	ACGTTGGATGGATCTGCTCTCATTTTCTGC	
Replication 1	rs11194095	Reverse	ACGTTGGATGGTAGAACCCTGAGGTGGAAG	S
		Extend	GGGTGGAAGGCAGATAG	
		Forward	ACGTTGGATGGGAATGATTGTAATGCTTC	
Replication 1	rs331515	Reverse	ACGTTGGATGTAAATACCCTCTTGGAGCCC	S
		Extend	ACCGTAGGTAGAGTTTG	
		Forward	ACGTTGGATGAATTAGGACTACCCATGCTG	
Replication 1	rs472918	Reverse	ACGTTGGATGTCTGACCTTCAAAGACAGCC	S
		Extend	TCACCCCACCCACCATC	
		Forward	ACGTTGGATGAGTCCTATCTAAGCTGGCAC	
Replication 1	rs12425758	Reverse	ACGTTGGATGTCAGCTATATAACCTACTC	S
		Extend	CTACTCATAACTGGGTTCT	
		Forward	ACGTTGGATGTTTTAAACACCAGCAAAGC	
Replication 1	rs7314034	Reverse	ACGTTGGATGGTTATATCTGAGTATTGGG	S
		Extend	TCGGGATTATAATGATTTTAAAACTTTT	
		Forward	ACGTTGGATGCTTATGAGTGGTTAAAGTGG	
Replication 1	rs2955360	Reverse	ACGTTGGATGGGGAGGGGTTTTCTGCAAAT	S
		Extend	TAGGGGTTTTCTGCAAATTATATGTAAT	
Replication 1	rs17525406	Forward	ACGTTGGATGAGGATTGAAAACTAGACACG	S
		Reverse	ACGTTGGATGCGCCATTTATATTGTATTCCC	

		Extend	TCCCTGAATCTTAAACATCCTTTTATG	
		Forward	ACGTTGGATGTCTTGAACACACGGACTAGG	
Replication 1	rs6071483	Reverse	ACGTTGGATGGGCTTCCTAATGTCCTTGAG	S
		Extend	CTCAACACTTCAAACATTCT	
		Forward	ACGTTGGATGCAACACTTAAGCACTCTTT	
Replication 1	rs2831544	Reverse	ACGTTGGATGGCTAGCTAGTATTAGTATAG	S
		Extend	TATTTGCCTTATTATGTGTCTA	
		Forward	ACGTTGGATGGAGAATGGACAAGCTCCAAC	
Replication 1	rs4817901	Reverse	ACGTTGGATGAAATGCACTAAAAGTGCCCG	S
		Extend	GTCCCTGCCTGCCTC	
		Forward	ACGTTGGATGTCAAGGTGATTTTGTAGGAG	
Replication 1	rs4952620	Reverse	ACGTTGGATGGGAAACATATAGCTAACTTG	S
		Extend	CATGTGAATTAAATTGGTTTATGCCTA	
		Forward	ACGTTGGATGGTATAGCTTTCCAGATACAT	
Replication 1	rs2042583	Reverse	ACGTTGGATGATGGTTACTTCTGTCTCTGG	S
		Extend	ATCTTATTTTACAATGATATGAGAGA	
		Forward	ACGTTGGATGAGCTGGAGGCATCAATCTTC	
Replication 1	rs1921824	Reverse	ACGTTGGATGTTTCAGTTCTTACAGATCC	S
		Extend	TTTTCTTATTCTGTTTACTTGCTC	
		Forward	ACGTTGGATGCATTTGGTAGGAGTTATGGC	
Replication 1	rs35056331	Reverse	ACGTTGGATGGTCACGTAACCTCAGATTCC	S
		Extend	CCCACCCTCAGATTCCCACTTG	
		Forward	ACGTTGGATGGGTCTGATAAAGTGGTTTGC	
Replication 1	rs4663581	Reverse	ACGTTGGATGGGCTCTGCGTATTATTTGAG	S
		Extend	CCCATATTATTTGCCTACATCCG	
Replication 1	rs4032301	Forward	ACGTTGGATGCTGTGTGATATTGTCTGCTG	S

		Reverse	ACGTTGGATGCACACATACACTGCCTGTAG	
		Extend	ACGCTGTCACCATTGA	
		Forward	ACGTTGGATGCTGGTACATAACAGTGGTTG	
Replication 1	rs9307564	Reverse	ACGTTGGATGTGAGTAAAAGCAGCCATTCC	S
		Extend	TCATCCATTCCAGAGAATATTAAAATC	
		Forward	ACGTTGGATGGCTAAATTTTTCTTCATCTG	
Replication 1	rs1897280	Reverse	ACGTTGGATGCAAAAATAAAATCCCTCTAC	S
		Extend	CCACTTTTATTTCAGACCACTGTTC	
		Forward	ACGTTGGATGAGGTCTTTGAGCTCTCTTCC	
Replication 1	rs9390589	Reverse	ACGTTGGATGCCAAGGAATTCAGAGAGCAC	S
		Extend	GAGCACAGAATGCTCATTATTTG	
		Forward	ACGTTGGATGATTTGGAGTGACATGGAGGC	
Replication 1	rs2466212	Reverse	ACGTTGGATGCCTATAGGTTTGATAGCTCC	S
		Extend	TTTTATGCTAAGGACCTTTATACTG	
		Forward	ACGTTGGATGTGAAGGGAGGAGTTTCAAGG	
Replication 1	rs7018262	Reverse	ACGTTGGATGCCCCTTCCCCATCTATTTAG	S
		Extend	CATCTATTTAGTCAACTATGATGAT	
		Forward	ACGTTGGATGGTCACACTGATTCTTGGGTC	
Replication 1	rs12352196	Reverse	ACGTTGGATGCATGCACAAAGCTATGCAGG	S
		Extend	GCAGGAAATTTATGTAATGTTTAG	
		Forward	ACGTTGGATGCCAAATATTTTCTCCCTGTC	
Replication 1	rs11052386	Reverse	ACGTTGGATGCATTCCACATTCAAACACTG	S
		Extend	CCACTATTTTCTCCCTGTCTGTGGCTT	
		Forward	ACGTTGGATGTGCTCAGTGACCCTAAGGGA	
Replication 1	rs1880081	Reverse	ACGTTGGATGAGGAAGCATCCAAGACAAGG	S
		Extend	GGAGACCAGTGAGATG	

		Forward	ACGTTGGATGAGGTTGTCACAGTTACAGAG	
Replication 1	rs12756662	Reverse	ACGTTGGATGAGAAGATAGACTCTGGACCG	S
		Extend	GAGCAAGTCAGGGATTTGA	
		Forward	ACGTTGGATGTGTACACCAAGGCATTGCCA	
Replication 1	rs7000921	Reverse	ACGTTGGATGAACTTCCAACAGTAGCTGCC	S
		Extend	ATTGGTGAATTCTCCAAAG	
		Forward	ACGTTGGATGACAAAGGATGTGGCTCTTGG	
Replication 1	rs7138383	Reverse	ACGTTGGATGATAAGATAAAACAATATGAC	S
		Extend	GTAGAGACTGTATCTGTGTTAAAT	
		Forward	ACGTTGGATGCCGGCCGATTTTCACCCTT	
Replication 1	rs666052	Reverse	ACGTTGGATGCTGCCATTTTGTGGAAACAG	S
		Extend	CGGCCGATTTTCACCCTTTTATAC	
		Forward	ACGTTGGATGAAACATTTGCAAACAGTGGG	
Replication 1	rs1426399	Reverse	ACGTTGGATGCCTAACACGTGAATTCTATGG	S
		Extend	AGTGGGTATGTAATAAATTACTGACTA	
		Forward	ACGTTGGATGTTGTGGAAGGAAACACAAA	
Replication 1	rs434602	Reverse	ACGTTGGATGATCCCACAAGTTAGCAGACC	S
		Extend	TTGGGGAAGGAAACACAAAACAATA	
		Forward	ACGTTGGATGTTCTCTTGTTGGGCTGTCAC	
Replication 1	rs2723437	Reverse	ACGTTGGATGATAAGGGCTCTCCCTAGTTG	S
		Extend	GACTGTGGGCCACTAA	
		Forward	ACGTTGGATGGCAAAATCTATAGAAACCC	
Replication 1	rs2225979	Reverse	ACGTTGGATGAATGAGGAAATGAAACAGCC	S
		Extend	AGCAAAATCTATAGAAACCCACTTAA	
		Forward	ACGTTGGATGAATTTAGGTGAGCTATCCAG	
Replication 1	rs7581	Reverse	ACGTTGGATGGAAGTCCTAAGGGTTTTTG	S

		Extend	GAGGGGAGCTATCCAGAGCTTTC	
		Forward	ACGTTGGATGTAGAACTTGGTCTTGGAGGG	
Replication 1	rs13301972	Reverse	ACGTTGGATGGGCATCTTGCCTTCTAATGG	S
		Extend	CTTGGAGGGCAGCTT	
		Forward	ACGTTGGATGAGAAGATCTACAGTGAAGAC	
Replication 1	rs740643	Reverse	ACGTTGGATGTAGTCTATCTGCCTGGTTGG	S
		Extend	TTACCAAACACTACTCCA	
		Forward	ACGTTGGATGAGTATGTCCTCGCATGATGG	
Replication 1	rs12745427	Reverse	ACGTTGGATGAATGTGAGGACATGGTGTTC	S
		Extend	CTCACACAGTGCCCTTAC	
		Forward	ACGTTGGATGCAATGAAAACTCCTGTTCAGA	
Replication 1	rs11009788	Reverse	ACGTTGGATGGACTGCATTAGTATCTGTATC	S
		Extend	CCTGTTCAGAAAAGTAATAGAT	
		Forward	ACGTTGGATGGAGGCATGTGGTTCGTAAAC	
Replication 1	rs35748479	Reverse	ACGTTGGATGCCTTCCCCAAGTCTGGAAAA	S
		Extend	CCCCTTCGTAAACAATATTGAAAAAAAC	
		Forward	ACGTTGGATGAAGGAAAAGCACAAAGTGCC	
Replication 1	rs6588109	Reverse	ACGTTGGATGTGGCTAAGTACTGATTCCTC	S
		Extend	AGATGCAGGATGTCAGGAAAA	
		Forward	ACGTTGGATGTGCTAGCTATTGAGCAAGTG	
Replication 1	rs28730696	Reverse	ACGTTGGATGTTTAAAACTGATAAGCCTG	S
		Extend	CTGCTTATTTAACAGCCTTATTTGATTT	
		Forward	ACGTTGGATGCACCTACTGTATTCCAGGTC	
Replication 1	rs10934369	Reverse	ACGTTGGATGGTAAGGGCCCAGTGCATTTG	S
		Extend	GCTCCAAACTCCCCA	
Replication 1	rs1993612	Forward	ACGTTGGATGGCAGTTATATATAGTGAGGAC	S

		Reverse	ACGTTGGATGAAATTAGGAGCTCCCACTC	
		Extend	GGTGAGTGCATTGTTACAG	
		Forward	ACGTTGGATGAGCAATGTGGATGCATTGCC	
Replication 1	rs926274	Reverse	ACGTTGGATGCCTCCTCACCAGGTTCATTC	S
		Extend	CCTCAGAATGGCTCCCC	
		Forward	ACGTTGGATGAGAGCCTTCCATGGGAACTC	
Replication 1	rs4729655	Reverse	ACGTTGGATGCTCTCCTCTTTCTCCTTGGT	S
		Extend	ACAGGAAACAAGCCC	
		Forward	ACGTTGGATGTGGTAACAGGCTGGCAGTG	
Replication 1	rs10116586	Reverse	ACGTTGGATGATATTGGATGCCAACTTAGG	S
		Extend	CATTGTAACAGGCTGGCAGTGTGCATCA	
		Forward	ACGTTGGATGATACCCTTCTTAGAGGACGG	
Replication 1	rs1902403	Reverse	ACGTTGGATGTCAAGCCCATTGAATTCCCC	S
		Extend	CCCGGAATTCCCCTAAAAATCGTT	
		Forward	ACGTTGGATGGGCTTATCAATAAGTTGTGTG	
Replication 1	rs6505041	Reverse	ACGTTGGATGTTTTTCTTGTTTAGTTTTGG	S
		Extend	TTTGCTTGTTTAGTTTTGGTTTAGTT	
		Forward	ACGTTGGATGAGGCAGGGAGATCCCATCG	
Replication 1	rs58331374	Reverse	ACGTTGGATGACAGAATCAATCAGCCCCAC	S
		Extend	ATAGTCGCGCTCTCC	
		Forward	ACGTTGGATGCAGTTATAAGGAGAAAGATCA	
Replication 1	rs56177624	Reverse	ACGTTGGATGGTGTACAGTTCTATGAATTT	S
		Extend	GTACAGTTCTATGAATTTTAATATACTA	
		Forward	ACGTTGGATGATGTTTTGCTTGGCTGAGTC	
Replication 1	rs449171	Reverse	ACGTTGGATGTCTCCTGACTTTACAGTGAC	S
		Extend	ACAGTGACAGAGGCTAA	

		Forward	ACGTTGGATGTGCTTTATGCATAGTACCTC	
Replication 1	rs10438527	Reverse	ACGTTGGATGTGTCCTCATTCCTAAAACAC	S
		Extend	CCTAAAACACTAGTCCCTAC	
		Forward	ACGTTGGATGGCACTATGTACTAGGATGTC	
Replication 1	rs11706404	Reverse	ACGTTGGATGCCTTTTTTATTGGGTTGTTAC	S
		Extend	TGTATTGGGTTGTTACTAGGATTA	
		Forward	ACGTTGGATGTGAGAGTCCAGGGAAAAATC	
Replication 1	rs7799945	Reverse	ACGTTGGATGGAATTATTTTGGCACCCTTG	S
		Extend	ATTGTCAAAAATCAATTAACCAT	
		Forward	ACGTTGGATGGAAAGGCTGTGGCCGAATTG	
Replication 1	rs11762272	Reverse	ACGTTGGATGTCAGGACTGGGACATGAGAG	S
		Extend	TGCACACATGGGCAG	
		Forward	ACGTTGGATGCCCTCATTAGTTAAGTCAAC	
Replication 1	chr13:106932234	Reverse	ACGTTGGATGGATCAAGGCATAGAAATATC	S
		Extend	GGTCAAGGCATAGAAATATCATAAGC	
		Forward	ACGTTGGATGCTCTGGCCCAATTTTCTGAC	
Replication 1	chr8:92704009	Reverse	ACGTTGGATGTCATCCAAGAAGACAAAGGC	S
		Extend	GACCAGAAGACAAAGGCCTTGTCACTAA	
		Forward	ACGTTGGATGGGAGGAAGACTACAGGTTTG	
Replication 1	chr2:51536515	Reverse	ACGTTGGATGCCACTTGGCCATCTTTCAAT	S
		Extend	CCACAGACAGCTGAGACTTAC	
		Forward	ACGTTGGATGGTGTGTTTGTGTATTTTAAGG	
Replication 1	rs2394534	Reverse	ACGTTGGATGCATTTAATAAATTCATACGAC	S
		Extend	TTTTGTGTATTTTAAGGTTATATCTCT	
Replication 1	rs62101819	Forward	ACGTTGGATGCAAAGTATAATTTTTGAAAAG	S
		Reverse	ACGTTGGATGCTTTAGTATGTGTTCATTTC	

		Extend	AGGTGTTCATTTCTTATTTGCAT	
		Forward	ACGTTGGATGTCTGCTGTTATGAGACTCTG	
Replication 1	rs11119687	Reverse	ACGTTGGATGTGGGTAACAGAGATCCTGTC	S
		Extend	TGATACTTTCTTCAGTATATCAATC	
		Forward	ACGTTGGATGTGTGAGGAATGCATAGAAGG	
Replication 1	rs16837192	Reverse	ACGTTGGATGTTCTCCCCAGAGCTGCTAAC	S
		Extend	GCTTTTCTGCTTCCAC	
		Forward	ACGTTGGATGTCAGGTCCACAGGGCTCAGA	
Replication 1	rs12153720	Reverse	ACGTTGGATGCAAGTAGGCAAGCATGCAAC	S
		Extend	TCCTGCGTCCTTTCTCT	
		Forward	ACGTTGGATGAACTCCAGATTTCTATCCCC	
Replication 1	rs10235363	Reverse	ACGTTGGATGTCAGACTTGGGCATGTACAG	S
		Extend	AGAGTTCTATCCCCAAATACCTACTC	
		Forward	ACGTTGGATGGAGGGCAACTTATAAGGCAG	
Replication 1	rs631408	Reverse	ACGTTGGATGCTGTGTTTTATGCACTTCCC	S
		Extend	TTTTTGTTTGTTGAATGGAC	
		Forward	ACGTTGGATGATATTCTCCCATCCTCTCC	
Replication 1	rs374725	Reverse	ACGTTGGATGTCCTGGGTAAGTTATGCTAC	S
		Extend	TGAACATTGAGCAGGA	
		Forward	ACGTTGGATGTGTATATGTATTCTTATCTG	
Replication 1	rs7220871	Reverse	ACGTTGGATGTGATAGCCTGTTCATCAAG	S
		Extend	GTTCATCAAGAAACTAGAAACTA	
		Forward	ACGTTGGATGCATTTAATGTAATTGGCTC	
Replication 1	rs41331849	Reverse	ACGTTGGATGGTAGTTAGAAAGTAGGATGGG	F
		Extend	TGCTATAAATAGTACCTCTCA	
Replication 1	rs11957374	Forward	ACGTTGGATGTTCAGAACCCTGAGATAGCC	F

		Reverse	ACGTTGGATGAGCTGCACGAACAGGATTAC	
		Extend	TGGTCAGCCAACATG	
		Forward	ACGTTGGATGAATCTCACTCCACCAAGACC	
Replication 1	rs12536200	Reverse	ACGTTGGATGCTATGTTTGAGGCCTTGTGC	F
		Extend	TGAGGGCAAAGAAAATG	
		Forward	ACGTTGGATGATGGATGAGAGTGCTGGTTC	
Replication 1	rs12218331	Reverse	ACGTTGGATGGTCATTGGAATACACCTCAC	F
		Extend	GTCATGTCCCACCAC	
		Forward	ACGTTGGATGACCTGTGAAACTGTAGAGTG	
Replication 1	rs4809766	Reverse	ACGTTGGATGATAACAGCTTCAAATTGTG	F
		Extend	GGAGGACAGCTTCAAATTGTGTTTTAC	
		Forward	ACGTTGGATGAGGAAGCATCCAAGACAAGG	
Replication 2	rs1880081	Reverse	ACGTTGGATGTGCTCAGTGACCCTAAGGGA	S
		Extend	AGGAGACCAGTGAGATG	
		Forward	ACGTTGGATGAACTTCCAACAGTAGCTGCC	
Replication 2	rs7000921	Reverse	ACGTTGGATGTGTACACCAAGGCATTGCCA	S
		Extend	ATTGGTGAATTCTCCAAAG	
		Forward	ACGTTGGATGTAGTCTATCTGCCTGGTTGG	
Replication 2	rs740643	Reverse	ACGTTGGATGAGAAGATCTACAGTGAAGAC	S
		Extend	CTTACCAAACACTACTCCA	
		Forward	ACGTTGGATGAGGTTGTCACAGTTACAGAG	
Replication 2	rs12756662	Reverse	ACGTTGGATGAGAAGATAGACTCTGGACCG	S
		Extend	AACCGATAATGCCTGCA	
**By TaqMan genotyping[b]**				
Replication 1	rs2394255	Forward	CCTGCACTATTGAATGGAGACAGT	S
		Reverse	CAGGTGAGGTCAGTGAAGTTGG	

		Probe1	(FAM)-CTGCAGCCCTACCTACAGGTGTCCTT	
		Probe2	(HEX)-CTGCAGCCCTATCTACAGGTGTCCTTC	
Replication 2	rs631408	Forward	TCTCCCCTGAACTCCAGATTTC	S
		Reverse	CTTGGGCATGTACAGCTAAATCTTT	
		Probe1	(FAM)-CAAATACCTACTCGGCA	
		Probe2	(HEX)-CCAAATACCTACTCAGC	
Replication 2	rs10235363	Forward	TCACGACCTGAATGAGACTTACATAGA	S
		Reverse	GCTTTTTAGTTTTGAGGGCAACTTA	
		Probe1	(FAM)-CAAATACCTACTCGGCA	
		Probe2	(HEX)-CCAAATACCTACTCAGC	
Replication 3 and 4	rs7000921	Forward	CAGACACATATAAAGTGTCCAGATGAG	S
		Reverse	ACATTCAACCATAAACATTCCACAT	
		Probe1	(FAM)TGTGGCTTTTCTTTC	
		Probe2	(HEX)TGGCTTGTCTTTCCA	

SNP, single nucleotide polymorphism. In the replication stage 1, 72 SNPs were selected for genotyping. Among these SNPs, one SNP (rs2394255) was within the major histocompatibility complex (MHC) region and was genotyped using TaqMan assay. The remaining 71 SNPs were genotyped using Sequenom assay. Five of these SNPs (rs41331849, rs11957374, rs12536200, rs12218331 and rs4809766) failed in the Sequenom assay. Among the 67 successfully genotyped SNPs, six SNPs (rs1880081, rs7000921, rs740643, rs12756662, rs631408 and rs10235363) survived in the replication stage 1 and went forward to the replication stage 2. In the replication stage 2, 4 SNPs (rs1880081, rs7000921, rs740643 and rs12756662) were genotyped using Sequenom assay and 2 SNPs (rs631408 and rs10235363) using TaqMan assay. Only one SNPs, i.e. rs7000921survived in the replication stage 2 and then went forward to the subsequent replication studies using TaqMan assays. [a]Primers used for genotyping using Sequenom MassArray System. [b]Primers and MGB or BHQ probes used for TaqMan assay. PCR was

performed with an initial 2 min at 50°C and 10 min at 95°C, followed by 40 cycles of 15 sec at 95°C and 1 min at 60°C. [c]S, successfully genotyped; F, genotyping failure.

Supplementary Table 10: Stratification analyses of rs7000921 by sex and age.

	Genotype	GWAS stage, n		Replication stage 1 (Jiangsu population), n		Replication stage 2 (Guangxi population), n		Replication stage 3 (Guangdong population), n		Replication stage 4 (Beijing population), n		Overall, n	
		Cases	Controls	Cases	Controls	Cases	Controls	Cases	Controls	Cases	Controls	Cases	Controls
Gender													
	T/T	610	471	424	415	459	352	225	122	226	157	1,944	1,517
Male	T/C	397	378	291	316	295	251	178	142	145	109	1,306	1,196
	C/C	52	72	39	58	42	43	29	20	20	20	182	213
OR (95% CI)		0.68 (0.57-0.82)		0.86 (0.73-1.01)		0.88 (0.74-1.05)		0.78 (0.61-1.00)		0.84 (0.64-1.10)		0.83 (0.76-0.90)	
P value		7.5×10^{-5}		0.063		0.16		0.049		0.21		1.3×10^{-6}	
	T/T	117	69	300	293	293	203	151	96	77	39	938	700
Female	T/C	67	55	190	227	164	172	136	113	40	31	597	598
	C/C	8	11	33	47	20	40	19	19	5	5	85	122
OR (95% CI)		0.50 (0.31-0.81)		0.82 (0.68-1.00)		0.62 (0.50-0.77)		0.78 (0.59-1.03)		0.67 (0.41-1.10)		0.73 (0.65-0.82)	
P value		0.0052		0.047		1.9×10^{-5}		0.080		0.11		1.1×10^{-7}	
$P_{heterogeneity}$		0.81		0.82		0.046		0.74		0.65		0.26	
Age, years													
	T/T	371	289	209	217	547	421	261	143	229	177	1,617	984
≤ 43	T/C	248	213	160	170	344	282	206	169	143	123	1,101	711
	C/C	32	47	26	41	49	61	33	25	20	24	160	154
OR (95% CI)		0.73 (0.57-0.93)		0.88 (0.71-1.09)		0.86 (0.74-1.10)		0.75 (0.60-0.94)		0.80 (0.62-1.03)		0.84 (0.76-0.93)	
P value		0.0099		0.25		0.055		0.014		0.078		6.0×10^{-4}	
> 43	T/T	356	251	515	491	205	134	115	75	74	19	1,265	1,233

	T/C	216	220	321	373	115	141	108	86	42	17	802	1,083
	C/C	28	36	46	64	13	22	15	14	5	1	107	181
OR (95% CI)		0.58 (0.45-0.74)		0.82 (0.71-0.96)		0.56 (0.43-0.74)		0.83 (0.60-1.15)		0.75 (0.40-1.44)		0.74 (0.67-0.81)	
P value		2.2×10^{-5}		0.012		2.8×10^{-5}		0.26		0.39		1.0×10^{-9}	
$P_{heterogeneity}$		0.31		0.58		0.031		0.68		0.52		0.088	

GWAS, genome-wide association study. OR, odds ratio. CI, confidence interval. The *P* values, ORs and 95% CIs were calculated under additive model by logistic regression while adjusting for age, sex and population. $P_{heterogeneity}$ values were calculated to compare the difference of ORs within each stratum of sex (female and male) or age (≤ 43 and > 43).

Supplementary Table 11: The predicted functional relevance of rs7000921 and 17 SNPs in strong linkage disequilibrium with rs7000921.

No.	SNPs	Chr.	Positions[a]	Alleles[b]	LD SNP	Population/LD (r^2)	Splicing (site)	nsSNP	Stop Codon	RegPotential[c]	Conservation[d]
1	rs10503680	8	20426247	C/G	rs7000921	CHB/0.737	Not	Not	Not	0	0
2	**rs11991803**	8	20444740	A/G	rs7000921	JPT/0.731|CHB/0.739	Not	Not	Not	0.23986	0.996
3	rs11993011	8	20440397	G/T	rs7000921	JPT/0.731|CHB/0.739	Not	Not	Not	0	0
4	rs13273637	8	20450477	A/G	rs7000921	JPT/0.766	Not	Not	Not	0	0
5	rs1471523	8	20438971	A/G	rs7000921	JPT/0.731|CHB/0.731	Not	Not	Not	0	0.002
6	rs1480351	8	20440655	G/T	rs7000921	JPT/0.731|CHB/0.731	Not	Not	Not	0	0.004
7	rs17092725	8	20433018	C/T	rs7000921	CHB/0.739	Not	Not	Not	0	0
8	rs17092760	8	20445066	G/T	rs7000921	JPT/0.731|CHB/0.739	Not	Not	Not	0.102314	0
9	rs17092766	8	20445533	C/G	rs7000921	CHB/0.739	Not	Not	Not	0	0
10	rs2127653	8	20426281	C/T	rs7000921	JPT/0.943|CHB/1.000	Not	Not	Not	0	0
11	rs2170173	8	20450380	A/G	rs7000921	JPT/0.731|CHB/0.717	Not	Not	Not	0	0
12	rs4288392	8	20428186	A/G	rs7000921	JPT/1.000|CHB/1.000	Not	Not	Not	0.089175	0
13	rs4307359	8	20447091	C/T	rs7000921	JPT/0.766	Not	Not	Not	0	0
14	rs4375017	8	20439092	C/G	rs7000921	JPT/1.000|CHB/1.000	Not	Not	Not	0	0.02
15	**rs4922214**	8	20432806	C/T	rs7000921	JPT/0.731|CHB/0.729	Not	Not	Not	0.425733	1
16	rs6980913	8	20441926	A/G	rs7000921	JPT/0.721|CHB/0.731	Not	Not	Not	NA	0
17	rs6990841	8	20445705	A/G	rs7000921	JPT/0.721|CHB/0.737	Not	Not	Not	0	0
18	rs7000921	8	20437486	C/T	rs7000921	1	Not	Not	Not	0	0

SNP, single nucleotide polymorphism. Chr., chromosome. LD, linkage disequilibrium. nsSNP, nonsynonymous SNP. JPT, Japanese in Toyko, Japan; CHB, Han Chinese in Beijing, China. [a]Positions based on NCBI Build 36. [b]Minor allele/major allele. [c]RegPotential, regulatory potential score, which was downloaded from the UCSC genome bioinformatics web site (http://genome.ucsc.edu/). [d]Conservation, ertebrate Multiz Alignment and Conservation score (17 Species), which was downloaded from the UCSC genome bioinformatics web site (http://genome.ucsc.edu/).

Supplementary Table 12: Primers used for quantitative real-time PCR assays.

Templates	Primers	Sequences (5'→3')
HBV DNA	Forward	ATCCTGCTGCTATGCCTCATCTT
	Reverse	ACAGTGGGGGAAAGCCCTACGAA
HBV pgRNA	Forward	TCTGCCTAATCATCTCTTGT
	Reverse	GAGAGAAGTCCACCACGAGT
HBV Pre-S/S RNA	Forward	TTCCTAGGACCCCTTCTCGT
	Reverse	AGCAGCAGGATGAAGAGGAA
ACTB	Forward	AGAGCCTCGCCTTTGCCGAT
	Reverse	CCATCACGCCCTGGTGCCT
IFNL1	Forward	CGCCTTGGAAGAGTCACTCA
	Reverse	GAAGCCTCAGGTCCCAATTC
IFNL2/IFNL3	Forward	AGTTCCGGGCCTGTATCCAG
	Reverse	GAGCCGGTACAGCCAATGGT

HBV, hepatitis B virus. pgRNA, pregenomic RNA. Pre-S, pre-surface-antigens; S, surface antigen. ACTB, beta-actin. Quantitative real-time PCR (qRT-PCR) was performed with an initial 3 min at 95°C, followed by 40 cycles of 3 sec at 95°C and 20 sec at 60°C. The pgRNAs and Pre-S/S RNAs of HBV in cells were measured by qRT-PCR normalized to *ACTB*.

Supplementary Table 13: Protein levels of INTS10, p-IRF3 and p-p65 in 40 liver tissues.

Samples	Age, years	Sex	HBsAg	HBcAb	Protein levels		
					INTS10	p-IRF3	p-p65
JL-01	66	Female	+	+	4	0	2
JL-02	52	Male	+	+	5	0	5
JL-03	39	Female	+	+	3	2	3
JL-04	45	Male	+	+	3	0	1.5
JL-05	52	Male	+	+	4	2	1.5
JL-06	55	Female	+	+	3	0	1.5
JL-07	58	Male	+	+	4	0	3
JL-08	49	Male	+	+	4	0	5
JL-09	37	Male	+	+	5	2.5	4.5
JL-10	48	Male	+	+	5	3	4
JL-11	49	Male	+	+	4	3	5
JL-12	45	Male	+	+	6	4	0
JL-13	48	Male	+	+	5	3	4
JL-14	49	Male	+	+	6	4	4
JL-15	61	Male	+	+	6	3	3
JL-16	46	Female	+	+	5	3	5
JL-17	46	Male	+	+	5	3	0
JL-18	48	Female	+	+	6	0	4
JL-19	56	Male	+	+	5	6	5
JL-20	44	Male	+	+	6	4	4
JL-21	41	Male	+	+	6	0	4
JL-22	62	Male	+	+	5	5	5
JL-23	51	Female	+	+	6	5	5
JL-24	61	Male	+	+	5	5	5
JL-25	50	Male	+	+	6	6	5
JL-26	50	Male	+	+	6	0	3
JL-27	56	Male	+	+	6	5	4
JL-28	57	Male	+	+	5	3	5
JL-29	43	Male	+	+	6	2.5	1.5
JL-30	39	Male	+	+	6	4	5
JL-31	46	Male	+	+	6	5	2.5
JL-32	39	Male	+	+	5	5	5
JL-33	38	Male	+	+	5	5	2.5
JL-34	53	Male	+	+	6	6	5
JL-35	49	Male	+	+	4	5	4
JL-36	56	Male	+	+	6	5	5
JL-37	45	Female	+	+	5	5	4.5
JL-38	53	Male	+	+	6	6	4

JL-39	40	Male	+	+	5	6	4.5
JL-40	29	Male	+	+	6	5	4.5

HBsAg, hepatitis B surface antigen. HBcAb, anti-hepatitis B core antigen. +, positive. Protein levels of INTS10, p-p65 and p-IRF3 were measured by immunohistochemistry (IHC) in 40 non-tumor liver tissues of patients with hepatitis B virus-related hepatocellular carcinoma collected from the Jinling Hospital (Nanjing City, China). The IHC signals were scored as following (see Methods): (1) A proportion score was assigned representing the estimated proportion of positive staining tumor cells (0, none; 1, < 1/100; 2, 1/100 to < 1/10; 3, 1/10 to < 1/3; 4, 1/3 - 2/3; 5, > 2/3); (2) Average estimated intensity of staining in positive cells was assigned an intensity score (0, none; 1, weak; 2, intermediate; 3, strong); (3) The two parameters were combined and resulting in an overall score (0 or 2 - 8); and (4) A total of 5 fields per slide were selected, counted, and averaged.

Supplementary Table 14: The allele and genotype frequencies of rs7000921 in different populations.

Populations	Sample size	Descents	rs7000921					P^{a}
			Alleles, n (%)		Genotypes, n (%)			
			T	C	TT	TC	CC	
In the present study								
Persistently HBV infected samples	5,156	Asian	7,660 (75.9)	2,432 (24.1)	2,878 (57.0)	1,904 (37.7)	264 (5.2)	
Spontaneously recovered samples	4,413	Asian	6,228 (71.7)	2,464 (28.3)	2,217 (51.0)	1,794 (41.3)	335 (7.7)	
Random controls	689	Asian	1,012 (73.4)	366 (26.6)	366 (53.1)	280 (40.6)	43 (6.2)	
In the 1000 Genomes Project (release April 2012)								
CHB	97	Asian	143 (73.7)	51 (26.3)	54 (55.7)	35 (36.1)	8 (8.2)	
CHS	100	Asian	158 (79.0)	42 (21.0)	62 (62.0)	34 (34.0)	4 (4.0)	0.20
JPT	89	Asian	123 (69.1)	55 (30.9)	39 (43.8)	45 (50.6)	5 (5.6)	
CEU	85	European	133 (78.2)	37 (21.8)	50 (58.8)	33 (38.8)	2 (2.4)	
TSI	98	European	160 (81.6)	36 (18.4)	64 (65.3)	32 (32.7)	2 (2.0)	
FIN	93	European	161 (86.6)	25 (13.4)	68 (73.1)	25 (26.9)	0 (0.0)	7.8×10^{-11}
GBR	89	European	148 (83.1)	30 (16.9)	60 (67.4)	28 (31.5)	1 (1.1)	
IBS	14	European	25 (89.3)	3 (10.7)	11 (78.6)	3 (21.4)	0 (0.0)	
YRI	88	African	71 (40.3)	105 (59.7)	14 (15.9)	43 (48.9)	31 (35.2)	
LWK	97	African	60 (30.9)	134 (69.1)	10 (10.3)	40 (41.2)	47 (48.5)	1.4×10^{-51}
ASW	61	African	63 (51.6)	59 (48.4)	19 (31.1)	25 (41.0)	17 (27.9)	
MXL	66	American	91 (68.9)	41 (31.1)	33 (50.0)	25 (37.9)	8 (12.1)	
PUR	55	American	81 (73.6)	29 (26.4)	28 (50.9)	25 (45.5)	2 (3.6)	0.64
CLM	60	American	92 (76.7)	28 (23.3)	35 (58.3)	22 (36.7)	3 (5.0)	

HBV, hepatitis B virus. CHB, Han Chinese in Beijing, China; CHS, Han Chinese South; JPT, Japanese in Toyko, Japan; CEU, Utah residents (CEPH) with Northern and Western European ancestry; TSI, Toscani in Italia; FIN, Finnish from Finland; GBR, British from England and Scotland; IBS, Iberian populations in Spain; YRI, Yoruba in Ibadan, Nigeria; LWK, Luhya in Webuye, Kenya. ASW, African Ancestry in Southwest US; MXL, Mexican Ancestry in Los Angeles, CA; PUR, Puerto Rican in Puerto Rico; CLM, Colombian in Medellin, Colombia. [a]*P* values were obtained by comparing the frequency of the C allele of rs7000921 between the spontaneously recovered samples in the present study and the populations of different descent in the 1000 Genomes Project using χ^2 test (two-sided).

Supplementary Note 1

Study samples. In the present study, we carried out a two-stage GWAS analysis, including the discovery GWAS stage and the replication stage (totally consists of 5,156 cases and 4,413 controls; Supplementary Fig. 1). In the discovery GWAS stage, the genotype data were derived from several previously published GWASs and in-house data[1-4]. These data totally consist of 12,027 individuals (including 4,262 patients with cancer and 7,765 cancer-free subjects), who have been genotyped by various genotyping platforms providing genome-wide coverage (Table 1 and Supplementary Table 1a). By screening for HBV markers, including hepatitis B surface antigen (HBsAg), antibodies against hepatitis B surface antigen (anti-HBs, HBsAb) and hepatitis B core antigen (anti-HBc, HBcAb), in the plasma of these subjects whose plasma samples were available, we determined 1,251 cases (persistently HBV infected subjects, PIs) and 1,057 controls (spontaneously recovered subjects, SRs). Patients with cancer were excluded from screening unless the cancer is probably caused by HBV infection (e.g., HBV-related HCC). Subjects who had been positive for both HBsAg and anti-HBc immunoglobulin G (IgG) for at least 6 months were defined as PIs (cases). Those who were negative for HBsAg and positive for both anti-HBs and anti-HBc IgG were defined as SRs (controls)[5]. All the individuals who were positive for anti-HBs but negative for anti-HBc IgG (may have vaccination history) were excluded. In the replication stage, four independent sample sets were included, namely the Jiangsu population, the Guangxi population, the Guangdong population and the Beijing population (Table 1 and Supplementary Table 1a). With the

same sample inclusion and exclusion criteria as those used in the discovery GWAS stage, we totally determined 3,905 cases and 3,356 controls in the replication stage (Table 1 and Supplementary Table 1a).

All the participants were self-reported Chinese and were recruited from southern (Guangxi and Guangdong province), eastern (Jiangsu province) and northern (Beijing) China, and had no serological evidence for hepatitis C virus, hepatitis D virus, and HIV coinfection. Overall, there were no significant differences between the cases and controls in terms of sex and age. However, in the GWAS population 3 (from Guangxi province), the mean age of the controls is significantly higher than that of cases (P = 0.002) and a significantly higher proportion of the cases were female compared with controls (P = 0.023); whereas in Beijing population in the replication stage, the mean age of the cases is significantly higher than that of controls ($P = 2.4 \times 10^{-19}$; Supplementary Table 1a).

GWAS populations: The GWAS population contains five independent sub-populations of Chinese ancestry. These sub-populations provided totally 1,251 cases and 1,057 controls for GWAS of persistent HBV infection.

GWAS population 1: This population contains 942 individuals (286 PIs and 656 SRs) who were identified from 1,999 cancer-free subjects in the GWAS on serum levels of complement C3 and C4, all of whom have been genotyped by Illumina Omini one array[1]. All the subjects are males and the mean ages (s.d.) of PIs and SRs are 37.2 (10.1) and 37.1 (10.8) years old, respectively.

GWAS population 2: This population contains 707 PIs who were derived from

the GWAS on HBV-related HCC, and were genotyped using Affymatrix SNP 5.0 array[2]. The GWAS on HBV-related HCC totally contains 348 PIs with HBV-related HCC and 359 cancer-free PIs, therefore genotype data of all of the 707 individuals were included in this study. The male/female ratio and the mean age (s.d.) of these PIs are 6.5 (613/94) and 43.7 (11.6) years old, respectively.

GWAS population 3: This population contains 152 individuals (78 PIs and 74 SRs), who were identified from 436 cancer-free subjects genotyped by Illumina Human Omni Zhonghua BeadChip (in-house unpublished data). The male/female ratio and the mean age (s.d.) of PIs are 0.3 (20/58) and 62.8 (8.3) years old, respectively. The male/female ratio and the mean age (s.d.) of SRs are 0.8 (33/41) and 66.0 (7.2) years old, respectively.

The above three sub-populations were all recruited from Guangxi province, thus were combined and referred to as **"Guangxi-GWAS population"**.

GWAS population 4: This population contains 294 individuals (91 PIs and 203 SRs) identified from the GWAS on lung cancer, all of whom have been genotyped using Affymatrix SNP 6.0 array[3]. The GWAS on lung cancer totally contains 5,408 individuals, among whom 2,331 were patients with lung cancer and 3,077 were cancer-free subjects. By screening for HBV markers in the plasma of the cancer-free subjects whose plasma samples were available, we determined 91 PIs and 203 SRs. Therefore genotype data of the 294 individuals were included in this study. The male/female ratio and the mean age (s.d.) of PIs are 4.4 (74/17) and 56.6 (10.0) years old, respectively. The male/female ratio and the mean age (s.d.) of SRs are 2.3

(142/61) and 57.5 (9.7) years old, respectively. This sub-population was recruited from Jiangsu province, thus was referred to as **"Jiangsu-GWAS population"**.

GWAS population 5: This population contains 213 individuals (89 PIs and 124 SRs) identified from the GWAS on nasopharyngeal carcinoma (NPC), all of whom have been genotyped by Illumina Human610-Quad BeadChip[4]. The GWAS on NPC totally contains 3,477 individuals, among whom 1,583 were patients with NPC and 1,894 were cancer-free subjects. By screening for HBV markers in the plasma of the cancer-free subjects whose plasma samples were available, we determined 89 PIs and 124 SRs. Therefore genotype data of the 213 individuals were included in this study. The male/female ratio and the mean age (s.d.) of PIs are 2.9 (66/23) and 46.3 (11.1) years old, respectively. The male/female ratio and the mean age (s.d.) of SRs are 2.8 (91/33) and 47.7 (13.0) years old, respectively. This sub-population was recruited from Guangdong province, thus was referred to as **"Guangdong-GWAS population"**.

The replication stage contains four independent case-control populations.

Jiangsu population: The Jiangsu case-control population was recruited as part of a community screening program as described previously[6]. Briefly, 9,720 subjects from the Changzhou City and 48,422 subjects from the Zhangjiagang City in Jiangsu province were screened for the HBV/HCV markers in 2004 and 2009, respectively. Totally, 865 (8.9%) PIs and 1,759 (18.1%) SRs were identified from the Changzhou City, whereas 2,156 (4.5%) PIs and 7,851 (16.2%) SRs were identified from the Zhangjiagang City. Of the 3,021 PIs in both cities, 1,279 (42.3%) have enough

high-quality DNAs for genotyping and were included in the present study. Then, we randomly selected 1,360 SRs from these two cities and matched to the PIs on age and sex. The male/female ratio and the mean age (s.d.) of PIs are 1.4 (755/524) and 50.5 (11.1) years old, respectively. The male/female ratio and the mean age (s.d.) of SRs are 1.4 (793/567) and 50.4 (11.0) years old, respectively.

Guangxi population: The Guangxi case-control population was recruited as part of a community cancer screening program for early detection of cancer conducted in the Yulin City in Guangxi province between February 2011 and October 2012. Of these subjects, 1,299 were PIs and 1,067 were SRs. The response rates for cases and controls were both 92%. The male/female ratio and the mean age (s.d.) of PIs are 1.6 (806/493) and 38.2 (10.7) years old, respectively. The male/female ratio and the mean age (s.d.) of SRs are 1.6 (651/416) and 38.4 (12.4) years old, respectively.

Guangdong population: The Guangdong case-control population contained 1,343 unrelated adult Chinese who were recruited from the outpatient clinics and hospitalization wards at the Third Affiliated Hospital, Sun Yat-sen University (Guangzhou City, China) between June 2012 and July 2014. Of these subjects, 783 and 560 were determined as PIs and SRs, respectively. The response rates for the cases and controls were 91% and 90%, respectively. The male/female ratio and the mean age (s.d.) of PIs are 1.4 (459/324) and 39.8 (14.9) years old, respectively. The male/female ratio and the mean age (s.d.) of SRs are 1.2 (308/252) and 40.7 (17.0) years old, respectively.

Beijing population: A total of 285 PIs and 215 SRs were enrolled in the

Affiliated You'an Hospital, Capital University of Medical Science (former the Second Infectious Diseases Hospital of Beijing; Beijing City, China) between November 2001 and October 2003 as described in detail previously[7]. Of these subjects, 285 PIs and 23 SRs had to be excluded because their DNAs had been depleted in the original studies. In this study, a further 265 PIs were recruited from October 2005 to July 2010 at this hospital and 279 PIs were recruited from January 2008 to November 2009 at the Beijing 302 Hospital (a specialized infectious disease hospital; Beijing City, China); therefore, the Beijing case-control population in the present study had a total of 544 PIs. The response rate for PIs was 89%. A further 177 SRs were recruited as controls from October 2005 to July 2010 at the Affiliated You'an Hospital, Capital University of Medical Science; therefore, the Beijing case-control population in the present study had a total of 369 SRs. The response rate for SRs was 85%. All the 544 PIs and 369 SRs were unrelated ethnic adult Chinese and residents in the Beijing City and the surrounding regions in China. The male/female ratio and the mean age (s.d.) of PIs are 3.2 (415/129) and 35.5 (11.9) years old, respectively. The male/female ratio and the mean age (s.d.) of SRs are 3.8 (292/77) and 28.0 (10.5) years old, respectively.

SNP imputation. To increase the number of overlapping SNPs among data sets and generate more genotypes in the discovery GWAS stage, we performed imputation on the GWAS data sets using a Markov Chain based haplotyper (MACH; version 1.0.16)[8] with haplotypes derived from genotypes of 90 samples of Asian ancestry (CHB + JPT) in the HapMap phase II

(http://hapmap.ncbi.nlm.nih.gov/downloads/phasing/2006-07_phaseII/phased/), 170 samples of Asian ancestry (CHB + JPT) in the HapMap phase III (http://hapmap.ncbi.nlm.nih.gov/downloads/phasing/2009-02_phaseIII/HapMap3_r2/) and initial low coverage sequencing data of 62 samples of Asian ancestry (CHB + JPT) in the 1000 Genomes Project (ftp://ftp.1000genomes.ebi.ac.uk/vol1/ftp/pilot_data/release/2010_03/pilot1/). Before imputation, all the SNPs were checked for strand inconsistencies. For the Affymetrix data, the publicly available NetAffx annotation files provide the necessary strand information (if not always error-free) to 'flip' alleles to the forward strand. For the Illumina data, few SNPs with A/T and C/G alleles were genotyped. For convenience, we removed all these SNPs to avoid spurious imputation results. For all the datasets, data were imputed by a two-stage design. The first stage generated error and crossover maps as parameter estimates for imputation on a random subset of 200 samples or all samples per study over 100 iterations of the initial statistical model. We used these parameter estimates to generate maximum likelihood estimates of allele numbers per SNP on the basis of reference haplotypes for the datasets during the second stage of the imputation. Each data set was analyzed separately because genotyping was carried out on different platforms. For all the data sets, cases and controls were imputed together. The SNPs with imputation r^2 less than 0.30 (for the HapMap project) or 0.50 (for the 1000 Genomes Project) as indicated by MACH were excluded from analyses of the datasets, because imputed genotypes below this threshold are probably of poor quality. Imputation r^2 is an estimation of the expected

correlation between imputed genotypes and actual genotypes. The SNPs with MAF < 0.01 were also excluded from further analyses. We then combined “allele dosages” (the estimated number of minor alleles from MACH, ranging from 0 to 2) from each population together. The SNPs that were genotyped or imputed in only one population were excluded.

Assessment of accuracy of array genotyping and imputation in the GWAS stage. We randomly selected 274 samples from the GWAS population 2 in the GWAS stage (more than ten percent of the samples) to repeat the array genotyping and imputation results by deep sequencing. We used long-range PCR and Agilent array, respectively, to capture a ~127-Kb non-repeat genomic region (Chr. 1: 10153049-10408606 at 1p36.22 locus, based on NCBI Build 36) in 185 and 89 samples, respectively, and then resequenced the captured products using deep sequencing. We removed sequencing adaptors in reads and low quality reads containing more than five unknown bases, and then aligned the remaining reads to human genome (hg19 build) using BWA (v0.5.9) with default parameters. SAMtools (v0.1.8) was used to remove PCR duplicates. For long-range PCR based sequencing, we achieved a mean depth of 284.0-fold, with more than 83.8% of the regions covered at least 10-fold. For Agilent array based sequencing, we achieved a mean depth of 676.2-fold, with more than 97.6% of the regions covered at least 10-fold. A SNP was considered to be heterozygous when both alleles were present in > 15% of all reads. When assessing the array genotyping and imputation data accuracy, we only compared the SNPs

covered sufficiently (≥ 8 ×) at the genomic position by deep sequencing. When accounting for the array genotyping accuracy, we evaluated the concordance between the genotypes determined by array genotyping and those determined by deep sequencing. We successfully determined 5,399 SNP genotypes in the 274 samples by deep sequencing. Among these SNPs, 5,324 were concordant between the array genotyping and sequencing, indicating a high rate of concordance (5,324/5,399 = 98.6%). Kappa test was used to evaluate the agreement between these two platforms. When accounting for the imputation accuracy, we calculated the Pearson's correlation coefficients between the "allele dosages" from the imputed data based on the 1000 Genomes Project and the non-reference allele proportions of SNPs in the deep sequencing.

Genome-wide genetic association analyses in the GWAS stage. We combined all five GWAS populations (GWAS population 1-5) and then conducted joint association analyses. Population substructure was characterized using principal component analyses as implemented in EIGENSTRAT (version 3.0)[9]. We used a random set of ~25,000 independent autosomal SNP markers ($r^2 < 0.001$) that passed quality control criteria after imputation. Using the 206 reference samples in HapMap phase II, we confirmed that all the cases and controls in this study were of Chinese ancestry (Supplementary Fig. 3). We used basic covariates of component vectors 1 and 2 from principal components analyses (PCA) to adjust statistical models for covariates accounting for possible population substructure.

We did genome-wide association analyses at every SNP using MACH2DAT[10] by use of imputation results based on HapMap phase II, HapMap phase III and 1000 Genomes Project data, respectively. To account for imputation uncertainty, we used "allele dosages" as a primary predictor of persistent HBV infection in logistic regression models adjusted for age, sex and admixture principal components. The Manhattan plot of $-\log_{10}(P)$ was generated using Haploview (v4.2). The quantile-quantile plot was generated using R package to evaluate the overall significance of the genome-wide associations and the potential impact of population stratification. If a SNP was successfully imputed by different references, we selected the association result with the lowest *P* value.

Selection of SNPs for the replication studies. In the joint analyses for case-control populations in the GWAS stage, a locus was chosen for replication when it had at least one SNP with a *P* value $\leq 1.0 \times 10^{-4}$ in the GWAS stage. Among these loci, SNPs with a *P* value of $\leq 1.0 \times 10^{-4}$ were selected for subsequent analyses. To test whether the selected SNPs were independent associations, we conducted conditional analyses by adjusting the most significant SNP at each locus. The SNPs showed significant associations ($P \leq 0.05$) in conditional analyses were considered as additional signals in this locus. We then selected the SNPs showing independent association within each region as candidates for subsequent replication studies. We gave priority to genotyped SNPs rather than imputed ones, because it is possible that even low error rates of imputation could have considerable effects on downstream analyses[11]. If a candidate

SNP cannot be designed conveniently using MassARRAY Assay Design 3.0 software (Sequenom), we will genotype another candidate SNP in the same region. These steps led to the identification of 72 candidate SNPs forward to the replication stage (Supplementary Data 1).

In addition to performing the joint analyses to select SNPs for replication, we also conducted independent GWAS for each case-control populations and subsequently performed meta-analysis in the GWAS stage. We identified 43 candidate loci containing SNPs with $P \leq 1.0 \times 10^{-4}$, which were fully within the 72 loci identified by joint analysis (data not shown). Thus, we selected all of the 72 loci for the replication studies.

Genotyping and quality controls in the replication stage. In the replication stage 1, 72 SNPs were genotyped (Supplementary Data 1). Among these SNPs, one SNP (rs2394255) was within the major histocompatibility complex (MHC) region and was genotyped using TaqMan assays. The remaining 71 SNPs were genotyped using Sequenom assays, but five of them failed. Among the 67 successfully genotyped SNPs, six SNPs survived in the replication stage 1 ($P < 0.05$ and with effects in the same direction as in the GWAS stage) and went forward to the replication stage 2. In the replication stage 2, four SNPs (rs1880081, rs7000921, rs740643 and rs12756662) were genotyped using Sequenom assays and two SNPs (rs631408 and rs10235363) using TaqMan assays. Only one SNP, i.e. rs7000921, survived in the replication stage 2 and then went forward to the subsequent replication studies (i.e. replication stage 3

and 4) using TaqMan assays.

In the Sequenom assays, approximately 15 ng of genomic DNA was used to genotype each sample. Locus-specific PCR and detection primers were designed using the MassARRAY Assay Design 3.0 software (Sequenom). The sample DNAs were amplified by multiplex PCR, and the products were then used for locus-specific single-base extension reaction. The resulting products were desalted and transferred to a 384-element SpectroCHIP array (Sequenom). Allele detection was performed using MALDI-TOF-MS (Sequenom). The mass spectrograms were analyzed by the MassARRAY TYPER software. TaqMan assays were performed according to the manufacturers' instructions (Applied Biosystems). For the primers and probes used for the genotyping of these SNPs, see Supplementary Table 9. The genotype data in the replication studies were subjected to the same quality control analyses as in the GWAS stage. The cluster patterns of the genotyping data from the Sequenom and TaqMan assays were visually checked to confirm their good quality.

In the replication stage, the associations were carried out with additive model using PLINK (v1.07). Meta-analysis of data generated from multiple stages was conducted to assess the pooled genetic effects. Furthermore, we calculated the Cochran's Q statistic to test for between-group heterogeneity. The heterogeneity was considered significant when $P < 0.05$. These analyses were performed using meta-analysis helper (METAL) software[12].

The potential modification effects of sex and age on the association between rs7000921 and persistent HBV infection risk were assessed both by adding interaction

terms in the logistic regression model and by separate analyses of subgroups of subjects stratified by these factors.

Genotype-expression analyses. We performed correlation analyses between the SNPs genotypes and the mRNA expression levels to evaluate whether a SNP serves as an expression quantitative trait locus (eQTL) with putative *cis* regulatory effects on gene expression traits in liver tissues. The first dataset was from our in-house database (data not shown). Briefly, thirty-one non-tumor liver tissues from 31 subjects with HBV-related HCC were collected from May 2007 to July 2008 at the First Affiliated Hospital of Zhejiang University (Hangzhou City, China; n = 13) and the Peking University Cancer Hospital and Institute (Beijing City, China; n = 18), respectively. The male/female ratio and the mean age (s.d.) of these 31 subjects are 5.2 (26/5) and 52.7 (8.8) years old, respectively. The genotypes of these 31 subjects and the mRNA expression profiles of tissues from these 31 subjects were generated using the Affymetrix Genome-Wide Human SNP Array 6.0 and the Affymetrix Human Exon 1.0 ST arrays (the mRNA expression profiles data were already available, microarray GEO number: GSE74925), respectively, according to the manufacturer's instructions (Affymetrix, USA). The genotyping of rs7000921 failed in one sample, thus the eQTL analyses were performed on the remaining 30 individuals. The mRNA expression levels were log2 transformed. A second independent sample set[13] was used to replicate the eQTL signals from the first 31 samples. This sample set includes 88 non-tumor liver tissues from 88 subjects with HCC recruited from the Hong Kong

Queen Mary Hospital (Hong Kong, China)[13]. Among these 88 HCCs, 81 are HBV-related ones. The male/female ratio and the mean age (s.d.) of these 88 subjects are 3.6 (69/19) and 55.0 (11.1) years old, respectively. The genotypes of rs7000921, rs11991803 and rs4922214 in these 88 subjects were obtained by whole genome sequencing from the European Genome-phenome Archive database (accession number ERP001196). The INTS10 mRNA expression levels of these 88 samples were detected using Rosetta/Merck Human RSTA Affymetrix 1.0 microarray (GEO dataset accession number GSE25097). The mRNA expression levels were log2 transformed. Three subjects were identified as outliers (the mRNA levels of *INTS10* > mean + 3 s.d. or < mean - 3 s.d.), thus the eQTL analyses were performed on the remaining 85 individuals. The intensity of all gene array experiments were normalized together using the Robust Multiarray Averaging (RMA) method.

Functional annotations of genes at 8p21.3. Six genes (*CSGALNACT1*, *INST10*, *LPL*, *SLC18A1*, *ATP6V1B2* and *LZTS1*) are located within 1 megabase (Mb) from rs7000921. *CSGALNACT11* is involved in the elongation and initiation of chondroitin sulfate synthesis[14]. *INTS10* is a subunit of the integrator complex, which is functionally relevant with spliceosome and mediates transcriptional initiation, pause release and transcriptional termination[15-20]. *LPL* contributes to normal lipoprotein metabolism, tissue-specific substrate delivery and utilization[21]. *SLC18A1* encodes the vesicular amine transporter 1, which is important for the functioning of monoaminergic systems[22]. *ATP6V1B2* encodes a component of vacuolar ATPase that

mediates acidification of endosomal organelles[23]. *LZTS1*, also known as *FEZ1*, is a candidate tumor suppressor gene[24]. According to the RNA-Seq Atlas database, INTS10 is expressed in a wide range of tissue types including in liver tissues. Among the other genes in 8p21.3, the expression of *CSGALNACT1* in liver tissue is low; *LPL* is mainly expressed in heart and lung tissues but low in liver; the expression of *SLC18A1* is very low in various types of tissues, including liver; the expression of *ATP6V1B2* is very high in various types of tissues, but much lower in liver; *LZTS1* is expressed mainly in liver and heart tissues.

Functional annotations of candidate SNPs at 8p21.3. Functional annotations of rs7000921 and SNPs which are tagged by rs7000921 (linkage disequilibrium $r^2 > 0.7$) was performed using multiple tools, including the SNPinfo Web Server (http://snpinfo.niehs.nih.gov/)[25] and HaploReg (http://www.broadinstitute.org/mammals/haploreg/haploreg.php)[26]. Seventeen SNPs were suggested to be tagged by rs7000921 ($r^2 > 0.7$), of which rs11991803 and rs4922214 are in conserved regions predicted to have high regulatory potential scores according to the SNPinfo Web Server (RegPotential score = 0.24 and 0.43, respectively; Supplementary Table 11). HaploReg further showed that the rs11991803 was within a transcriptional repressor CCCTC-binding factor (CTCF)-binding site detected in multiple cell types including the human hepatoma cell line HepG2, suggesting that this variant might be involved in gene regulation (Supplementary Fig. 7).

Pathway enrichment analyses based on gene expression profiles of liver tissues after *INTS10* dysregulation. Gene expression profiles of non-tumor liver tissues from three independent data sets were used to perform pathway enrichment analyses after *INTS10* dysregulation. Data set 1 consists of 31 mRNA expression profiles of liver samples by the Affymetrix Human Exon 1.0 ST arrays (Affymetrix, USA) from 31 patients with HBV-related HCC (in-house data). Data set 2 consists of 289 mRNA expression profiles of liver samples by Rosetta/Merck Human RSTA Affymetrix 1.0 microarray (Affymetrix, USA) from 289 patients with HBV-related HCC (GSE25097). Data set 3 consists of 96 mRNA expression profiles of liver samples by Rosetta/Merck Human RSTA Custom Affymetrix 1.0 microarray (Affymetrix, USA) from 96 patients with HBV-related HCC (GSE22058). In each data set, we divided the samples into two subgroups according to the mean expression level of *INTS10* mRNA. The up and down quartile were defined as INTS10-high and INTS10-low subgroups, respectively. Then, the significance analyses of microarrays (SAM) were performed to search for genes highly differentially expressed between these two subgroups. We defined genes with Q value < 0.01 and $|\text{fold change}| > 1.2$ as highly differentially expressed genes. Then, the DAVID tool (Version 6.7) was used for pathway enrichment analyses based on the highly differentially expressed genes.

Cell transfections. The immortalized human hepatocyte cell line L02, the human hepatoma cell line HepG2, and the human hepatoma cell line HepG2.2.15 which

constitutively produces HBV were maintained in our lab. All cell lines were confirmed to be negative for mycoplasma contamination. These cell lines are not listed in the database of commonly misidentified cell lines maintained by International Cell Line Authentication Committee (ICLAC). The HBV-producing plasmid pAAV-HBV1.2, which contains 1.2 mer genome-length HBV sequences, was provided by Prof. Li Tang (Beijing Proteome Research Center, Beijing City, China). The following siRNAs targeting *INTS10* or *IRF3* were used: Si-INTS10#1: 5'-GGCUGAAACUAUUGAAGAATT-3', Si-INTS10#2: 5'-GCUCUCAGAAGUACAUAAUTT-3'. Si-IRF3#1: 5'-GCCACUGGUGCAUAUGUUCTT-3', Si-IRF3#2: 5'-GGAGGCAGUACUUCUGAUATT-3'. A non-targeting scrambled siRNA (Si-Ctrl, 5'-UUCUCCGAACGUGUCACGUTT-3') was used as control. The cDNAs of *INTS10* were subcloned into the pLV-EGFP or pCMV-myc vectors (Promega, Madison, WI, USA), according to manufacturer's instruction. For gene silencing, the cells were transfected with siRNAs using the riboFect™ CP (RiboBio Co. Ltd., Guangzhou City, China) as instructed by the manufacturer. For transfection with pLV-EGFP-INTS10 vector (INTS10) or pLV-EGFP control vector (Vector), FuGENE® HD was used as instructed by the manufacturer (Fugent, LLC, USA). Seventy-two hours after transfection, protein extracts were analyzed by Western blot analyses to confirm the protein knockdown or overexpression.

Western blotting assays. Cells were lysed with ice-cold lysis buffer (20 mmol/L

Tris-HCl, pH 7.5, 150 mmol/L NaCl, 1 mmol/L Na2EDTA, 1 mmol/L EGTA, 1% Triton, 2.5 mmol/L sodium pyrophosphate, 1 mmol/L β-glycerophosphate, 1 mmol/L Na3VO4, 1 μg/mL leupeptin, and protease inhibitor cocktail) for 30 minutes (min) in ice. Cell lysates were then collected after centrifugation at 12,000 rpm for 5 min at 4°C. Sixty micrograms of lysate protein were loaded and total cellular protein was separated with 15% SDS-PAGE and then transblotted overnight at 4°C onto Hybond-P polyvinylidene difluoride membrane (Amersham Biosciences). The membrane was probed with indicated primary antibodies at room temperature for 1 hour (h) and then washed three times with 0.1% Tween 20-TBS and incubated in a horseradish peroxidase-linked secondary antibody for 1 h at room temperature. The membrane was washed three times with 0.1% Tween 20-TBS and the immunoreactive bands were detected by using enhanced chemiluminescent plus reagent kit. The following primary antibodies were used in this study: anti-GAPDH (GeneSci, ZL9001), anti-Tubulin (GeneSci, #ZL9003), anti-INTS10 (Proteintech, #15271-1-AP), anti-IRF3 (Proteintech, #11312-1-AP), anti-p-IRF3 (ser396; Cell Signaling Technology, #4947), anti-p65 (SAB, #21014-2), anti-p-p65 (ser536; SAB, #11014-2), anti-IκBα (Santa, #sc-203) and anti-p-IκBα (ser32/36; Santa, #sc-101713).

Detection of HBV DNAs and RNAs. The HBV DNAs were purified from intracellular core particles as described[27], then were subjected to Southern blot analyses using AP-labeled HBV DNAs as the probes generated with the Gene Images AlkPhos Direct labeling system (Amersham Pharmacia Biotech). In a separate

experiment, the levels of HBV DNAs were also measured by quantitative real-time PCR (qRT-PCR) in the iQ5 real-time PCR detection system (BioRad). HBV DNA load in plasma was determined using the TaqMan assay according to the manufacturer's instructions (COBAS TaqMan HBV Test version 2.0, Roche Diagnostics, Indianapolis, IN, USA). RNA templates were extracted from indicated cells using the Trizol Reagent (Invitrogen, USA). Then, the RNAs were subjected to Northern blot analyses using AP-labeled HBV DNAs as the probes. In a separate experiment, the levels of HBV 3.5 Kb pregenomic RNAs (pgRNAs) and 2.4/2.1 Kb Pre-S/S RNAs were also measured by qRT-PCR in the iQ5 real-time PCR detection system (BioRad).

qRT-PCRs. cDNAs were made from RNA templates using a standard reverse transcriptase protocol (iScript, BioRad) by use of 500 ng of RNA per reaction. qRT-PCR for HBV DNAs and RNAs, *IFNL1* and *IFNL2/IFNL3* was carried out in triplicate by the SYBR Green method on an iQ5 real-time PCR detection system (BioRad) using iQ SYBR Green Supermix (BioRad catalog n° 170-8862). PCR primers and probes were designed using Primer3 (v.0.4.0, http://bioinfo.ut.ee/primer3-0.4.0), as listed in the Supplementary Table 12. Normalization for RNA quantity was performed with primers of human β-actin gene *ACTB* for each sample using the $^{\Delta\Delta}$Ct method. Final abundance figures were adjusted to yield an arbitrary value of 100% for cells expressing control vectors and/or non-targeting scrambled siRNA controls. Melting curve analyses were performed on

all PCRs to rule out non-specific amplification.

Enzyme-linked immunosorbent assays (ELISAs). To identify persistently HBV infected subjects (PIs) and spontaneously recovered subjects (SRs) in the discovery GWAS stage and replication stage, hepatitis B surface antigen (HBsAg), antibodies against hepatitis B surface antigen (anti-HBs, HBsAb) and hepatitis B core antigen (anti-HBc, HBcAb) in the plasma/serum of these subjects were detected using ELISA kits from Kehua Bio-Engineering Co., Ltd. (Shanghai City, China), according to the manufacturer's instructions. Ten percent of the plasma/serum samples were randomly selected for repeated assays, and the results were 100% concordant. To measure HBV protein markers *in vitro* functional experiments, the levels of HBsAg and hepatitis B e antigen (HBeAg) in cell culture supernatants were detected using ELISA kits from Kehua Bio-Engineering Co., Ltd. (Shanghai City, China). All the assays detecting the levels of HBsAg and HBeAg in cell culture supernatants were carried out in triplicate, and each experiment was carried out at least 3 times. To compare the concentration of INTS10 in PIs with that in SRs and evaluate correlation between INTS10 and HBV DNA load, INTS10 in the plasma of 216 PIs and 80 SRs were detected using ELISA kits from CUSABIO BIOTECH Co., Ltd. (Wuhan City, China). Although the process of how INST10 is transported into serum is almost unknown at present, its existence in plasma as suggested by the ELISA was confirmed with an independent rabbit polyclonal anti-INTS10 (Proteintech, #15271-1-AP) by Western blotting assays (data not shown).

Luciferase reporter gene assays. The IFN-stimulated response element (ISRE) reporter, which contains sites recognized by p-IRF3 and/or p-IRF7, was purchased from Promega (#E4141; Madison, WI, USA). Liver cells were plated at a density of 5 × 10^4 cells/well in 24-well plates, and transiently cotransfected with the reporter gene, siRNAs, vectors, and pRLTK (Promega), which expresses Renilla luciferase and was used to normalize the transfection efficiency, using Lipofectamine™ 2000 (Invitrogen). After 48 h, the cells were washed and lysed, and a Dual-Glo™ Luciferase Assay System (Promega) was used to determine the luciferase activity according to the manufacturer's instructions. The fold induction of promoter activity was calculated by dividing the relative luciferase activity of stimulated cells with that of Lipofectamine-treated cells. All the assays were carried out in triplicate, and each experiment was carried out at least 3 times.

Immunohistochemistry (IHC). Two slides of each biopsy were stained with hematoxylin-eosin for routine histological evaluation. The slides were washed in xylene to remove the paraffin and then rehydrated through serial dilutions of alcohol, followed by washings with a solution of phosphate-buffered saline (PBS, pH 7.2). All subsequent washes were buffered via the same protocol. The slides were then incubated with 3% H_2O_2 for 10 min to reduce non-specific staining. Treated slides were placed in a citrate buffer (pH 6.0) and heated in a pressure cooker for 2 min. The slides were then incubated for overnight at 4°C with three primary antibodies

separately, including rabbit polyclonal anti-INTS10 (Proteintech, #15271-1-AP), rabbit monoclonal anti-p-IRF3 (ser396; Cell Signaling Technology, #4947) and rabbit polyclonal anti-p-p65 (ser536; SAB, #11014-2). After washing, the slides were treated by the MaxVisionTM HRP-Polymer anti-Rabbit IHC Kit (Maxim Co., China). Then all slides were stained with 3, 3-diaminobenzidine tetra-hydrochloride (DAB) and the cells were counter-stained with hematoxylin. Negative controls and positive controls were performed at the same time. The slides were mounted with gum for examination and capture by the Olympus BX51 microscopic/Digital Camera System for study comparison. The immunohistochemistry signals were scored as previously described[28,29]. Briefly, a proportion score was assigned representing the estimated proportion of positive staining tumor cells (0, none; 1, < 1/100; 2, 1/100 to < 1/10; 3, 1/10 to < 1/3; 4, 1/3 - 2/3; 5, > 2/3). Average estimated intensity of staining in positive cells was assigned an intensity score (0, none; 1, weak; 2, intermediate; 3, strong). The two parameters were combined and resulting in an overall score (0 or 2 - 8). A total of 5 fields per slide were selected, counted, and averaged. Slides were scored by two pathologists (Li P and Li Y) who did not have knowledge of ligand-binding assay results or patient outcome. The correlations of protein levels between INTS10 and p-IRF3, or between INTS10 and p-p65 were assessed by a Spearman’s test. A *P* value of < 0.05 was used as the criterion of statistical significance, and all statistical tests were two sided.

Supplementary References

1. Yang, X. et al. Genome-wide association study for serum complement C3 and C4 levels in healthy Chinese subjects. *PLoS Genet* **8**, e1002916 (2012).

2. Zhang, H. et al. Genome-wide association study identifies 1p36.22 as a new susceptibility locus for hepatocellular carcinoma in chronic hepatitis B virus carriers. *Nat Genet* **42**, 755-8 (2010).

3. Hu, Z. et al. A genome-wide association study identifies two new lung cancer susceptibility loci at 13q12.12 and 22q12.2 in Han Chinese. *Nat Genet* **43**, 792-6 (2011).

4. Bei, J.X. et al. A genome-wide association study of nasopharyngeal carcinoma identifies three new susceptibility loci. *Nat Genet* **42**, 599-603 (2010).

5. Deng, G. et al. Association of estrogen receptor alpha polymorphisms with susceptibility to chronic hepatitis B virus infection. *Hepatology* **40**, 318-26 (2004).

6. Hu, L. et al. Genetic variants in human leukocyte antigen/DP-DQ influence both hepatitis B virus clearance and hepatocellular carcinoma development. *Hepatology* **55**, 1426-31 (2012).

7. Ma, J. et al. Association of NKG2D genetic polymorphism with susceptibility to chronic hepatitis B in a Han Chinese population. *J Med Virol* **82**, 1501-7 (2010).

8. Li, Y., Willer, C.J., Ding, J., Scheet, P. & Abecasis, G.R. MaCH: using sequence and genotype data to estimate haplotypes and unobserved genotypes. *Genet Epidemiol* **34**, 816-34 (2010).

9. Price, A.L. et al. Principal components analysis corrects for stratification in genome-wide association studies. *Nat Genet* **38**, 904-9 (2006).

10. Li, Y., Willer, C., Sanna, S. & Abecasis, G. Genotype imputation. *Annu Rev Genomics Hum Genet* **10**, 387-406 (2009).

11. Huang, L., Wang, C. & Rosenberg, N.A. The relationship between imputation error and statistical power in genetic association studies in diverse populations. *Am J Hum Genet* **85**, 692-8 (2009).

12. Willer, C.J., Li, Y. & Abecasis, G.R. METAL: fast and efficient meta-analysis of genomewide association scans. *Bioinformatics* **26**, 2190-1 (2010).

13. Sung, W.K. et al. Genome-wide survey of recurrent HBV integration in hepatocellular carcinoma. *Nat Genet* **44**, 765-9 (2012).

14. Gotoh, M. et al. Enzymatic synthesis of chondroitin with a novel chondroitin sulfate N-acetylgalactosaminyltransferase that transfers N-acetylgalactosamine to

glucuronic acid in initiation and elongation of chondroitin sulfate synthesis. *J Biol Chem* **277**, 38189-96 (2002).

15. Yamamoto, J. et al. DSIF and NELF interact with Integrator to specify the correct post-transcriptional fate of snRNA genes. *Nat Commun* **5**, 4263 (2014).

16. Baillat, D. et al. Integrator, a multiprotein mediator of small nuclear RNA processing, associates with the C-terminal repeat of RNA polymerase II. *Cell* **123**, 265-76 (2005).

17. Matera, A.G. & Wang, Z. A day in the life of the spliceosome. *Nat Rev Mol Cell Biol* **15**, 108-21 (2014).

18. Skaar, J.R. et al. The Integrator complex controls the termination of transcription at diverse classes of gene targets. *Cell Res* **25**, 288-305 (2015).

19. Stadelmayer, B. et al. Integrator complex regulates NELF-mediated RNA polymerase II pause/release and processivity at coding genes. *Nat Commun* **5**, 5531 (2014).

20. Gardini, A. et al. Integrator regulates transcriptional initiation and pause release following activation. *Mol Cell* **56**, 128-39 (2014).

21. Wang, H. & Eckel, R.H. Lipoprotein lipase: from gene to obesity. *Am J Physiol Endocrinol Metab* **297**, E271-88 (2009).

22. Lawal, H.O. & Krantz, D.E. SLC18: Vesicular neurotransmitter transporters for monoamines and acetylcholine. *Mol Aspects Med* **34**, 360-72 (2013).

23. Hinton, A., Bond, S. & Forgac, M. V-ATPase functions in normal and disease processes. *Pflugers Arch* **457**, 589-98 (2009).

24. Ishii, H. et al. FEZ1/LZTS1 gene at 8p22 suppresses cancer cell growth and regulates mitosis. *Proc Natl Acad Sci U S A* **98**, 10374-9 (2001).

25. Xu, Z. & Taylor, J.A. SNPinfo: integrating GWAS and candidate gene information into functional SNP selection for genetic association studies. *Nucleic Acids Res* **37**, W600-5 (2009).

26. Ward, L.D. & Kellis, M. HaploReg: a resource for exploring chromatin states, conservation, and regulatory motif alterations within sets of genetically linked variants. *Nucleic Acids Res* **40**, D930-4 (2012).

27. Ono, S.K. et al. The polymerase L528M mutation cooperates with nucleotide binding-site mutations, increasing hepatitis B virus replication and drug resistance. *J Clin Invest* **107**, 449-55 (2001).

28. Tse, K.P. et al. MCP-1 Promoter Polymorphism at 2518 is associated with metastasis of nasopharyngeal carcinoma after treatment. *Clin Cancer Res* **13**, 6320-6 (2007).

29. Harvey, J.M., Clark, G.M., Osborne, C.K. & Allred, D.C. Estrogen receptor status

by immunohistochemistry is superior to the ligand-binding assay for predicting response to adjuvant endocrine therapy in breast cancer. *J Clin Oncol* **17**, 1474-81 (1999).
